# In Vitro Models for Investigating Intestinal Host–Pathogen Interactions

**DOI:** 10.1002/advs.202306727

**Published:** 2023-12-28

**Authors:** Reece McCoy, Sophie Oldroyd, Woojin Yang, Kaixin Wang, Darius Hoven, David Bulmer, Matthias Zilbauer, Róisín M. Owens

**Affiliations:** ^1^ Department of Chemical Engineering and Biotechnology University of Cambridge Cambridge CB3 0AS UK; ^2^ Wellcome‐MRC Cambridge Stem Cell Institute University of Cambridge Cambridge CB2 0AW UK; ^3^ Department of Pharmacology University of Cambridge Cambridge CB2 1PD UK

**Keywords:** in vitro models, pathogens, gut microbiome, organoids, tissue engineering

## Abstract

Infectious diseases are increasingly recognized as a major threat worldwide due to the rise of antimicrobial resistance and the emergence of novel pathogens. In vitro models that can adequately mimic in vivo gastrointestinal physiology are in high demand to elucidate mechanisms behind pathogen infectivity, and to aid the design of effective preventive and therapeutic interventions. There exists a trade‐off between simple and high throughput models and those that are more complex and physiologically relevant. The complexity of the model used shall be guided by the biological question to be addressed. This review provides an overview of the structure and function of the intestine and the models that are developed to emulate this. Conventional models are discussed in addition to emerging models which employ engineering principles to equip them with necessary advanced monitoring capabilities for intestinal host‐pathogen interrogation. Limitations of current models and future perspectives on the field are presented.

## Graphical abstract

Infectious diseases are increasingly recognized as a major threat worldwide due to the rise of antimicrobial resistance and the emergence of novel pathogens. In vitro models that can adequately mimic in vivo gastrointestinal physiology are thus in high demand. This review provides an overview of such models and their applicability for interrogating host‐pathogen interactions.

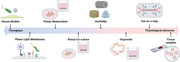

## 1 Introduction

The intestinal tract serves as an intricate ecosystem with vital complex functions. Comprising the small and large intestines, the organs are specialized in digestion, absorption of nutrients, regulation of immune responses, and maintenance of overall body homeostasis. The healthy intestine is a dynamic interface with a functioning symbiotic relationship between the gut microbiome and the host epithelium.^[^ ^1^ ^]^ Diseases have been associated with perturbations of the microbiome – termed dysbiosis.^[^ ^2^, ^3^ ^]^

Host‐pathogen interactions are often intricate and poorly understood processes involving the interplay between the human host and various pathogens – including bacteria, viruses, fungi, and parasites. Such interactions routinely have harmful outcomes that are dependent on the virulence factors of pathogens and the efficacy of the host's immune defenses. Studying the mechanisms which underpin such interactions is important for designing effective strategies to prevent and treat intestinal infections and associated diseases.

**Figure 1:**
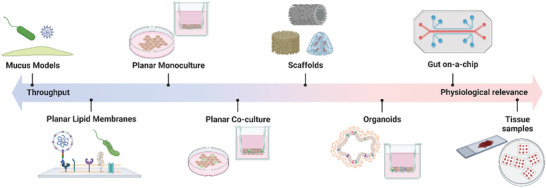
Range of in vitro models for investigating intestinal host pathogen interactions ordered by throughput and physiological relevance. Created in Biorender.com.

While in vivo animal models continue to be used extensively to investigate host‐pathogen interactions, they often suffer from being expensive and time‐consuming^[^ ^4^ ^]^ The lack of biological relevance to humans is increasingly under question, and there are significant doubts about the extrapolation of findings to humans. To overcome this, human tissue derived in vitro models have emerged as a promising alternative for studying host‐pathogen interactions (**Figure** **1**). At their simplest they involve the cultivation of epithelium and immune system‐derived cells in controlled conditions that aim to mimic the in vivo physiological environment. These systems enable emulation of intestinal structure and function to examine host‐pathogen interactions in a simple yet controlled system. Specific aspects of host‐pathogen interactions, including adhesion, invasion and the corresponding immune response can be assessed, often simultaneously. Such models accelerate the pace of research by enabling high throughput experimentation.

This literature review will discuss how the structure and function of the intestine are fundamentally interdependent on each other and detail the cellular composition which gives rise to physical characteristics that mediate host‐pathogen interactions. Traditional and emerging in vitro models are explored, and their strengths and limitations discussed. In vivo models will only be discussed insofar as they inform the design of physiologically relevant in vitro models. Ex vivo methodologies will be addressed as a stepping‐stone between in vitro and in vivo models. A significant emphasis is placed on organoids and organoid‐derived models. By critically evaluating the current literature, we aim to provide a valuable insight into the future directions of in vitro modelling and its potential impact on the mechanistic understanding of, and development of, preventive and therapeutic approaches for intestinal infections.

## 2 The Intestine

### 2.1 Structure and Function

**Figure 2:**
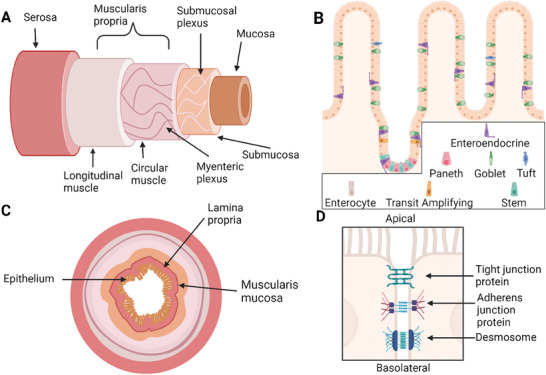
Structure of the small intestine. A) Side‐on view of the layers. B) Zoomed in view of the jejunum epithelial layer that makes up one part of the mucosa, along with the lamina propria and the muscularis mucosae. C) Cross sectional view of the layers. D) Enterocyte structure within epithelial layer, with a barrier formed due to adherens junction proteins, tight junction proteins and desmosomes. Figure created in Biorender.com

The gastrointestinal (GI) tract is composed of two main sections: the small intestine, which includes the duodenum, jejunum, and ileum, and the large intestine, comprising the cecum, colon, rectum, and anus. The function of the intestine is to absorb nutrients and water from food, via the small intestine, and to digest food into stool, via the colon. The structural arrangement of the GI tract includes four layers: the mucosa, submucosa, muscularis (consisting of circular and longitudinal muscles), and serosa (**Figure** **2**A,C). Between these layers, two neuronal plexuses, namely the submucosal plexus and the myenteric plexus, are interspaced. The mucosa itself is composed of three layers: the epithelium, the lamina propria, and the muscularis mucosa.

The intestinal epithelium plays a crucial role in protection against disease, digestion of food and absorption of nutrients by serving as a selectively permeable barrier that facilitates the exchange of materials between the external and internal environments. It physically and chemically segregates these environments while also mediating signals between the microbiome and the immune system.^[^ ^5^ ^]^ The epithelium is composed of several different cell types shown in Figure 2B and outlined in more detail in **Table** **1**. The epithelium is made primarily of barrier‐forming enterocytes held together by the apical junction complex consisting of tight and adherens junction proteins, and desmosomes^[^ ^6^ ^]^ shown in Figure 2D. The basement membrane exists to separate the epithelial cells from the lamina propria, providing extracellular matrix structural support to the cells as well as aiding in filtration.^[^ ^7^ ^]^ The tight junctions play a significant role in regulating gut barrier transport and maintaining epithelial paracellular resistance, allowing small non‐polar molecules to cross.^[^ ^8^ ^]^ The adherens junctions and desmosomes play a mechanical role in cellular communication and adhesion.^[^ ^9^ ^]^

**Table 1:** Common cell types within the intestine.

Cell type	Function	Relevance to host pathogen interactions	Characteristics	Location	Abundance	Ref
Enterocyte	Absorb nutrients and function as a physical barrier between the intestinal lumen and bloodstream.	Prevent the physical passing of pathogens.	Cells are held together by tight junctions, adherence junctions and desmosomes. Cells are polarised, with apical boundary facing the lumen. Tall and columnar, with an elongated nucleus.	Epithelial lining of colon and small intestine (SI). More in small intestine than larger intestine.	Up to ≈80% of all epithelial cells.	[23, 24]
Goblet	Produce mucus, which provides lubrication in the lumen, and acts as a physical barrier.	Prevents the physical passing of pathogens. Secretes anti‐microbial proteins. Can form antigen passages useful for immune response.	Narrow base, with wider top. Cells are polarized, with the apical boundary facing the lumen and with nucleus at the basal end.	Epithelial lining of colon and SI.	A gradual increase in abundance further through the GI tract, with less than 5% in the duodenum and more than 15% in the distal colon.	[25, 26]
Paneth	Regulation of the microbiome through secretion of granules containing antimicrobial peptides and growth factors.	Pathogenic stimulation causes release of granules, reducing the impact of viruses.	Polarised, slightly triangular shaped columnar cells.	Base of the crypts, along the epithelial lining of the SI.	The number in a single crypt decreases from an average of 18 in the ileum to seven in the duodenum. ∼3%‒8%.	[25, 26]
Enteroendocrine	Responsible for the sensing of luminal contents and secretion of hormones. There are at least 12 distinct types of cells involved in the regulation of motility, digestion, and satiety.	Chemosensory receptors can detect pathogens and release cytokines and hormones to modulate an immune response.	Microvilli containing, columnar cells. Polarised cells with apical microvilli, facing the lumen.	Sparsely scattered among epithelial barriers.	Less than 1% of total epithelial population.	[27, 28, 29]
Tuft	Chemosensory cells that act as sentinels, with ability to sense between bitter and sweet.	Suggested to be involved in the immune response for helminth and protozoan parasites	Tuft shaped microvilli, polarised cells.	Sparsely scattered among epithelial barriers.	Less than 0.4‐2% of total epithelial population.	[30, 31]
M cells	Enable the transcytosis of microbial antigens.	Antigens are transferred to the lymphoid structures. Enables reception of antigens by dendritic cells, initiating the immune response.	Polarised cell, no microvilli, apical membrane has a microfold that cleaves in the centre of the cell.	Located in the follicle‐associated epithelium of the Peyer's patch in the small intestine, and in the colonic patches and other lymphoid structures.	Less than 1% of the total epithelial population.	[32]
Stem cells	Can differentiate into any of the intestinal epithelial cells. Upon dividing they produce a new stem cell and a daughter TA cell.	Allow for the rapid regeneration of the intestinal barrier upon damage by pathogens.	Polarised cells.	Located at the bottom of intestinal crypts.	Between 1‐6 stem cells are present in each crypt.	[33, 34]
Transit amplifying cells	Migrate upwards through the intestinal crypt during differentiation from stem cells.	Allow for the rapid regeneration of the intestinal barrier upon damage by pathogens. Changing the proliferation rate changes secretory/absorptive balance.	Rapid proliferation rate. Physical characteristics change upon differentiation. Divide every 12‐16 h. Live in crypts for 1 for 2 days. Divide up to 6 times.	Located on sides of intestinal crypts.	They generate 300 cells per crypt per day.	[18]
Macrophages	Release proinflammatory cytokines. Involved in the recognition and phagocytosis of foreign bodies. Help initiate the immune response through presentation of antigens.	Help regulate gut inflammation during the presence of pathogens and commensal bacteria while also avoiding chronic and excessive inflammation.	Round or oval shaped, ovoid nucleus, diameter between 10 to 30 µm. White blood cell.	Located in lamina propria, under the epithelial layer.	Most abundant of all the immune cells present in the lamina propria.	[35, 36]
T cells	T cells are activated by antigens, either directly or via dendritic cells.	Exhibit a tolerance to commensal bacteria and are used to defend against pathogens. Type b exhibit a low level of baseline activity to prevent an uncontrolled response.	White blood cell. Has T‐cell receptors on the cell surface, these have constant, variable and transmembrane regions made of 2 polypeptide chains.	Located in lamina propria and as small populations of intraepithelial lymphocytes. Type a (CD4 or CD8αβ coreceptors) T cells exist in the lamina propria, and type b exist in the epithelium (CD8αα homodimers).	‐	[37, 38]
B cells	Generate IgA producing plasma cells. Memory B cells are used for immune protection.	Antibody producing cells fight against infection, with antibodies binding to specific antigens and promoting their clearance.	White blood cell. Has B‐cell receptors on the surface, these have constant, variable and transmembrane regions made of 4 peptides.	Located in lamina propria, specifically in the germinal centres of the GALT.	The plasma cells generated are the largest population of antibody producing cells in the body, ≈6 × 10^10^.	[39]
Dendritic	Transport antigens to lymph nodes, activate T cells and initiate immune response.	Determine whether activate an immune response to a pathogen.	White blood cell. Antigen presenting. Irregular shape, with spindles increasing the surface area to volume ratio, irregular nucleus.	Located in lamina propria in GALT.	‐	[40]

The function of the epithelial barrier is strongly dependent on its ability to maintain cellular polarity. All barrier cells are polarized, with distinct apical (luminal) and basolateral (basement membrane) regions.^[^ ^10^ ^]^ The membrane of each side is composed of different proteins and lipids, essential to the homeostasis of the gut. The apical side of enterocytes cells contains microvilli, proteins associated with absorption, and acts to defend against harmful pathogens.^[^ ^11^ ^]^ Within the basolateral membrane exist cell adhesion molecules for connection to the basement membrane.

The epithelium also contains goblet cells responsible for producing mucus, a hydrogel primarily composed of water and held together by MUC‐2 mucin proteins, which acts as both lubrication for luminal material and a physical barrier between the epithelial cells and this material. Mucus exists as a single layer in the small intestine and contains antimicrobial mediators.^[^ ^12^ ^]^ In the colon, the adherent inner layer of mucus (unstirred) functions similarly to the single layer in the small intestine, while the looser outer layer (stirred) acts as a protective biofilm with the presence of bacteria.^[^ ^13^ ^]^

Also present in the epithelium are enteroendocrine cells (EECs), that sense metabolites, products of digestion, and signals from the microbiome, such as fatty acids, amino acids, and lipopolysaccharides.^[^ ^14^ ^]^ In response, EECs release various hormones like motilin, cholecystokinin, and peptide YY, which modulate motility, digestion, and satiety, respectively.^[^ ^15^, ^16^, ^17^ ^]^ Additionally, stems cells situated at the base of intestinal crypts differentiate into different epithelial cell types, providing a system for rapid self‐renewal and repair. These stem cells differentiate into one daughter cell and one transit amplifying (TA) cell. These TA cells migrate upwards along the edge of the crypt for two to three days, and upon reaching the crypt villus junction become terminally differentiated.^[^ ^18^ ^]^ All cells then migrate and divide upwards toward the tip of the villi, aside from Paneth cells, which migrate downwards.^[^ ^19^ ^]^ The balance between the apoptosis of epithelial cells at the tip of the villi and the production of TA cells is of upmost importance to the maintenance of a healthy epithelial barrier. An imbalance can lead to cancerous growth and inflammation^[^ ^20^ ^]^ and can be caused by changes to the Notch^[^ ^21^ ^]^ and Wnt^[^ ^22^ ^]^ signaling pathways.

### 2.2 Immune System and the Gut

**Figure 3:**
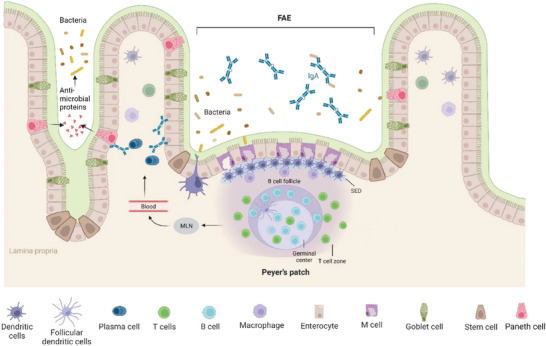
Outline of the immune response in the intestine upon bacterial infection. Adapted with permission.^[^ ^48^ ^]^ Copyright 2014, Taylor and Francis. Paneth cells perform primary immune responses, and release antimicrobial peptides upon activation by bacteria. The gut‐associated lymphoid tissue includes Peyer's patches, which lie under the M cell rich follicle‐associated endothelium (FAE). The subepithelial dome (SED) is abundant in dendritic cells and macrophages. B cells reside within follicles and T cells within interfollicular spaces. T helper cells activate B cells via the mesenteric lymph nodes (MLNs). Activated B cells differentiate into plasma cells in the Germinal center and secrete antigen specific IgA into the lumen.

The second of the main functions of the GI system is the mediation of the interaction between the immune system and the gut microbiome. The microbiome consists of many bacteria that help metabolize nutrients and strengthen the immune system.^[^ ^41^ ^]^ Numerous comprehensive reviews explore the intricate role of the complex and far‐reaching microbiome in maintaining gut homeostasis.^[^ ^42^, ^43^ ^]^ This review aims to concentrate on the dynamic interplay between the microbiome and the immune system and the models which emulate this. **Figure** **3** displays an outline of the different primary and lymphoid tissue‐based responses in the gut.^[^ ^44^ ^]^

Primary immune defenses within the intestine exist in the epithelial layer. Previously mentioned enterocytes, while providing a physical barrier, can also directly expose antigens to regulatory T cells through antigen presenting molecules such as MHC class II.^[^ ^45^ ^]^ The main primary immune cell in the epithelial later is the Paneth cell, which are granule loaded cells located within the crypts in the small intestine. Pathogenetic stimulation via antigens or cholinergic mediators causes release of the cell's granules^[^ ^46^ ^]^ which contain antimicrobial peptides and growth factors, such as amyloids and lipokines.^[^ ^47^ ^]^ In addition, the mucus produced by goblet cells acts as a non‐specific defense mechanism, housing the released antimicrobial peptides and forming a mesh‐like network acting as a steric barrier.^[^ ^48^ ^]^

The gut‐associated lymphoid tissue (GALT) encompasses tissues in the gut that are involved with initiating and priming immune responses, such as the Peyer's patches (PP), colonic patches, and the isolated lymphoid follicles (ILF).^[^ ^49^ ^]^ Although similar in structure, PPs are located purely within the small intestine, whereas ILFs can be found in the colon too.^[^ ^50^ ^]^ PPs are formed of a subepithelial dome (SED) which is rich with dendritic cells (DCs) and macrophages. There are follicles rich with B cells, containing germinal centers (GCs). T‐cells are primarily found within the inter‐follicular regions.^[^ ^51^ ^]^ The SED is underneath a specialized area of epithelium layer (follicle‐associated epithelium (FAE)), within the lamina propria and submucosa. The FAE is characterized by low numbers of microvilli, a thin mucus, and an abundance of microfold (M) cells.^[^ ^51^ ^]^

M cells manage the transcytosis of microbial antigens from the lumen to the lymphoid tissue, thereby initiating the immune response.^[^ ^52^ ^]^ Upon receipt of antigens, DCs and macrophages activate helper T cells, which pass via the bloodstream in the highly vascular lamina propria to the immune response amplifying mesenteric lymph nodes.^[^ ^40^ ^]^ In addition, macrophages secrete anti‐inflammatory mediators, cytokines and start phagocytosis of bacteria.^[^ ^35^ ^]^ Activated B cells gather in the GCs of the PP and differentiate into specialized plasma cells. The cumulation of the immune response is the secretion of antigen‐specific Immunoglobulin A (IgA) by these plasma cells.^[^ ^53^ ^]^ Memory B cells are also produced in the GCs and provide prolonged intestinal protection.^[^ ^54^ ^]^ The immune response within the intestine is vital to maintaining the homeostasis of the microbiome, the GALT is ≈70% by weight of the entire immune system.^[^ ^55^ ^]^

In addition to the crucial role played by immune cells in inflammatory processes, the peripheral nervous system also assumes significance. The intestinal epithelium is innervated by specialized damage detecting sensory neurons called nociceptors, whose cell bodies are in the dorsal root ganglion. When exposed to bacteria, these nociceptors are triggered by N‐formyl peptides.^[^ ^56^ ^]^ This activation sets off a subsequent neurogenic immune response by inducing the release of specific neuropeptides, most notably calcitonin gene‐related peptide (CGRP).^[^ ^57^ ^]^ These neuropeptides, in turn, lead to vasodilation^[^ ^58^ ^]^ and the activation of immune cells, including mast cells.^[^ ^59^ ^]^

## 3 Investigating Intestinal Pathogens

Host‐pathogen interactions vary largely between pathogens in terms of the intestinal region in which they interact with. Indeed, many pathogens can be considered opportunistic in that they do not typical cause disease in healthy individuals. When the host's defenses are lowered, often due to the presence of disease or as a side effect of medication,^[^ ^60^ ^]^ some commensal bacteria can become virulent. This review will not make the distinction between conventional pathogens and opportunistic pathogens.

### 3.1 Common Intestinal Pathogens

A selection of common intestinal pathogens is presented in **Table** **2** along with the intestinal location that they infect and the current understanding of the invasion or host entry mechanism that is found in literature. While being far from exhaustive, this list demonstrates the breadth of invasion mechanisms and demonstrates the necessity for in vitro models capable of modelling the mucus layer, cell membrane and the native epithelium.

**Table 2:** Common pathogens which cause intestinal infections.

Pathogen	Clinical Symptoms	Intestinal location infected	Invasion or Entry mechanism	Ref
Bacteria				
*Vibrio cholera*	Watery diarrhoea, vomiting, thirst, leg cramps, restlessness	Small intestine	Motility and mucinases allow penetration of mucus layer. GbpA assists binding to epithelial cells. The endotoxin, cholera toxin, binds to GM1 ganglioside and is internalised. Leads to increase in cAMP.	[61, 62, 63, 64]
*Campylobacter jejuni*	Diarrhoea (often bloody), fever, stomach cramps, nausea, vomiting	Distal ileum, jejunum, cecum, and colon. Particularly within crypts.	Flagella promote motility, chemotaxis, and colonisation. Flagella facilitates secretion of *campylobacter* invasion antigens (Cia), including CiaC and CiaI. Adhesins promote attachment including to basolateral fibronectin which precedes secondary invasion mechanisms. Potential role for membrane lipid, lysophosphatidylethanolamine, in permeabilising host cell membrane.	[65, 66, 67, 68]
*Clostridium* difficile	Diarrhoea, fever, stomach pain, loss of appetite, nausea	Reproduce in the small intestine but infect the colon.	Based on the action of two toxins, A and B coded by TcdA and TcdB. These may disrupt the host cytoskeleton and tight junctions leading to cell detachment and death.	[69, 70, 71]
*Shigella dysenteriae*	Prolonged or bloody diarrhoea, fever, stomach pain, feeling the need to defecate when bowels are empty	Colon. Gut‐associated lymphoid tissue (GALT).	*Shigella* traverses the epithelium through M‐cells. Phagocytosed by antigen‐presenting cells, lyses the phagosome, induces lysis of cell membrane. Type III secretion system (T3SS) contacts the basolateral host plasma membrane and facilitates injection of effectors causing uptake into epithelial cells. *Shigella* replicates in the cytosol and spreads to neighbouring cells.	[72, 73]
*Listeria monocytogenes*	Fever, muscle ache, headache, convulsions, diarrhoea	Colon	Receptor‐mediated uptake in epithelial cells by binding of internalin A and B to the host receptors E‐cadherin and Met, or *Listeria* adhesion protein (LAP) interaction with HSP‐60. In goblet cells, *L. monocytogenes* can traverse across the cell and transcytose. There are also interactions observed with M‐cells.	[74, 75, 76, 77]
*Salmonella enterica* Serovar Typhimurium	Diarrhoea (can be bloody), fever, stomach cramps, nausea, vomiting, headache	Colonises ileum and affects small and large intestine	T3SS allow *Salmonella* to inject effectors across the epithelial cell membrane causing membrane ruffling and internalisation of the bacterium. T3SS also used to alter host cell vacuoles and limit effect of lysosomes to prevent enzymatic degradation. Allows bacterium to survive and replicate in host cytoplasm.	[78, 79, 80]
*Escherichia coli* (EHEC and EPEC strains)	Stomach cramps, diarrhoea, vomiting, low grade fever, (EHEC: haemorrhagic colitis and hemolytic uremic syndrome)	EHEC: mainly Peyer's patches and colon EPEC: Duodenum, terminal ileum, and Peyer's patches.	Tight attachment to epithelium formed during colonisation. Formation of attaching and effacing A/E lesions characterised by damage to microvilli and formation of dense actin plaques below. Uses T3SS to deliver effector proteins. EHEC delivers Shiga toxin, Stx, which inhibits protein synthesis and induces apoptosis.	[81, 82, 83, 84]
Viral				
Norovirus	Diarrhoea, vomiting, nausea, stomach pain		Noroviruses bind to histo‐blood group antigens (HBGAs) which are glycan attachment factors on host cells including enterocytes. Entry and viral uncoating then occur with positive‐sense RNA translated in the cytoplasm. Shown to infect organoids and IECs. M‐cells may be used to deliver NoV to lymphocytes, macrophages and DCs which are also susceptible.	[85, 86, 87, 88, 89]
Rotavirus	Vomiting and watery diarrhoea	Upper two‐thirds of the small intestine	Binding of viral capsid protein VP4 to host cell receptors including sialoglycans (GM1 and GD1a) as well as HBGAs, sialic acid and Hsc70. Proteases in the GI tract cleave VP4 spike into VP8 and VP5 which facilitate viral penetration. Virus particles are internalised by receptor‐mediated endocytosis.	[90, 91, 92]
Adenovirus	Flu‐like symptoms, fever, sore throat, acute bronchitis, pneumonia, conjunctivitis, acute gastroenteritis	Small and large intestine. Latent infection in Peyer's patches.	HAdV‐2 and 5 attach to the surface of cells via the coxsackievirus and adenovirus receptor (CAR). Other types attach to CD46, GD1a and desmoglein‐2. Entry is facilitated by interactions between virus penton base protein and α5β3 and α5β5 integrins. Phosphatidylinositol 3‐OH kinase is necessary for internalisation. Post‐internalisation viruses disrupt the early endosome, are transported to the nucleus and capsid uncoating reveals viral DNA.	[93, 94]
Astrovirus	Mild, watery diarrhoea, nausea, vomiting, abdominal pain, fever	Mainly effecting the epithelium of the small intestine	Astroviruses typically rely on trypsin to cleave the capsid precursor. Human astrovirus VA1 was differs in that it can be cleaved intracellularly to yield mature infectious particles. The receptors for human astroviruses are yet to be determined. Capsid proteins can affect the regulation of tight junctions which may be a pre‐requisite for accessing its receptors.	[95, 96, 97, 98, 99, 100, 101, 102]
Coronavirus (SARS‐CoV‐2)	Respiratory symptoms, loss of smell and taste, diarrhoea, nausea, vomiting	Small and large intestine	Angiotensin‐converting enzyme 2 (ACE2) receptors are fundamental to cellular entry of SARS‐CoV‐2. ACE‐2 receptors are abundant in the intestine – even more so than in the lungs. The viral spike protein is primed by TMPRSS2 which facilitates invasion.	[103, 104, 105, 106, 107]
Fungi				
*Candida albicans*	Pain when swallowing, white matches in mouth, loss of taste, stomach pain, cramping, weight loss, diminished appetite	Colonisation of small and large intestine	Yeast cells adhere to the host via adhesins. Contact causes yeast‐to‐hypha transition which is known to be associated with host cell invasion. Invasin (Als3 and Ssa1) expression mediated binding to epithelial cells e.g., E‐cadherin, and induces endocytosis into host cell. Virulence factors are expressed including the pore forming toxin candidalysin.	[108, 109, 110, 111]

## 4 In Vitro Models

In vitro models are fundamental to modelling the structure of the native intestine due to the lack of complexity, heterogeneity, and low throughput of in vivo models. The precise control over experimental conditions allows for the deconvolution of contributing factors to disease in a reductionist approach. Complexity can be added where necessary to answer biological questions for example by the inclusion of multiple cell types, engineering of native architectures, or by the mimicking of the in vivo conditions including pH, oxygen levels, and mechanical forces. The cellular complexity in models studying host‐pathogen interactions should align with the specific biological question. Cell lines are suitable for exploring the specific details regarding pathogen's interaction mechanisms. Conversely, organ or body‐on‐chip models, which are discussed in detail in other reviews,^[^ ^112^, ^113^ ^]^ may be better suited for assessing the potential for pathogen interaction, invasion, and colonization when crosstalk between cell types and organ systems is concerned. However, unnecessary complexity could obscure the true host‐pathogen interaction mechanism if the added intricacies of such models are not pertinent. More intricate models, which replicate native biology, can be developed by introducing numerous cell types that mimic the breadth of specialized cells found in vivo. More physiologically relevant models often employ primary cells and organoids models extracted from patient biopsies.

When selecting cell types for exploring host‐pathogen interactions, it is often desirable to start with established cellular models and gradually introduce complexity by adding additional cell types. Furthermore, when confronted with an unknown disease‐causing organism in which the manifestation of that disease is unclear, a model which has a broader scope should be used. Ideally, the model would include a variety of tissue/cell types to ascertain where and how the pathogen interactions with the system. With regards to the intestine, as a first‐pass approach, it would be useful to select a well‐understood and accepted model which is likely to initially include well‐established cell lines. To comprehensively capture the intricate crosstalk between cell types and mimic the human body's microenvironment, this strategy culminates in the utilization of patient‐derived organoids. These organoids provide a more precise representation of disease heterogeneity, enhancing our understanding of complex interactions. Indeed, in our view, patient biopsy‐derived cells, including organoids or dissociated organoids, will be the future of in vitro models replicating the in vivo physiology of the gut. This is largely due to the presence of all relevant cell types which cannot be faithfully replicated with cell lines. As such, cell line‐only models cannot faithfully replicate the structure or function of the in vivo intestine – this does, however, not mean they are not incredibly useful tools for probing well‐defined biological questions. The features of such cells and their applications are considered in this section and a summary of in vitro models for monitoring intestinal host‐pathogen interactions is provided in **Table** **3**.

**Table 3:** In vitro models for monitoring intestinal host‐pathogen interactions.

Model	Advantages	Disadvantages	Examples		
			Pathogen	Host Cell(s)	Ref
Planar Monoculture 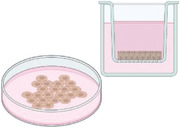	‐ Easy to culture ‐ Relatively cheap ‐ Cell culture inserts allow for basolateral access ‐ Can be adapted for a quasi‐3D system	‐ One cell type only ‐ Mostly 2D ‐ Cell lines are normally cancerous ‐ Long culture times of cell lines (≈3 weeks)	*S. typhimurium*	Caco‐2	[114]
			*C. jejuni*	Caco‐2, INT 407	[115]
			*Rotavirus*	HT‐29	[116]
Planar Co‐culture 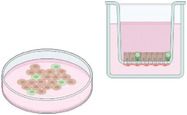	‐ Easy to culture ‐ Relatively cheap ‐ Added complexity of multiple cell types ‐ Cell culture inserts allow for basolateral access ‐ Can be adapted for a quasi‐3D system	‐ Cell lines are normally cancerous ‐ Mostly 2D ‐ Long culture times of cell lines (≈3 weeks) ‐ ‐May require optimized media conditions	‐	Caco‐2, HT29‐MTX, Raji B, Cord blood mononuclear cells	[117]
			Adenovirus	Caco‐2, Raji B, mouse lymphocytes	[118]
Scaffold 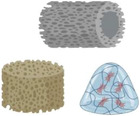	‐ Substrates designed to match physical properties of ECM ‐ Houses multiple cell types ‐ 3D morphology	‐ Understanding of mechanical and chemical properties required ‐ Thicker scaffolds can be difficult to image and monitor	*E. coli LF82*	Caco‐2, HT29‐MTX	[119]
			Stool sample including *E. coli, Shigella, Streptococcus*	Human jejunum intestinal enteroids	[120]
Organoids 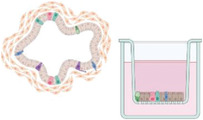	‐ ex vivo ‐ 3D structure ‐ Can be dissociated into monolayers ‐ Contains all cell types found in vivo	‐ Expensive to culture ‐ High level of skill needed ‐ Long culture periods ‐ Difficult to access apical side without disruption	*C. difficile*	Human intestinal organoids	[121]
			SARS‐CoV and SARS‐CoV‐2	Human small intestinal organoids	[122]
Tissue Samples 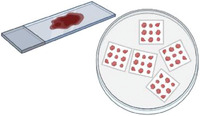	‐ Differentiated tissue ‐ Contains all cell types found in vivo ‐ 3D morphology ‐ No culture time	‐ Supply is limited ‐ High level of skill required to culture and image ‐ Limited time frame (≈<24 hours)	‐	Human precision cut intestinal slices	[123]
Gut‐on‐a‐chip 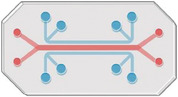	‐ Often incorporates fluid flow ‐ Can host multiple cell types ‐ Often allow for real time/live monitoring	‐ Expert skill required to manufacture the chips ‐ Limited chip availability ‐ Capable of continuous treatment with microorganisms under flow to prevent overgrowth	*Cryptosporidiun parvum* oocysts	Dissociated mouse intestinal organoids	[124]
			*Salmonella typhimurium*	Dissociated mouse colon organoids	[125]
Planar lipid membranes 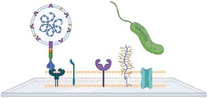	‐ Simplistic model ‐ Can be interfaced with electronics for quantitative readout ‐ No continuous cell culture	‐ No active cellular processes ‐ Expert skill required for formation and manipulation ‐ Super resolution microscopy needed to adequately image	*Staphylococcus aureus, Pseudomonas aeruginosa* and secreted toxins	Synthetics lipids including 2,3‐di‐O‐phytanyl‐glycerol‐1‐tetraethylene glycol‐d,l‐lipoic acid ester lipid (DPhyTL) and cholesterol‐pentaethyleneglycol (CholPEG)	[126]
Mucus models 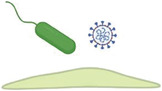	‐ Can be from ex vivo, in vitro, synthetic, or semi‐synthetic ‐ No live cell component ‐ Tuneable properties ‐ Can be used to probe pathogen interactions	‐ Variability in commercially available mucin compositions ‐ ex vivo mucus difficult to obtain	*Candida albicans*	Native porcine gastric (MUC5AC) and intestinal (MUC2) mucus	[127]
			*Vibrio cholerae*	Native porcine gastric (MUC5AC) and intestinal (MUC2) mucus	[128]

### 4.1 Cell Types

Models typically use a range of cell types which together mimic the complex cellular composition, structure, and function of the intestinal epithelium. The primary cell type constituting the epithelial wall are intestinal epithelial cells (IECs) which include enterocytes, goblet cells, enteroendocrine cells, and Paneth cells. Stem cells are fewer in number but are responsible for the renewal of the intestinal epithelium through the differentiation into a range of epithelial cell types and therefore long‐term maintenance. Contributing to the regulation of the homeostasis of the microbial microenvironment are immune cells which are often included for immune surveillance and investigation of host‐pathogen interactions. An oversight in many intestinal models is their absence of the microbiome which has a wide‐reaching impact on the structure and function of the intestine and is therefore necessary to fully recapitulate the native environment in vitro. Of course, the relevance of the cellular composition of any model is entirely dependent on the biological question under investigation.

#### 4.1.1 Cell Lines

Immortalized cell lines remain the work horse of in vitro intestinal models due to their relative ease of culture, low‐cost, and potential for scale up. A summary of the common cell lines and their characteristics are summarized in **Table** **4**. Two of the most common cell lines used to date are Caco‐2 and HT29. The Caco‐2 human colorectal adenocarcinoma cell line has long been accepted as the standard model for mimicking the epithelium's transport and permeability functions. Derived from a 72‐year‐old white male, this line displays a non‐homogenous morphology. Upon reaching confluency the cells begin to express the phenotype of mature enterocytes; including polarized apical brush border and basolateral regions delineated by the localization of tight junctions. While Caco‐2 form tight barriers through which reproducible permeability studies and trans‐epithelial resistance measurements are observed, they lack the functionality of the in vivo epithelium's mucus layer and hormone secretion. Furthermore, the inherent heterogeneity in the population of Caco‐2 cells leads to cellular models with properties that differ from the original cell line because cultivation conditions can select for growth of a particular phenotype.^[^ ^129^ ^]^ The culture time and passage number of the cell line has also been shown to significantly influence the TEER, 14C‐mannitol permeability, sucrase activity, and alkaline phosphatase activity in addition to the formation of multicellular layers.^[^ ^130^ ^]^ The typical 21‐day differentiation time can impose limitations on throughput, though groups have adapted culture conditions – both media composition and the introduction of shear stress – to accelerate differentiation^[^ ^131^, ^132^ ^]^

**Table 4:** A summary of cell lines commonly used for in vitro models of the intestinal epithelium.

Cell line	Characteristics	Origin	Application	Ref
Caco‐2	Cylindrical polarised monolayer, apical brush border with enzyme secreting microvilli, tight junctions between adjacent cells, forms domes	Human colon adenocarcinoma	Barrier models, permeability assays, drug absorption, metabolism, 3D cell culture, toxicology	[144, 145, 146]
HT‐29	Cylindrical polarised monolayer, apical brush border with enzyme secreting microvilli, tight junctions between adjacent cells HT29‐MTX are mucin producing and goblet cell‐like	Human colon adenocarcinoma	Barrier models, drug absorption, metabolism, cancer research, 3D cell culture, toxicology, mucus extraction	[147, 148, 149]
T84	Like Caco‐2 but with more colonocyte‐like features including shorter microvilli. Generally, exhibit higher TEER than Caco‐2.	Human colon carcinoma (lung metastasis)	Cancer research, neurotransmitter and hormone studies, barrier models	[150, 151, 152]
SW480	Heterogenous cell population producing polygonal cells of the typical epithelial type (E‐type) and round refractile cells (R‐type). R‐type forms multilayers. Large microvilli and glycogen stores. Deep processes observed through inserts	Human colon adenocarcinoma	Cancer research, 3D cell culture, drug delivery, migration studies	[153, 154, 155]
IEC‐6	Normal cell type. Synthesise fibronectin and collagen. Tight colonies with polygonal shape. Crypt cell characteristics and tight junctions.	Rat small intestine epithelia	3D cell culture, transport studies, ECM, parasites, healthy cell metabolism	[156, 157, 158]
HCT‐8	Heterogenous organisation and small proportion of cells expressing SI, villin and ZO1. Methotrexate‐selected HCT8‐MTX cells show universal expression of ZO1 and MUC1.	Human ileocecal adenocarcinoma	3D cell culture, cancer research, toxicology, virus entry, parasite,	[159, 160, 161, 162]
FHC	Normal cell type. Exhibits tumorigenic phenotype.	Foetal colonic epithelial	3D cell culture, drug delivery, cancer research, foetal research, uptake	[163, 164]
Raji	Grow as single cells without attachment. Clumps may form. Carries latent Epstein‐Barr Virus.	Human B lymphoblastoid	Toxicity, 3D cell culture, immunology	[139, 140, 165, 166]
THP‐1	Grown in suspension. Can be stimulated by phorbol‐12‐myristate‐13‐acetate (PMA) into macrophage‐like cells	Human monocytic leukaemia	3D cell culture, immunology, toxicology	[167, 168, 169]

A significant characteristic of the epithelium is a mucus layer. The HT29 cell line is a well‐characterized model for studying intestinal host‐pathogen interactions due to the interaction of some pathogens with the mucin secreted by these cells, and the steric barrier that the secreted mucus provides. Derived from the primary tumor of a 44‐year‐old Caucasian female, this line has since been treated with methotrexate (MTX) to isolate a differentiated mucus‐secreting phenotype.^[^ ^133^ ^]^ Structurally, differentiated HT29 cells express a phenotype like small intestinal enterocytes with the presence of brush border hydrolases and similar villin expression to fresh colonocytes. However, the usefulness of these cells as an intestinal model may be impacted due to the high rates of glucose metabolism (cancerous cells) and lack of expression of typical hydrolases including lactase and maltase‐glucoamylase.^[^ ^134^ ^]^ Typical models of the intestinal epithelium are composed of both HT29‐MTX and Caco‐2 cells seeded in a 1:9‐3:7 ratio to mimic in vivo conditions.

While Caco‐2 and HT29 are commonplace, they are no means the only cell lines used in vitro intestine models. The T84 epithelial cell line is derived from the lung metastasis of human colon carcinoma and displays similar characteristics as Caco‐2 cells and as such are often used interchangeably. It has since been shown that there are functional, biochemical, and structural distinctions between these cell types as T84 retain much of their colonic characteristics including shorter microvilli and a dose‐dependent increase in barrier integrity in response to butyrate.^[^ ^135^ ^]^

The immune component is surprisingly often omitted in cell culture models – even where the focus is the investigation of host‐pathogen interactions. M‐cells found in Peyer's patches are major sites of microorganism and antigen sampling.^[^ ^136^ ^]^ Mature M‐cells are scarce in vivo, but methods have been developed to convert enterocyte‐like cells into cells that express both functional and morphological features of M‐cells. Caco‐2 cells seeded basally on cell culture inserts and differentiated into a mature phenotype exhibit M‐cell characteristics when incubated with primary Peyer's patch lymphocytes or the Raji lymphoblast‐like cell line.^[^ ^137^, ^138^, ^139^, ^140^ ^]^ The human monocyte line THP‐1 and their mature macrophage or dendritic lineage has also been incorporated into IEC models to investigate host‐pathogen interactions and resulting inflammation.^[^ ^141^, ^142^, ^143^ ^]^

Cell lines are not able to truly mimic all of the subsets of epithelial cells found in vivo, however, the application may not entirely necessitate a physiologically complete model. When trying to elucidate fundamental mechanisms it is often useful to begin with a well characterized and understood system in a reductionist manner and later build up complexity.

#### 4.1.2 Primary Cells and Organoids

The in vivo epithelium is a highly organized architecture built by the continuous maturation of multipotent stem cells. Asymmetric mitosis leads to a population of cells that are rapidly renewed as they migrate along the crypt‐villus axis. Differentiated cells migrate, die, exfoliate into the intestinal lumen, and are subsequently replaced.^[^ ^170^ ^]^ Primary culture refers to cells that have been extracted from tissue and cultured in vitro without any passaging. The integrity of the epithelium in vivo is maintained by cell‐to‐cell or cell‐to‐extracellular matrix (ECM) contacts. A breakdown of such interactions can initiate apoptosis – termed anoikis.^[^ ^171^ ^]^ Intestinal stem cells are particularly susceptible to anoikis which limits in vitro culture. This has long hindered their successful culture. Primary cells cultured in monolayers also suffer due to a lack of knowledge of optimum media compositions and the incompatible physical and chemical properties of supporting substrates.^[^ ^172^ ^]^ Advances were realized once primary epithelial cells were cultured on ECM‐coated porous membranes which vastly improved the culture of differentiated and non‐proliferative epithelial monolayers across a range of species.^[^ ^173^, ^174^, ^175^ ^]^

While primary intestinal epithelial cells (IECs) traditionally exhibit a low survival rate ex vivo, organoids derived from intestinal crypt stem cells overcome this limitation by demonstrating enhanced survival and proliferation ex vivo. First isolated from mouse small intestinal tissue,^[^ ^176^ ^]^ intestinal organoids are derived from crypt‐resident Lgr5+ adult stem cells and can be derived from adult primary tissue, as well as IPSCs and embryonic stem cells. Organoids are nevertheless limited by the Hayflick limit^[^ ^177^ ^]^ and thus cannot be propagated indefinitely as is the case for immortalized cell lines. Lgr5+ crypt cells display directional polarization and the capacity to develop into various cell types, while concurrently developing villus and crypt‐like domains which are correctly compartmentalized into the lumen and mucosal sides of the organoids. They show the capacity to self‐organize into 3D structures, displaying highly in vivo‐like morphologies^[^ ^178^ ^]^ as seen from histological images,^[^ ^179^ ^]^ absorptive functionality,^[^ ^180^ ^]^ transporter activity,^[^ ^181^ ^]^ and their ability to differentiate into pertinent intestinal cell types^[^ ^182^ ^]^ including correctly polarized Paneth, goblet, enteroendocrine cells, and enterocytes.^[^ ^176^ ^]^ Once isolated and grown in a 3D environment providing ECM‐interactions such as hydrogels, Lgr5+ stem cells can proliferate and form the classical 3D structure of organoids.

Intestinal organoids may be derived from across the small^[^ ^183^, ^184^ ^]^ and large intestine,^[^ ^185^ ^]^ and maintain their respective GI region‐specific expression profiles,^[^ ^184^ ^]^ thus providing a representative experimental platform. This further enables matching of organoids to gut segment‐specific pathogens, such as colonic and small intestine‐derived organoids for rotavirus,^[^ ^186^, ^187^ ^]^ norovirus,^[^ ^86^, ^89^ ^]^ *C. difficile* and *Shigella*.^[^ ^188^ ^]^

The cell surface of gut epithelial cells can be categorized into the apical (luminal) and basolateral (mucosal) regions, which serve distinct roles and display distinct expression profiles. This polarization along the apical‐basal axis is crucial for proper epithelial function and is recapitulated in organoids which exhibit apical tropism toward the lumen.^[^ ^176^ ^]^ IEC apical/basal domains possess specialized expression of transporters, enzymes, receptors, and adhesion and signaling molecules, of which the distinct segregation of membrane proteins is essential for maintaining the functional integrity of the intestinal epithelium, as it allows for the establishment of polarized transport processes and selective permeability. This established cell polarity is a critical aspect of gut physiology and plays a fundamental role in the proper functioning of the digestive system and is regulated by various intracellular trafficking pathways.^[^ ^189^ ^]^

It is thus of paramount importance for disease models to faithfully recreate this apical‐basal compartmentalization, especially in context of diseases which are affected by disruptions in apical‐basal cell polarity and gut barrier function such as inflammatory bowel disease^[^ ^190^ ^]^ – for example, in which Crohn's disease patients have been shown to display disrupted expression of key immunomodulatory proteins including cytokine receptors,^[^ ^191^ ^]^ TLRs,^[^ ^191^ ^]^ as well as protein polarity complexes.^[^ ^192^, ^193^ ^]^

#### 4.1.3 Tissues

One of the limitations to the use of organoids, and cell lines, for in vitro models is their long culture time, which hinders high‐throughput experimentation; alternatively, explanted tissue segments can be used upon culturing. Additionally, their benefits encompass a cellular environment that closely mimics in vivo while facilitating controlled experimentation. Intestinal tissues are routinely extracted from the small intestine and of rats, mice, and humans.^[^ ^194^ ^]^ Explanted tissue‐based research has not only focused on host‐pathogen interactions^[^ ^195^, ^196^ ^]^ but also drug efficacy and toxicity studies^[^ ^197^, ^198^ ^]^ and immune response investigations.^[^ ^199^ ^]^

**Figure 4:**
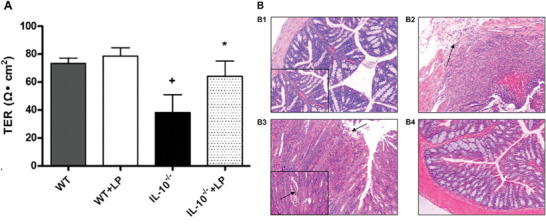
The action of probiotic Lactobacillus plantarum (LP) on interleukin‐10 knockout (IL‐10−/−) mice compared to wild type (WT) mice. A) TER measurements from of colonic mucosae in an Ussing chamber, with *n* = 6 and +*P* < 0.001 compared with WT mice, *P < 0.05 compared with IL‐10−/− mice. B) Histology shows healthy tissue in B1 (WT), effects of colitis, necrosed tissue and presence of lymphocytes and neutrophils in B2 and B3 (IL‐10‐/‐) and recovering tissue in B4 (IL‐10−/−+LP). Adapted with permission.^[^ ^195^ ^]^ Copyright 2010, American Physiological Society.

Two primary approaches exist for conducting tissue‐based ex vivo studies: intact tissues and sliced segments. Intact tissues are extracted by either by cutting them out or using a biopsy tool for punching. The full thickness of a rat colon measures over 0.5 mm^[^ ^200^ ^]^; maintaining the viability of such thick and vascularized tissue outside of the body is a significant challenge, as the oxygen diffusion to the tissue's center is limited. The viability of intact tissues is typically ≈4 h in ex vivo conditions.^[^ ^201^ ^]^ Intact tissues are often used within an Ussing chamber, a technique described in more detail further on. Ussing chambers have been used in studies of bacteria on small intestines^[^ ^202^ ^]^ and colons of mice,^[^ ^195^, ^203^ ^]^ rat, and rabbits.^[^ ^202^ ^]^ The impact adding whole probiotic bacteria such as *Lactobacillus plantarum* ^[^ ^195^ ^]^ and *Lactobacillus acidophilus* ^[^ ^203^ ^]^ as well as toxins produced by harmful bacteria such as *Shigella dysenteriae* ^[^ ^202^ ^]^ have been studied. The Ussing chamber was used to monitor changes in permeability upon the addition of each of these bacteria. The combination of the Ussing chamber and histology, as shown in **Figure** **4**, in addition to western blotting and immunofluorescence was used to confirm *Lactobacillus plantarum*’s efficacy in reducing the intestinal permeability of interleukin‐10 knockout mice through the regulation of the apical junction complex.^[^ ^195^ ^]^ Besides testing bacteria on intact tissue, it is also feasible to reverse the process and test intact tissue products on bacteria. The antimicrobial peptides produced by colon tissue cultured in a 6 well plate on response to IL‐1β and IL‐18 were used to successfully kill cultured *Escherichia coli*.^[^ ^196^ ^]^

Sliced segments are obtained through a technique called precision cut intestinal slices (PCIS) in which slices of the intestine are either filled with agarose and sliced perpendicularly to the lumen or stripped off muscle and sliced through the layers.^[^ ^204^ ^]^ The invention of the Krumdieck tissue slicer in 1980^[^ ^205^ ^]^ facilitated reproducible production of tissue slices between 0.1 to 0.4 mm thick. The viability of sliced rat colonic tissue, measured using intracellular ATP, can be up to 24 h when the tissue is under constant oxygenation.^[^ ^201^ ^]^ The viability depends on both the animal the tissue was taken from, and the location within the gastrointestinal tract, with small intestines having a shorter viability than colons. Microfluidic channels can be utilized to increase the viability of mice colon explants to 192 h.^[^ ^206^ ^]^ Other advantages of PCIS include reproducibility with 100 s of slices able to be obtained from one section of intestine and high throughput, with these slices able to be handled within a few hours.^[^ ^207^ ^]^ Regulating the thickness also means slices can be compared across species.^[^ ^208^ ^]^ Their application to intestinal host‐pathogen interactions has been relatively unexplored, because due to the perpendicular nature of the slices, transport studies across the epithelium are unable to be obtained with this method. However, intestinal slices from chicken embryos, with a viability of 4 days, infected by avian influenza virus were immune‐stained and it was possible to confirm the presence of viral antigens in epithelial cells.^[^ ^209^ ^]^

The advantage of both techniques over othe*r* in vitro models is the preservation of aspects of the in vivo environment and increase in physiological relevance. Slices and biopsies have been shown to have a mucus layer,^[^ ^210^ ^]^ complete epithelial polarization,^[^ ^211^ ^]^ the inclusion of a full enteric nervous system with the presence of the interstitial cells of Cajal, Peyer's patches, crypts, and villi.^[^ ^199^ ^]^ However, with advanced in tissue engineering, many of these in vivo features can now be emulated in vitro.

### 4.2 Conventional Models

The term ‘conventional’ refers to existing cell culture models which do not incorporate any additional design, engineering or sensing principles beyond that of standard cell cultureware. Such models may be compatible with external equipment, for example cell culture inserts can be coupled with electrodes to measure transepithelial electrical resistance (TEER) of cell monolayers, but they themselves do not have additional sensing functionalities.

#### 4.2.1 2D Planar Models

2D models are long established and commonplace in cell culture. They consist of the culturing of a mono‐, co‐, or multi‐culture of cell types on a flat surface, typically a plastic flask or dish. Such plastic substrates can be coated with ECM proteins to aid adherence. Media is added to cover the cell layer allowing for the delivery of essential nutrients. Such a system is far removed from the native environment of the intestinal epithelium. The hard, flat plastic is not conducive to the formation of structures which simulate the intestine, and do not allow for access to the basolateral side of the cell thus limiting applications. Furthermore, gene expression and protein expression are altered resulting in a loss of in vivo functionality.^[^ ^212^ ^]^

The development of the cell culture insert system – a simple thin, porous, membrane, has since addressed one limitation of 2D culture by allowing for distinct reservoirs apically and basolaterally for the supply of nutrients, removal of waste products and for the application of treatment conditions. Typical uses of the cell culture insert models are for the high throughput investigation of barrier integrity and of uptake and mass transfer of molecules of interest.^[^ ^213^ ^]^ The inserts allow for the facile integration with electrical equipment to monitor the transepithelial electrical resistance TEER of the monolayers on the porous membrane.

Regarding organoids, conventional host‐pathogen interaction studies have been shifting toward 2D cultured monolayer systems over self‐assembling organoids due to the difficulty in luminal access. Organoids are dissociated, mechanically^[^ ^214^ ^]^ or enzymatically,^[^ ^214^ ^]^ and seeded onto membrane inserts as monolayers whereupon the stem cells can be induced to differentiate into pertinent cell types. Organoid‐derived monolayers have been shown to maintain consistent gene expression with in vivo tissue, as well as functional characteristics (fluid/ion absorption and secretion^[^ ^175^ ^]^), and maintenance of different gut cell types such as goblet cells, which have been shown to display mucus secretion in cell culture insert systems.^[^ ^215^ ^]^ 2D platforms enable compartmentalization of apical and basal compartments, control of morphogenic gradients, and apical/basal‐specific control and measurements. Organoid‐derived monolayers additionally promotes IEC differentiation.^[^ ^175^ ^]^ Like 3D organoids, 2D organoid‐derived monolayers can be paired with co‐culture systems or a range of different cell types under in vivo‐like conditions. This includes luminal bacteria, endothelial,^[^ ^216^, ^217^ ^]^ immune^[^ ^218^ ^]^ neural,^[^ ^219^ ^]^ and mesenchymal cells,^[^ ^220^, ^221^, ^222^ ^]^ but with the advantage of apical‐basal compartmentalization of these cell types to recreate in vivo‐like interactions.

In addition, 2D organoid monolayer systems enable integration of various sensors (e.g., oxygen, chemical, electrical, etc.) that are not compatible with 3D organoids. For example, transepithelial electrical resistance (TEER) measurements facilitate the observation of barrier integrity.^[^ ^223^ ^]^ Furthermore, organoid‐derived monolayers have been shown to exhibit a degree of in vivo‐like compartmentalization of epithelial cells, recapitulating in vivo‐like clustering of lgr5+ stem cells and lgr5‐ cells into domains.^[^ ^224^ ^]^ The emergence of crypt and villus‐like domains within these monolayers emphasizes the model's close resemblance to in vivo morphogenesis and its power over conventional cell lines. A significant advantage of organoid‐derived monolayers in the context of host‐pathogen interaction is the ease of apical and basal access, enabling direct, specific pathogenic or pathogen‐derived metabolite delivery to the cells.^[^ ^215^, ^225^ ^]^ Inability to efficaciously isolate representative gut flora from primary samples, as well as difficulties in providing the anaerobic conditions necessary for obligate anaerobic bacterial culture while providing sufficient oxygen to the intestinal epithelial cells, has been a consistent problem. In vivo, a sharp oxygen gradient is generated by the high consumption of oxygen by IECs, paired with the mucus and ECM layers which define the compartments for distinct niches of bacteria.^[^ ^226^ ^]^ However, much like 3D self‐assembled organoids which naturally form a hypoxic lumen, 2D monolayers can be modified to integrate anaerobic chambers to the luminal side, thus enabling the co‐culture of anaerobic bacteria. Various modified cell culture inserts for this system exist,^[^ ^227^, ^228^ ^]^ which have been used to demonstrate successful short‐term co‐culture of facultative and obligate anaerobes, albeit limited to sporulating obligates. Furthermore, imposition of oxygen gradients can further approximate in vivo‐like conditions, which have been established to result in depressed stem cell activity and improved restriction of dividing stem cells to crypt regions of 2D shaped monolayers, recapitulating natural compartmentalization in the gut.^[^ ^227^ ^]^ The importance of replicating in vivo oxygen concentrations in in vitro systems is often ignored.^[^ ^229^ ^]^ Oxygen has been shown to help regulate gut homeostasis through multiple pathways, including through hypoxia‐inducible factor (HIF). Oxygen levels also influence fermentation, and the production of short chain fatty acids (SCFAs) by bacteria which are consumed by the epithelial cells can regulate oxygen consumption.^[^ ^230^ ^]^ One useful method of maintaining oxygen gradients is by culturing models under continuous flow where the apical and basolateral regions are distinct. In such a case, a hypoxic environment can be maintained apically which is suitable for anaerobic bacterial culture, and the basolateral region can remain oxygenated such that the mammalian cells continue to be cultured at physiological oxygen concentrations.

Several shortcomings plague the organoid‐derived 2D monolayer system however. Of significant note is insufficient mucus production, and the absence of fluid flow and peristalsis. In vivo, the SI and colon possess a mucus layer^[^ ^210^ ^]^ which has been implicated in modulating epithelial‐bacterial interactions^[^ ^231^ ^]^ and is negatively impacted by pathogens^[^ ^232^ ^]^ and disease.^[^ ^233^ ^]^ Mucus production in 3D organoids is restricted by luminal size, while cell culture inserts are limited by thin mucus production (<36 µm),^[^ ^215^ ^]^ and while air‐liquid interface (ALI) systems improve this (≈381 µm),^[^ ^234^ ^]^ generated mucus layers remain significantly below in vivo thicknesses.

Furthermore, organoids derived from patients with IBD fail to maintain an inflammatory phenotype once in culture^[^ ^235^ ^]^ – as IECs respond differentially to bacterial metabolites (e.g. butyrate^[^ ^236^ ^]^) depending on the context of inflammation, further understanding of external factors influencing host gut‐pathogen interactions is necessary.

The lack of fluid flow in organoid‐derived 2D monolayer systems is conducive to overgrowth of co‐cultured microbes, which can negatively impact epithelial cells due to rapid nutritional depletion, overcrowding, and waste metabolite overaccumulation. This may result in 2D monolayer‐bacterial co‐cultures that more closely resemble pathogenic GI bacterial overgrowth^[^ ^237^ ^]^ and may not be truly representative of in vivo host‐pathogen interactions and responses. This is perhaps unsurprising, considering that GI bacterial overgrowth often arises because of defects in peristalsis^[^ ^238^ ^]^ or due to inflammation and the formation of fistulae.^[^ ^239^ ^]^ This problem may be combatted through physical separation of bacteria from cells, or alternatively through introduction of fluid flow,^[^ ^240^ ^]^ which has been shown to elicit crypt‐villus like 3D microstructural patterning.^[^ ^241^ ^]^

#### 4.2.2 3D Models

3D models more closely mimic the characteristics of the in vivo physiological system by emulating the tissue microenvironment including cell‐to‐cell and cell‐to‐ECM interactions. This is most simply achieved by modifying the underlying substrate with an ECM‐like coating including fibronectin, collagen, laminin, and hyaluronic acid.^[^ ^212^, ^213^ ^]^ Such proteins are found in the basement membrane of barrier tissues and have physical properties that far more closely mimic the ECM than conventional plastic cultureware. Cells can form attachments to this matrix which assists with organizing the cells into a particular structure. In addition to traditional planar 2D monolayers, advancements have been made in developing shaped scaffolds for monolayer formation. Scaffolding made of biocompatible material (e.g., collagen) has been used to guide and shape dissociated organoids into crypt‐villi‐like structures, which have been suggested to promote more specialized differentiation, as well as formation of natural chemical gradients that may be found in vivo.^[^ ^124^, ^242^ ^]^ The 3D in vitro morphogenesis of the intestinal epithelium with crypt‐villi structures has been demonstrated on gut‐on‐a‐chip platforms and also on cell culture inserts, the former of which will be discussed in the gut‐on‐a‐chip section. The culturing of Caco‐2 cells and intestinal organoids on cell culture inserts under apical and basolateral flow has been demonstrated to form a 3D morphology that is absent when the basolateral flow is ceased.^[^ ^241^ ^]^ It is posited that this morphogenesis is controlled by a chemical gradient of Wnt agonist DKK‐1 and flow‐induced effects on the receptor FZD9.^[^ ^241^ ^]^ Shin and Kim have since produced a protocol, based on this mechanism, demonstrating the establishing of these 3D intestinal layers in simple cell culture insert setups in addition to more complex organ‐on‐a‐chip platforms.^[^ ^243^ ^]^

**Figure 5:**
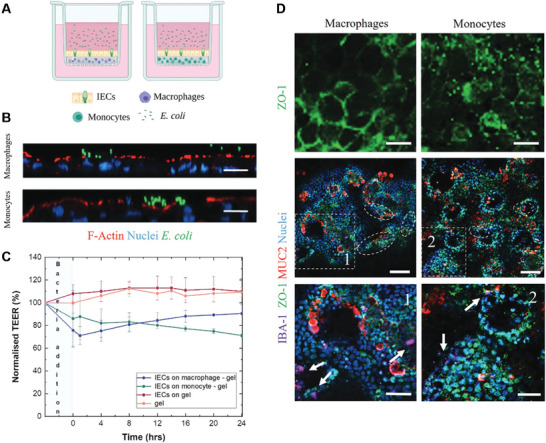
A) schematic demonstrating the setup of the model using a collagen matrix on a cell culture insert. B) Ortho‐views of confocal imaging showing *E. coli* interfaced with the cell intestinal epithelial cell (IEC) monolayers after 24 h (scale bar 20 µm). C) Normalized TEER values of the model variants once incubated with *E. coli*. D) Confocal images of IEC 24 h post‐incubation with *E. coli* (scale bar 20 µm). Macrophages and monocytes are labelled with IBA‐1. Images 1 and 2 are magnified from white dashed areas in the middle panel (scale bar 50 µm). Reproduced with permission.^[^ ^244^ ^]^ Copyright 2022, Wiley.

Other 3D tissues models have been developed within the confines of conventional cell cultureware – including culture inserts. Moysidou et al. developed a 3D system to monitor host‐parasite crosstalk on a cell culture insert.^[^ ^244^ ^]^ This included a Caco‐2 and HT29‐MTX co‐culture seeded above monocytes or macrophages (induced by PMA treatment of THP‐1 cells) within a collagen matrix (**Figure** **5**). Upon incubation with *E. coli*, both macrophages and monocytes are found to migrate through the collagen matrix and infiltrate the IEC barrier and the TEER response monitored. This model was further used to investigate the effect of the excretory‐secretory products (ESP) of the parasitic worm *T. circumcincta* when co‐incubated with *E.coli* to simulate the host‐microbiome‐parasite interaction. Cytokine profiles show a downregulation of IL‐4, IL‐10 and IP‐10 when treated with ESP compared to the *E. coli*‐only condition. The authors noted a pronounced increased in IL‐1b, IL‐8 and TNF‐α in the macrophage model compared to the monocyte model irrespective of whether ESP was present thus validating the use of macrophages within the system. Such models will be important for modelling the host‐microbiome and can be advanced by further mimicking the physiological system by incorporation of a range of bacterial species of the microbiome and the establishment of a similar O_2_ gradient found in vivo.

#### 4.2.3 Organoid Models

Apical‐basal compartmentalization within 3D self‐assembled organoids have proven powerful in investigating distinctly apical or basal invasive pathogen‐host interactions within the gut, such as in the case of *Salmonella*,^[^ ^245^ ^]^ *E. coli*,^[^ ^215^ ^]^ *and Listeria Monocytogenes*,^[^ ^246^ ^]^ as well as commensal interactions with the gut epithelia. In addition, gut‐specific parasitic^[^ ^247^ ^]^ and viral infections including rotaviruses^[^ ^187^ ^]^ and noroviruses,^[^ ^86^ ^]^ the latter of which has been difficult to culture robustly and induce GI invasion in vitro yet, have been recapitulated in organoids. Interestingly, bacterial pathogens such as Enteroaggregative and Enterohaemorrhagic *E. coli* (EACC and EHEC) have shown greater adherence to primary biopsy‐derived organoids over immortalized cell lines,^[^ ^215^ ^]^ while repeated *E. Coli* microinjections have been shown to induce colorectal cancer‐like mutations in organoids,^[^ ^248^ ^]^ demonstrating improved recapitulation of in vivo interactions compared to classic cell lines.

Despite such advantages of organoid‐based platforms over classic cell line‐based models, several prominent issues remain at hand. Organoids derived from biopsies, as per characteristic of primary samples, remain highly variable, which is paired with high batch‐to‐batch variability of organoid media and surrounding ECM (e.g., Matrigel).

Gut‐pathogen interactions have been conventionally modelled with organoids in their native, 3D structure. Stable co‐cultures of gut bacteria have been demonstrated in the context of organoids – either via direct coculture^[^ ^245^ ^]^ or luminal introduction of bacteria into 3D organoids.^[^ ^249^, ^250^ ^]^

**Figure 6:**
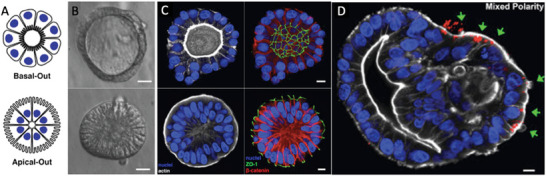
A–C) Normal and Inverted organoid. A) illustration and B) image via modulation contrast microscopy. C) Image via confocal microscopy. Nuclei in blue, actin in white, ZO‐1 in green, and β‐catenin in red are shown. D) S. typhimurium‐mCherry selectively invades the exposed apical surface (green arrows) of a mixed polarity enteroid. All scale bars are 10 µm. Figures demonstrate axis‐specific host‐pathogen interactions recapitulated with an organoid platform. Adapted with permission.^[^ ^253^ ^]^ Copyright 2019, Cell Press.

Although straightforward, direct bacterial‐organoid co‐culture displays restricted apical (luminal) access of organoids and is thus limited in its use to basal (mucosal) interactions (**Figure** **6**A). Moreover, in vivo, IECs rarely come into direct contact with gut microbiota, which further limits this model to pathogenic interactions. Instead, addition of bacterial metabolites via introduction through the media, including those secreted by commensals and pathogenic strains, can be used with organoids to investigate the effects of these bacteria while circumventing issues presented with bacterial co‐culture,^[^ ^251^ ^]^ although this may not always be an option for invasive pathogenic bacteria.

In contrast, the lumen of organoids provide relatively hypoxic conditions, which enables co‐culture of sporulating anaerobic bacteria such as *C. Difficile* with microinjected bacteria surviving up to 12 h in culture.^[^ ^121^, ^252^ ^]^ However, stable, long‐term co‐culture of obligate nonsporulating anaerobic bacteria via microinjection has proven difficult due to oxygen presence in the lumen, and despite studies demonstrating successful maintenance of non‐sporulating obligate anaerobes such as *Bifidobacterium* in the lumen of colon‐derived organoids,^[^ ^252^ ^]^ resident bacteria are suggested to rapidly lose viability and further improvements are necessitated.

Although the microinjection platform has shown significant improvements in protocol standardization, including injection volumes^[^ ^254^ ^]^ and fluorescence‐dye guided injections,^[^ ^255^ ^]^ the system remains cumbersome and is plagued by significant injection‐by‐injection differences and steep training curve in addition to fundamental problems.

Studies have demonstrated the potential for reversing luminal‐apical polarization through eversion^[^ ^253^ ^]^ (Figure 6), which could enhance the effective delivery of substances to the apical side of organoids. Nevertheless, this eversion protocol demands approximately three days of organoid maintenance in suspension on low‐attachment plates, resulting in limited experimental timeframes, decreased cell viability, and increased organoid‐to‐organoid adhesion due to the absence of ECM. Moreover, simultaneous apical‐basal control and electrical measurements across the organoid barrier remain a significant challenge within the context of spherical self‐assembling 3D organoids, which are inherently low throughput. Although high‐throughput microinjection platforms have been developed,^[^ ^252^ ^]^ they nevertheless possess limitations in incorporating barrier measurements and generating authentic crypt‐villi structures, thus leaving room for further advancements in this area.

Alternatively, microbes or their metabolites may be introduced to the lumen of organoids via fragmentation, which involves mechanical disruption of the organoids followed by addition of microbes, after which the disrupted organoids reassemble into 3D structures with the foreign material now entrapped within the lumen.^[^ ^256^, ^257^ ^]^ While fragmentation can be considerably higher throughput with lower demands on the specialized training required as in microinjections, it nevertheless demonstrates difficulties concerning infection efficacy, control of bacterial/pathogenic payload, and uncontrollable non‐specific basal‐pathogenic interactions due to the random nature of bacterial uptake during organoid reassembly. To combat these persistent problems with 3D self‐assembling organoid systems, several in vitro models using organoid‐derived cells attempt to resolve these issues. We are of the opinion that patient biopsy‐derived organoids offer some of the best opportunities for replicating in vivo biology due to the presence of all the relevant cell types which are not possible to fully replicate with cell lines.

#### 4.2.4 Ex Vivo Models

In contrast to the recent developments in in vitro sensing, the gold standard for ex vivo technologies is the aforementioned technique called the Ussing chamber.^[^ ^258^ ^]^ While the focus of this review is in vitro models, there are aspects of *ex vivo* work that are useful to adopt. The Ussing chamber works by recording current flow over a barrier such as a cell layer or tissue piece. There is a voltage and a current sensing electrode on each side of the tissue, with an electrolyte to complete the circuit and allow for ion transport. The tissue is clamped at 0 V, and the short circuit current needed to maintain that voltage is measured. By increasing the voltage clamp to above 0 V and measuring the current, the TEER can be calculated. There are, however, limitations with Ussing Chambers, notably the complicated sample preparation and therefore low throughput of results.^[^ ^259^ ^]^

In addition to their previously discussed utility in handling tissue segments, Ussing chambers can also prove beneficial when working with cells cultured on inserts. Jafari et al. investigated the interaction of the *Clostridium difficile*, a diarrhoea causing pathogen, on T84 cells during standard (5% CO_2_) and anaerobic conditions, finding a greater reduction in TEER during the latter.^[^ ^260^ ^]^ The advantage of the Ussing Chamber was in having an anaerobic apical compartment, while the basal compartment was aerobic. Using a similar technique, T84 cells in Ussing chambers were used to monitor the toxin activity of *Bordetella pertussis* on the epithelium.^[^ ^261^ ^]^

### 4.3 Emerging Models

The term ‘emerging’ refers to models which incorporate additional design, engineering or sensing principles beyond that of standard cell cultureware or well‐established setups. This includes reconfiguring culture surface design geometry or materials, incorporating flow, and introducing in‐line sensors for live monitoring, for example.

#### 4.3.1 Gut‐On‐A‐Chip

**Figure 7:**
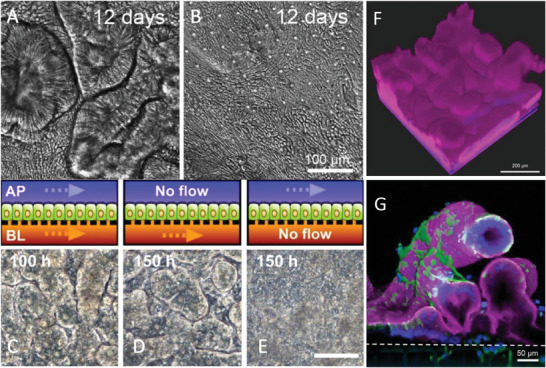
A,B) Differential interference contrast images of primary intestinal epithelium cultured on chip with HIMECs for 12 days under continuous and absent basal flow, respectively. C–E) Phase contrast images of intestinal epithelium cultured on chip under both apical and basal flow, basal‐only flow and apical‐only flow, respectively. Scale bar 50 µm. F) 3D reconstruction of confocal IF micrographs primary organoid‐derived epithelium under continuous flow. G) Confocal IF micrographs of vertical cross section under flow. Markers for F‐actin (magenta), DAPI (blue), Muc5AC (green). Figures demonstrate formation and increase in crypt‐villus morphogenesis under set flow conditions. Adapted with permission.^[^ ^216^, ^241^ ^]^ Copyright 2018, 2019, Springer Nature, Cell Press.

Derived from gut‐on‐chip platforms conventionally incorporating cell lines, organoid‐on‐chip systems have been developed to combine differentiated, in vivo‐like organoid‐derived cells with microfluidics to recreate cyclic compression and intestinal fluid flow via vacuum chambers and microfluidics. The system architecture used is the same as for cell lines and at their simplest, such models are microfluidic channels with an two inlets and two outlets for media flow across an apical and basolateral region compartmentalized by a porous membrane onto which cell monolayers are grown. Compared to conventional 3D organoids, such platforms have been shown to better approximate in vivo gene expression patterns and show improved crypt‐villus like patterning^[^ ^216^ ^]^ (**Figure** **7**). Moreover, vacuum chambers have been used to mimic peristalsis, in which pulsatile flow has been suggested to be essential in preventing bacterial overgrowth.^[^ ^262^ ^]^

**Figure 8:**
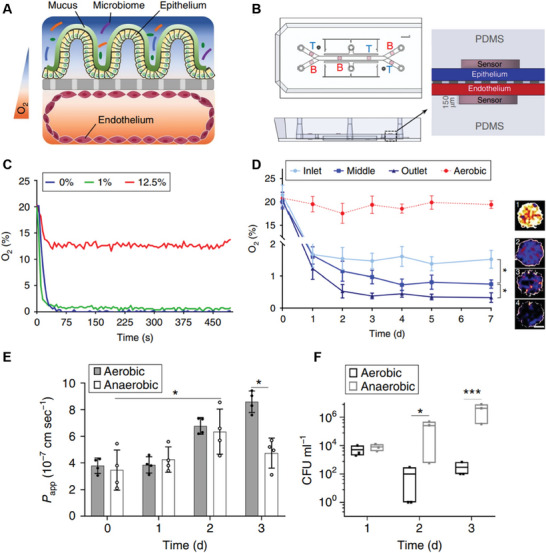
A) Microfluidic Organ Chip demonstrating an oxygen gradient with lower oxygen in the crypts. B) Schematic of the flow chip showing the oxygen sensors, inlets, and outlets. C) Sensitivity of oxygen sensors when exposed to defined oxygen levels. D) Oxygen concentrations in the aerobic chis (red) and at different locations in the anaerobic chips (blue). Scale bar = 200 µm. E) Changes in apparent permeability in aerobic and anaerobic conditions by quantifying the transport of Cascade blue. F) Colony forming units (CFU) per millilitre of *B. fragilis* cultured in both aerobic and anaerobic conditions. Reproduced with permission.^[^ ^264^ ^]^ Copyright 2019, Springer Nature.

However, reproducibility of in vivo‐like pathogen function in aerobic microfluidic systems have been called into question due to altered pathogenicity of microbes under disparate environmental oxygen concentrations.^[^ ^263^ ^]^ While such conventional ‘x‐on‐chip’ platforms have been limited in direct co‐culture with microbes due to their incompatibility with obligate anaerobes, recent developments have demonstrated successful co‐culture of microbes under anaerobic conditions on an organoid‐on‐chip system.^[^ ^264^ ^]^ Jalili‐Firoozinezhad et al. developed a fluidic device with integrated oxygen sensors using quenched fluorescence particles to monitor dissolved oxygen within the cell culture media (**Figure** **8**A–D). Microaerophilic and anaerobic environments could be maintained to better replicate the oxygen levels found in vivo which varies between 5 – 0.5% from the duodenum to the colon.^[^ ^264^ ^]^ The authors demonstrate the importance of maintaining an anaerobic environment by showing the difference in permeability of the established cell model (Figure 8E) in addition to the growth of *Bacteroides fragilis*, an obligate anaerobe, which exhibits a CFU ≈10^4^ times greater in anaerobic conditions than aerobic conditions after three days (Figure 8F).

In contrast to direct monolayers, engineered scaffolds enable recapitulation of the 3D crypt‐villi structure and may be made of ECM‐protein coated PDMS,^[^ ^265^ ^]^ micropatterned collagen,^[^ ^266^, ^267^ ^]^ hydrogels,^[^ ^268^ ^]^ and various other ECM substitutes. Such systems have been shown to demonstrate in vivo‐like compartmentalization of epithelial cell types, as well as characteristic behavior such as migration.^[^ ^266^, ^267^ ^]^ While many current systems fail to recreate the complex composition of the ECM, strides have been made to better recreate such environments, including micro/nanofibrillar biomaterials,^[^ ^269^ ^]^ recombinant‐DNA based artificial protein polymers,^[^ ^270^ ^]^ and other platforms which recreate identified key ECM interactions.^[^ ^271^ ^]^ A potential drawback of gut‐on‐a‐chip systems is that the surface effects are largely dominating over volume effects in microfluidic channels meaning little mixing is experienced in the bulk of the volume of the fluid. Furthermore, the use of PDMS in fabricating such devices can be detrimental due to the potential for it to absorb hydrophobic biomolecules, including drugs, from the media thus reducing the effective concentration of solutes and affecting reproducibility.^[^ ^272^ ^]^ Other materials are available, including polystyrene that is ubiquitous with cell culture, however, difficulties exists with fabrication.^[^ ^273^ ^]^ Berthier et al. believe that the onus is on the engineers to overcome such challenges and to provide options and solutions that work for biologists,^[^ ^274^ ^]^ and indeed, we agree that model design should be driven by the biological question being addressed.

#### 4.3.2 Cell Membrane Models

Biological membranes enable compartmentalization of intracellular and extracellular niches and serve key functions in cellular interactions with the environment rendering them targets for a multitude of pathogens and drugs alike. Since the studying of membrane properties and reactions in their native surrounding is challenging, artificial or native lipid bilayers have been coupled to various substrates to form supported lipid bilayers.^[^ ^275^ ^]^ Such supported membranes model cell surfaces in vitro^[^ ^276^, ^277^ ^]^ and have the advantage of being compatible with the application of surface sensitive techniques for studying membrane properties and biomolecular interactions with molecules or pathogens. Additionally, these systems depict new biosensor concepts for diagnostics and treatment.^[^ ^278^, ^279^ ^]^

**Figure 9:**
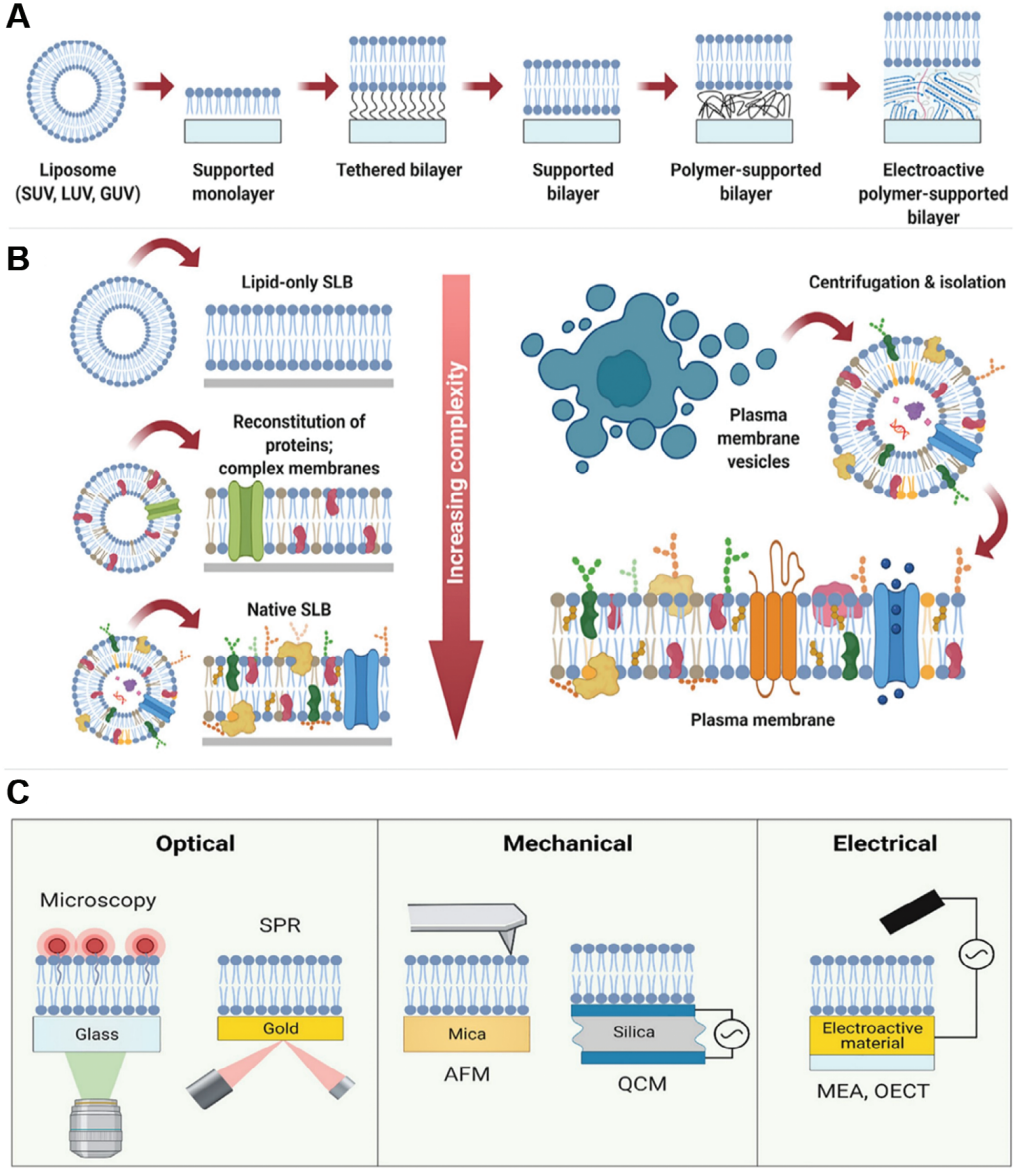
A) Types of lipid membrane on varying substrates. B) Increasing biological relevance of supported lipid bilayers. C) Methods for characterizing and monitoring the integrity of supported lipid bilayers. Adapted with permission.^[^ ^288^ ^]^ Copyright 2022, Cell Press.

Biological membranes are lipid bilayers featuring embedded proteins and attached carbohydrates.^[^ ^280^ ^]^ A variety of supported lipid bilayer systems has been descripted including freestanding black lipid membranes and integrated, freely supported, or polymer supported bilayers (**Figure** **9**).^[^ ^275^, ^281^ ^]^ Alongside monolayer transfer based on the Langmuir‐Blodgett technique^[^ ^282^ ^]^ and solvent‐assisted deposition,^[^ ^283^ ^]^ assembly of supported lipid bilayers is commonly achieved by a process called vesicle fusion.^[^ ^284^, ^285^ ^]^ Vesicle fusion is comprised of the absorption, rupture, and spreading of small lipid vesicles on surfaces to form a continuous film. Single‐ or multi‐lipid compositions as well as native cell membranes^[^ ^286^ ^]^ have been employed to fabricate supported lipid bilayers. Those native membranes can be extracted from a variety of cells via cell lysis or through induced proteoliposome release from cells in a process called blebbing.^[^ ^277^ ^]^

Supports are selected to meet requirements of the intended investigation technique such as transparency in case of optical measurements^[^ ^287^ ^]^ or conductivity in electrical studies.^[^ ^288^ ^]^ This has led to the fabrication of supported lipid bilayers on solid substrates like glass or silicon as well as on polymer cushions separating solid support and membrane.^[^ ^289^, ^290^, ^291^ ^]^ The latter is especially promising for representing membranes in their native configuration due to preservation of the membrane's natural fluid character allowing for diffusion and dynamic rearrangement of lipid molecules and proteins, and prevention of transmembrane protein denaturation.^[^ ^276^ ^]^ As such, conducting polymers are a useful subclass of polymer supports because they provide these advantages while also being transparent and electrically conducting.^[^ ^292^ ^]^

Regardless of the type of membrane and support, it is crucial to create continuous and defect‐free bilayers to allow for high quality investigations by applying a variety of surface sensitive techniques including surface plasmonic resonance spectroscopy, atomic force microscopy, neutron scattering, and quartz crystal microbalance with dissipation monitoring.^[^ ^288^ ^]^ Additionally, optical fluorescence microscopy and electrochemical impedance spectroscopy (EIS) provide tools to characterize membrane integrity and to study binding events as well as membrane‐pathogen interactions.^[^ ^293^ ^]^

Examples of host‐pathogen interaction studies featuring supported lipid bilayers comprise interactions of bacterial toxins with cell membranes and virus fusion events. Since bacterial toxins mainly act on the cell membrane by introducing pores or hydrolyzing lipids,^[^ ^294^, ^295^, ^296^ ^]^ electrochemical impedance spectroscopy can be leveraged to monitor changes in resistance due to the incorporation of pores or disturbances in membrane integrity.^[^ ^126^ ^]^ Furthermore, total internal reflection fluorescence (TIRF) microscopy has been used to investigate the importance of ligand presentation in membranes and shown the influence of ligand clustering on toxin binding.^[^ ^297^ ^]^ Some viruses interact with the cell membrane in a process featuring a binding and subsequent membrane fusion event. The specific binding of virus like particles to certain membrane component domains was investigated on supported lipid bilayers^[^ ^298^ ^]^ and the dynamics of the virus particle fusion process observed in another study.^[^ ^287^ ^]^

The relevance of studying and understanding virus‐cell interactions was further emphasized by the outbreak of the COVID‐19 pandemic and need for reliable platforms to accelerate the development of drugs and vaccines for combating viruses. Alongside common respiratory complications after SARS‐CoV‐2 infection, up to one fifth of patients show gastrointestinal and hepatic symptoms.^[^ ^299^ ^]^ This phenomenon can be explained when considering the specific binding of the viral envelope spike protein to the host's ACE2 (angiotensin‐converting enzyme 2) receptor in combination with high expression levels of ACE2 receptors, e.g., in lungs, endothelial cells, and liver, with the highest levels found in the brush border of intestinal enterocytes^[^ ^299^ ^]^

**Figure 10:**
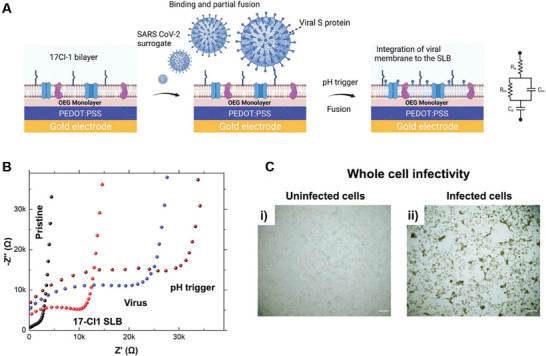
A) Schematic representation of Mouse Herpes Virus (MHV) fusion on a supported lipid bilayer (SLB) interfaced with conducting polymer electrodes. B) Nyquist plot showing the impedance of the electrode, supported lipid bilayer, virus addition and pH‐triggered fusion. C) Cytopathic effect of MHV on 17‐Cl1 murine cell line i) before, and ii) post infection. Scale bars 100 µm. Reproduced with permission.^[^ ^300^ ^]^ Copyright 2023, Wiley.

In this case, supported lipid bilayers provide compelling in vitro alternatives to state‐of‐the‐art cell‐based assays and animal testing. As such, Kallitsis et al optimized a platform for interfacing native membrane‐derived supported lipid bilayers with electrodes to monitor membrane integrity using electrochemical impedance spectroscopy (EIS) (**Figure** **10**).^[^ ^300^ ^]^ The attachment of the SARS‐CoV‐2 surrogate murine hepatitis virus (MHV‐A59) to a biomimetic SLB derived from the 17‐Cl1 murine cell line was observed through an increase in membrane resistance. Upon the addition of a pH 5.5 trigger, mimicking host cell cytosol, a further increase in membrane resistance is observed indicative of viral fusion. Both endosomal and non‐endosomal pathways can be investigated with this technique. When the relevant receptors are not present no fusion occurred demonstrating the specificity of this platform. Furthermore, this method could be used to discriminate between infectious and non‐infectious particles and prove useful for drug screening and vaccine development. However, not all membrane‐virus interactions show specificity as identified for the SARS‐CoV‐2 spike protein, which on its own introduces bilayer degradation in the presence and absence of the ACE2 membrane receptor.^[^ ^301^ ^]^ Importantly, membrane bioelectronic sensing can be extremely sensitive resulting in the detection of single binding events in membrane pores when applied in stochastic biosensing.^[^ ^302^ ^]^

Supported lipid bilayers are powerful in vitro platforms for accelerating and broadening fundamental and mechanistic understanding of biological membranes and facilitate development in diagnostics, treatment, and prevention of infectious diseases.

#### 4.3.3 Mucus models

**Figure 11:**
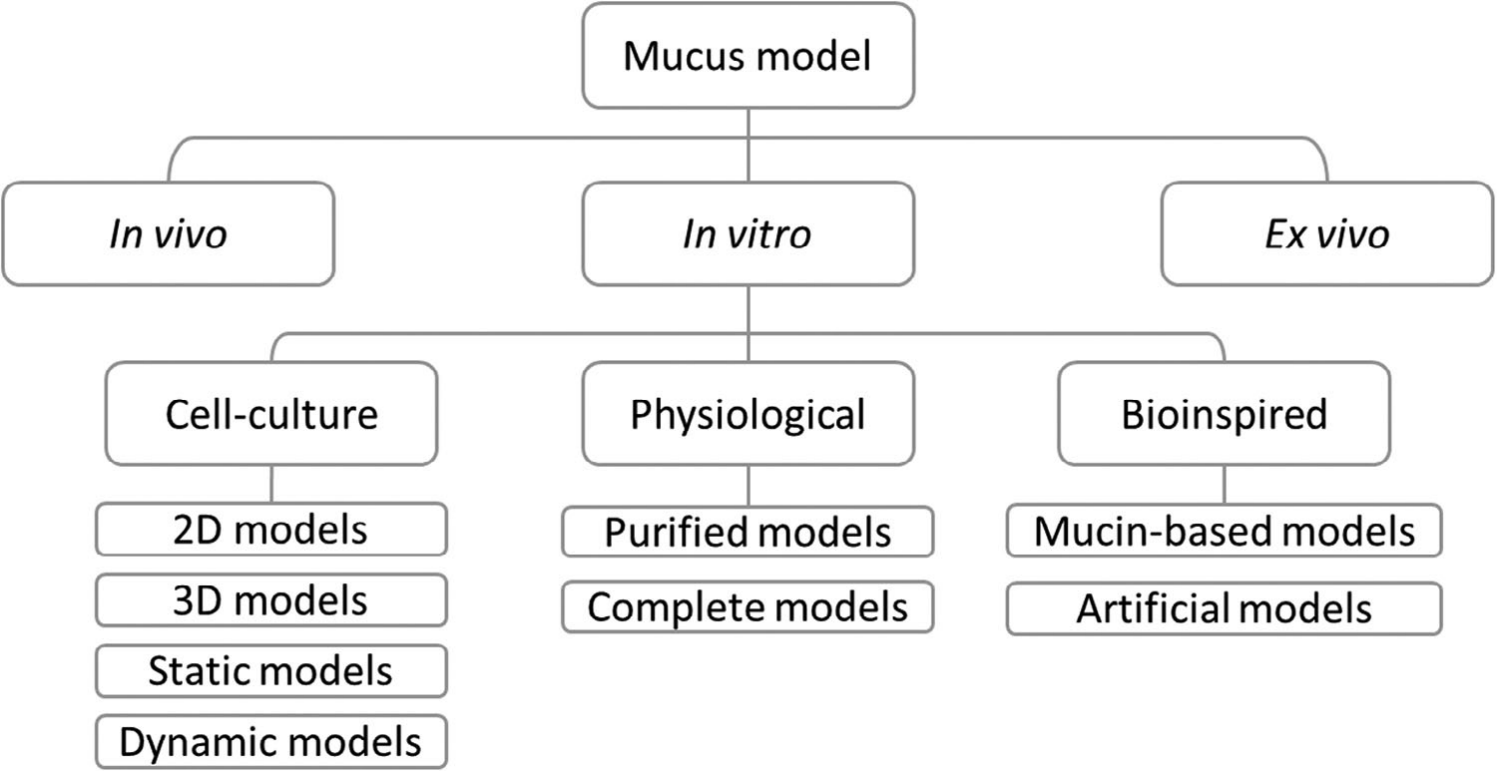
Classification of mucus models in the literature. Adapted with permission.^[^ ^304^ ^]^ Copyright 2019, Royal Society of Chemistry.

The integrity of the mucus layer plays an important role in protecting the gastrointestinal lining from digestive enzymes, commensal bacteria, and pathogens. This section will discuss mucus composition and structure, gastrointestinal mucus models, the effect of microbes and pathogens on these mechanisms and how various models have been used to monitor pathogen binding, colonization, and invasion. A hierarchical structure of the mucus models commonly used in literature is presented in **Figure** **11**.

##### Mucus Composition and Structure

Native mucus is mainly composed of 90–95% water, 5–6% proteins, 4% mucins, 2–4% lipids, and 0.02‐0.8% DNA molecules.^[^ ^303^, ^304^, ^305^ ^]^ Among all these components, mucins are the primary contributor to hydrogel formation and their structure and chemical properties have been discussed by numerous studies.^[^ ^304^, ^306^, ^307^, ^308^, ^309^ ^]^ Broadly, mucins are classified into two groups: transmembrane mucins that are synthesized and tethered to cell membranes, and gel‐forming mucins that are secreted by goblet cells. The former is primarily for cell protection purposes, while the latter can form a mucus hydrogel dictate the mucus’ viscoelastic properties. The type and structure of secreted mucins vary along the gut (MUC5AC in the stomach, MUC2 and MUC6 in the intestine), but key features are conserved: mucin monomers contain a linear structured backbone enriched with proline, threonine, and serine residues (PTS domains), and complex side chains which are highly O‐glycosylated. Studies show that mucins monomers are cross‐linked to form a complex 3D structure via disulfide bonds, which are supplied by cysteine‐rich von Willebrand factor (vWF)‐like domains.^[^ ^310^, ^311^ ^]^

Moreover, the thickness and pore size of the formed mucus vary along the intestine. The total mucus thickness in mice changes from ≈500 µm in the duodenum to 200 µm in the ileum.^[^ ^312^ ^]^ The intestinal mucus has a double‐layer structure with the inner dense layer having a pore size down to 0.5 µm.^[^ ^313^ ^]^

##### Gastrointestinal Mucus Models

To date, several studies have focused on the establishment of mucus models that can be employed to monitor the processes of both drug particles delivery and pathogen invasion to the GI tract. Indeed, the targeted delivery of drugs to the epithelium is particularly difficult and consideration has to be given to the materials of choice when designing an active biomaterial which is mucoadhesive or mucus penetrating, as the application dictates.^[^ ^314^ ^]^ Generally, there are three classes: in vivo, ex vivo, and in vitro mucus models and a summary is presented in **Table** **5**. The Loc‐I‐Gut model^[^ ^315^, ^316^ ^]^ is one of the most classic in vivo approaches in humans, using an intestinal perfusion technique that requires a multichannel tube with two balloons. Similar studies are also reproduced on rats but with single‐pass perfusion. Ex vivo models of intestinal mucus was first reported by Osiecka and co‐workers in 1985^[^ ^317^ ^]^ and improved in 1993,^[^ ^318^ ^]^ which utilized everted intestine rings to research the effect of drug particles.

**Table 5:** Advantages and disadvantages of in vivo, ex vivo, and in vitro mucus models.

Mucus Models	Advantages	Disadvantages	Ref
In vivo	Most closely mimic the natural physiological conditions of mucus	Animal sacrifices, ethical concerns High cost, low repeatability	[304, 315, 316]
Ex vivo	Allow various mucus models compared to in vivo studies Can either bound with tissue or excised separately	Animal sacrifices, ethical concerns High cost, low repeatability Delicate pore structures could be damaged during excision Loss of loose mucus layer after washing	[328, 329, 330]
In vitro			
Cell‐culture (HT29‐MTX and Caco‐2 coculture)	Controllable mucus secretion level Advanced culture system can mimic native intestinal 3D environment	Unable to replicate the double‐layer structure of native intestinal mucus HT29‐MTX lines cannot secrete intestinal MUC2	[119, 322, 323]
Purified mucus	Simple to prepare Mimic defensive properties against pathogens	Unable to replicate mucus‐like viscoelastic property of native mucus Initial structure cannot be recovered after rehydration	[319, 324, 325]
Mucin‐based mucus	Cross linked mucin‐based mucus shows comparable rheological properties, pore size and network structure with native mucus	Apply nonhuman commercial mucins that have different glycosylation Commercial mucins may contain adulterants that vary from batch to batch	[324, 326]
Bioinspired mucus	Comparable physical properties with native mucus Controllable mucus mechanical properties through raw material ratio	Only contain mucins, no other components (i.e., lipids, DNA, cellular debris)	[321]

**Figure 12:**
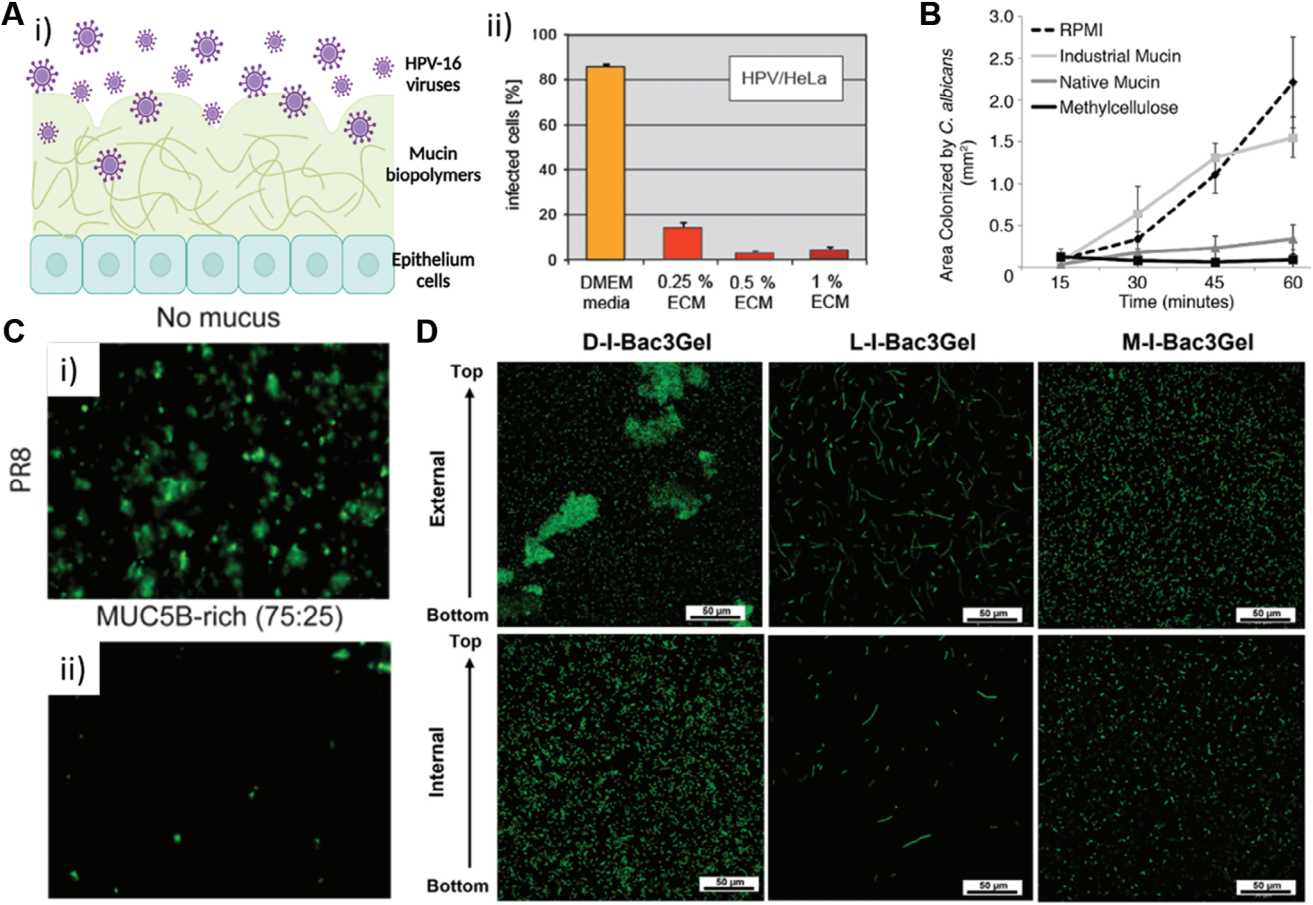
A‐i) Schematic representation of in vitro infection assay using mucin biopolymers, A‐ii) Percentage of HPV‐16‐infected HeLa cells protected by ECM hydrogel. Adapted with permission.^[^ ^319^ ^]^ Copyright 2012, ACS Publications. B) Quantification of C. albicans attachment to polystyrene protected under RPMI alone, industrial mucin, native mucin, or methylcellulose. C) IVA infected i) uncoated and ii) mucus‐coated BCi‐NS1.1 cells, green represents staining for IVA nucleoprotein. Reproduced with permission.^[^ ^320^ ^]^ Copyright 2021, ACS Publications. D) Infected *E. coli* in section of I‐Bac3Gel hydrogel mimicking loose mucus layer. Reproduced with permission.^[^ ^321^ ^]^ Copyright 2022, Elsevier.

However, in vivo and ex vivo approaches are limited by their low repeatability and animal sacrifices. Several in vitro models have been introduced to overcome these limitations. Co‐culture of HT29‐MTX and Caco‐2 is one of the models that combines the similar functionality of Caco‐2 cells with enterocytes and the mucus secretion capability of methotrexate‐treated HT‐29 cells.^[^ ^322^, ^323^ ^]^ By applying this cocultured cell line, García‐Díaz et al. successfully built a 3D in vitro model to mimic the in vivo situation, such as physiological dimensions and mechanical properties.^[^ ^119^ ^]^ By doing so, they found that mucus present in 3D topography can significantly modulate the interaction between pathogenic *E. coli* LF82 (AIEC) and the epithelium. Other researchers proposed an approach of using native mucins to form hydrogels from either a mixture of the components from native mucus, or rehydration of purified commercial mucins, i.e., porcine gastric mucin (PGM) and bovine submaxillary mucin (BSM).^[^ ^319^, ^324^, ^325^ ^]^ Lieleg and co‐workers used purified PGM to study the mucus barrier defensive properties against viruses such as human papillomavirus (HPV), and Merkel cell polyomavirus (MCV).^[^ ^319^ ^]^ They placed mucus samples on top of cells and counted the number of infected underlying cells after adding viruses at the mucus surface (**Figure** **12**A). Although this model successfully mimicked the mucus physical properties and prevent infections, it failed to reproduce the native mucus mechanical strength. Hence, several papers have since improved on this formulation either by adding a mechanical adjuvant such as the MC system models^[^ ^319^ ^]^ containing glycoproteins and methylcellulose, or by adding a cross‐linker such as a 4‐arm thiol‐polyethylene glycol (PEG‐4SH) to form a covalent network, as proposed by Duncan et al.^[^ ^326^ ^]^ The authors also investigated if mucus composition can affect its barrier function toward *influenza A* virus (IAV) by monitoring the diffusion rate of fluorescently labelled IAV in mucus samples, which had different MUC5B:MUC5AC ratio, via confocal laser scanning microscopy (CLSM) and particle tracking microscopy (PTM) in their follow‐up report (Figure 12B).^[^ ^320^ ^]^ Only a small fraction of IAV can penetrate through the mucus layer. Similar results were also found by Kavanaugh et al, who detected the colonization ability of *C. albicans* on the polystyrene plates with and without a 3D mucus layer, using not only MUC5AC but also MUC2 and MUC5B, which demonstrated the general effect of mucins on *C. albicans*.^[^ ^327^ ^]^ Phase‐contrast microscopy imaging illustrated that native mucins significantly reduce the *C. albicans* attachment (Figure 12C). Moreover, they also observed that mucins successfully downregulated the genes involved in cell adhesion (*ALS1* and *ALS3*).

In addition to the mucin‐based approach discussed above, another synthetic approach to produce bioinspired intestinal mucus model was proposed by Sardelli et al. in 2022.^[^ ^321^ ^]^ They formed hydrogel (I‐Bac3Gels) by mixing alginate with calcium carbonate and D‐(+)‐gluconic acid δ‐lactone to mimic the intestinal loose layer. The I‐Bac3Gels exhibited a homogeneous distribution of *E. coli* (ATCC 25922) (Figure 12D), which qualitatively is similar to the results of in vivo animal studies.

##### Bidirectional Interaction of Mucus and Microbiota

The composition of commensal microbiota can strongly modulate the intestinal mucus layer has been shown by a number of studies. For example, mucus in the small intestine needs to be detached from goblet cells which requires assistance of an enzyme called meprin β. This protease can only be activated under the control of gut microbiota.^[^ ^331^ ^]^ Furthermore, a murine model used by Jakobsson and co‐workers suggested that bacteria, such as *Allobaculum*, could improve the mucus layer protective function, while other bacteria, like *Proteobacteria*, have an antagonistic effect.^[^ ^332^ ^]^ One possible mechanism is that specific bacteria can induce the expression of host glycosyltransferase and therefore shape the glycan profile.^[^ ^333^ ^]^ Also, probiotic agents (e.g. *Lactobacillus species* ^[^ ^334^ ^]^) can stimulate the production of mucin and increase mucus layer thickness to improve the resistance to pathogens.

Conversely, the mucus layer can affect gut microbiota by providing specific attachment sites that only suitable bacteria can colonize successfully.^[^ ^308^ ^]^ For instance, *Acinetobacter spp* can penetrate through the mucus and colonize at the colonic crypts.^[^ ^335^ ^]^ Moreover, mucin glycans also serve as a direct source of carbohydrates for microorganisms. One distinct subset of intestinal microbiota, mucolytic bacteria, has the capability to digest mucin glycans via their glycan‐degrading enzymes, including glycoside hydrolases, sulfatases, and proteases.^[^ ^336^ ^]^ These enzymes can produce short chain fatty acids (SCFAs) that can also be partially utilized by colonocytes, which further confirms the mutualist relationship between the host and the commensal microorganisms.^[^ ^337^ ^]^

Pathogenic bacteria can also colonize in the intestine and cause infections by employing several strategies. They can not only degrade mucin glycans with enzymes but also use gluconeogenic nutrients flexibly. For example, *Vibrio cholerae* can utilize N‐acetyl‐D‐glucosamine and sialic acid compared to commensal bacteria.^[^ ^61^, ^338^ ^]^ Due to the presence of fimbriae, proteases, and other specific components, pathogens can easily penetrate through the mucus layer. For instance, *S. enterica serotype Typhimurium* and some uropathogenic *E. coli* can adhere the mucin glycans via the fimbriae,^[^ ^339^ ^]^ while *E. coli* LF82 can release a protease called VatAIEC to degrade mucins and decrease mucus viscosity. It is clear that the presence of mucus, and its absence, or presence of defects in disease states, is an important aetiological factor in intestinal infectious diseases.

## 5 Design Considerations for Advanced Monitoring

Conventional models are useful for understanding biological mechanisms with fixed timeframes and endpoints. Often the temporal response of a system under investigation is unknown and therefore continuous monitoring techniques can improve on existing approaches. A brief description of such monitoring techniques are discussed in addition to design criteria for incorporating these into advanced models.

### 5.1 Permeability

Increased permeability of the gut has been implicated in numerous gastrointestinal disease states. It is therefore essential that appropriate tools are developed to monitor and interrogate gut permeability.^[^ ^259^ ^]^ Due to the complex nature of cell‐cell contacts across the epithelium, different methods and probes can be used depending on the biological mechanism investigated. Multiple pathways for permeability of current and probes exist – including a transcellular and paracellular route, and therefore consideration for this should be made when deciding on a particular assay.

#### 5.1.1 Electrical

**Figure 13:**
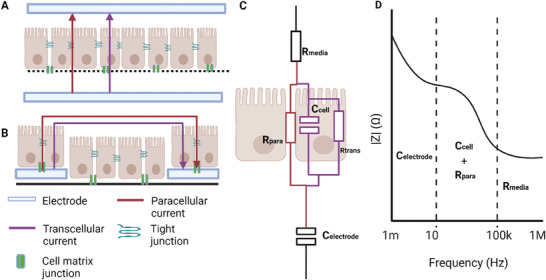
Current pathways between electrodes in different set‐ups. The transcellular pathway is through the cell membrane and the paracellular pathway is between cells. A) Current pathways with cells grown on a cell culture insert or tissues in an Ussing chamber with electrodes either side of the barrier. B) Current pathways with cells grown on an impermeable surface and electrodes on one side. C) Equivalent circuit model of the current pathways. D) Typical Bode plot of electrochemical impedance spectroscopy performed on a layer of barrier forming cells. Figure created in Biorender.com

Although barrier integrity and permeability can be measured through techniques such as immunofluorescence and spectroscopy, the main draw to electrical measurements is their inherently quantitative nature. By employing electrodes to induce a current through a cellular barrier and recording the resulting signal, as shown in **Figure** **13**A, it is possible to measure the transepithelial electrical resistance (TEER). TEER is a widely accepted gold standard for evaluating barrier integrity and permeability.^[^ ^340^ ^]^ A cellular monolayer can be modelled as an electrical circuit, which is commonly composed of a resistance due to the cell culture media, a paracellular resistance between the cells, a capacitance of the cell membrane and a transcellular resistance of the cell membrane (Figure 13C). The capacitance of the electrodes can also be included in the electrical circuit.

The most straightforward approach for TEER calculation involves using two electrodes to apply a constant current and record the voltage across the barrier. By applying Ohm's law and normalizing the result to the cellular area, the TEER can be determined. The main limitation of this method is that the result is the combined parallel resistance of the transcellular, and paracellular pathways. Commercial technologies include the Epithelial Volt/Ohm Meter (EVOM) for in vitro applications and the Ussing chamber for *ex vivo* experiments. The EVOM injects 12.5Hz current via a pair of silver/silver chloride chopstick electrodes, designed to sit in media either side of a cell culture insert. Its ease of use and quick measurement time have made it immensely popular among in vitro studies^[^ ^341^ ^]^ and, as such, the decrease in TEER caused by the apical introduction of harmful bacteria and toxins like *Salmonella typhimurium* ^[^ ^342^ ^]^ and *Listeria monocytogenes* ^[^ ^343^ ^]^ on rabbit small intestine tissue^[^ ^342^ ^]^ and Caco‐2 cells^[^ ^343^ ^]^ has been demonstrated. It is also used to monitor the increase in TEER during the apical addition of beneficial bacteria on Caco‐2 cells such as *Lactobacillus casei* ^[^ ^344^ ^]^ and *Lactobacillus plantarum*.^[^ ^345^ ^]^

Electrochemical impedance spectroscopy (EIS) represents another approach for determining the value of TEER.^[^ ^346^, ^347^ ^]^ In 1991, Giever and Keese pioneered the use of small currents to assess cellular barrier function and impedance.^[^ ^348^ ^]^ Since then, significant progress has been made with the development of commercially available technologies such as the CellZscope and electric cell‐substrate impedance sensing (ECIS).^[^ ^349^, ^350^ ^]^ Although impedance‐based measurements introduce greater electrical complexity compared to resistance‐based measurements, they permit the decoupling of the different resistances, and provide in an insight into the capacitive activity of the cells. EIS is non‐invasive, rapid, and allows for real‐time monitoring.^[^ ^351^ ^]^ This technique works by applying a small voltage AC frequency sweep between 10^−3^ and 10^6^ Hz and measuring the resultant signal.

**Figure 14:**
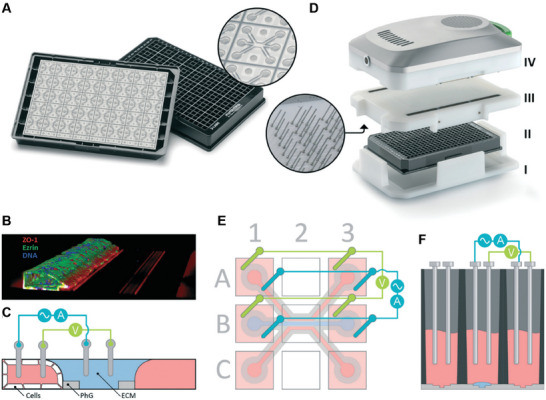
OrganoPlate and OrganoTEER device design. A) Shows 384 wells which connect 40 microfluidic chips. B) Confocal imaging of Caco‐2 cells culture within the device. C) Chip cross section. D) Exploded view of the system including the holder. E) Schematic of the electrode configuration of a single fluidic chip. F) Cross section of three wells with inserted electrodes. Reproduced with permission.^[^ ^355^ ^]^ Copyright 2021, Royal Society of Chemistry.

Such techniques are compatible with existing cell cultureware geometries, including permeable cell culture inserts. The real advantage with electrical techniques is that they can be integrated into most geometries and tissue engineering models that exist. Indeed, electrical monitoring of barrier integrity has been demonstrated on planar, 2.5D and 3D tissue engineered models using a membranous material as a support.^[^ ^352^, ^353^, ^354^ ^]^ Models that rely on artificial porous membranes are typically stifled by low throughput due to the complexity in manufacture. Higher throughput electrically‐compatible techniques have been developed that forego membrane supports by seeding directly against extracellular matrix.^[^ ^355^ ^]^ Nicolas et al. reported a model which connects 40 microfluidic chips on a plate layout for high throughput measurements (**Figure** **14**). This device design elegantly combines electrical measurement capabilities with microfluidics and perfusion techniques into a high throughput system. With less complex systems the measurement throughput can be increased through the use of an external multiplexer coupled to a potentiostat for automatic simultaneous or sequential measurements of independent samples.

Increasing further in complexity from the set‐ups described above, the disruption of a 2D barrier formed from a monolayer of cells can also be measured electrically by organic electrochemical transistors (OECTs).^[^ ^356^ ^]^ Two electrodes, the source and drain, are modulated by an additional electrode, the gate. The response time of the OECT is linked to cell barrier formation.^[^ ^357^ ^]^ OECTs offer an enhanced measurement gain and sensitivity but EIS offers increased ease in terms of fabrication and operation.^[^ ^358^ ^]^ Both techniques are often employed to measure the disruption of a Caco‐2 monolayer barrier, for example on the addition of *Salmonella typhimurium* ^[^ ^114^ ^]^ and *Faecalibacterium prausnitzii*.^[^ ^359^ ^]^

#### 5.1.2 Probe Assays

Barrier integrity of models can also be assessed as a function of direct diffusion of fluorescent dyes (e.g. Lucifer Yellow^[^ ^360^ ^]^), enzymes which give rise to fluorescent markers (e.g. HRP^[^ ^361^ ^]^), or those bound to semi‐permeable molecules (e.g. FITC‐Dextran^[^ ^362^ ^]^) which may be generally observed via fluorescence spectroscopy.^[^ ^363^ ^]^ Damage to cell membranes may be similarly observed, for example through LDH assays to identify leakage of LDH,^[^ ^364^ ^]^ an intracellular enzyme released upon cellular death or membrane damage,^[^ ^364^, ^365^ ^]^ in contrast to Lucifer Yellow, FITC‐Dextran, and HRP, which are specific to passive paracellular transport and thus provide quantification of tight junction leakiness.^[^ ^366^, ^367^, ^368^ ^]^

In order to incorporate such assays into models, the substrate onto which cells are cultured must be permeable in order to allow the diffusion or transport of such probes through. It is essential that the permeability of the substrate is significantly lower than that of the epithelial layers so that it does not become a major contributor to diffusional resistance of the probe used. It is also important that the substrate is chemically inert and does not physically interfere with the passage of the probe used. Accumulation of the probe on the substrate surface would interfere with the permeability measurements. Furthermore, being able to accurately and quantitatively detect such probes is essential. The flux of fluorescence‐based probes, for example, can be monitored using a fluorescence microscope or a plate reader.

Being able to measure within the confines of the existing setup without having to manually sample can be achieved by ensuring optical transparency or employing a continuous fluidic system to draw the sample out of the setup to measure externally. Such design considerations are important for high throughput and to ensure samples are not periodically disturbed during cell growth.

### 5.2 Optical Properties

The ability to observe biological processes unfold in real time allows us to visualize the mechanisms of life as well as elucidate fundamentals of diseases and their causes. The physical architecture of models can preclude imaging due to a lack of optical transparency often as a result of material selection, substrate thickness or poor design configurations. Fluorescent microscopy has long formed the backbone of live‐cell imaging,^[^ ^369^ ^]^ enabling tracking and quantification of intestinal pathogens^[^ ^370^, ^371^ ^]^ cellular components, cellular proliferation,^[^ ^372^ ^]^ and interaction dynamics within the in vitro model.^[^ ^373^ ^]^ Immunofluorescent microscopy provides an additional layer of specificity to this technique utilizing target specific antibody probes, allowing for tracking of specific molecules, including all pertinent molecules such as cytokines, chemokines, receptors, growth factors, DAMP/PAMPs, and other molecules to investigate factors which may affect pathogen invasion,^[^ ^374^, ^375^, ^376^ ^]^ as well as quantification and localization of bacteria and host cells.^[^ ^377^ ^]^

Imaging capability requirements strongly dependent on the type of sample being investigated. With an increase in physiological relevance demanded by researchers, models of a 3D architecture are increasingly being explored and as such the capabilities to adequate image and understand these models is essential. Pairing them with confocal^[^ ^378^, ^379^ ^]^ or 2 photon microscopy^[^ ^378^, ^380^ ^]^ provides enhanced spatial resolution as well as sharper visualization of specific layers. 2 Photon microscopy has a greater penetration depth than confocal or epifluorescence microscopy due the higher excitation wavelength. It also results in reduced light scattering, photobleaching and phototoxicity. If smaller structures are to be visualized, super resolution microscopy techniques such as STORM (stochastic optical reconstruction microscopy) and SIM (structured illumination microscopy) enable visualization of structures beyond the diffraction limit^[^ ^381^ ^]^ and thus subcellular structures, such as accumulation of plasma membrane gangliosides^[^ ^382^ ^]^ or even ubiquitination^[^ ^383^ ^]^ in context of pathogenic invasion and proliferation.

Spectroscopy provides a non‐invasive approach for quantifying relevant proteins, metabolites, and nucleic acids^[^ ^384^ ^]^ within in vitro intestinal host‐pathogen models via measurement of EM radiation interaction with matter, with following identification of molecular spectra which arises from molecule‐specific inelastic scattering of light. Conventional methods include infrared and Raman spectroscopy which identify nuclear vibrations, and UV/fluorescence spectroscopy for electronic excitations,^[^ ^385^ ^]^ which enables identification and quantification of key molecules as discussed above. In addition to small biomolecules, spectroscopy can be used to distinguish between injured and healthy cell types,^[^ ^386^ ^]^ antibiotic resistance in bacteria.^[^ ^387^, ^388^ ^]^ While conventional infrared spectroscopy demonstrates limited spatial resolution (>7 µm),^[^ ^389^ ^]^ modified systems such as optical photothermal infrared (O‐PTIR) super‐resolution imaging,^[^ ^390^, ^391^ ^]^ scattering scanning near‐field optical microscopy (s‐SNOM)^[^ ^392^ ^]^ and photothermal‐induced resonance (PTIR) improve this. Importantly, spectroscopy can be used to quantify activity of enzymes^[^ ^393^ ^]^ unlike PCR, providing a better evaluation of cell state.

Many of the aforementioned techniques require penetration of light at varying wavelengths through the sample. The underlying substrates which cells are grown on, or within, are often orders of magnitude thicker than the biological samples themselves. In order to adequately probe these systems, material engineering can be employed. It is well known that acrylic plastics and glass absorb light at short wavelengths and as such quartz or Pyrex is used to overcome this. High magnification imaging necessitates a shorter working distance. Substrates must be thin in order to adequately image at high magnification. Resins typically used in 3D printed microfluidics suffer from poor optical transparency and are not suitable for high magnification imaging applications but modifications to typical processes can improve the transparency.^[^ ^394^ ^]^ In the case of fluorescence imaging, material choice of housing and cell culture substrates is also important. ECM proteins, e.g. collagen and elastin, are often used as coatings and gel substrates for cell seeding but are known to auto‐fluoresce which could interfere with the imaging technique by producing high background signal.^[^ ^395^ ^]^ Similarly, common materials used for cell scaffolds are known to auto‐fluoresce but techniques including photo‐bleaching and quenching are being employed to limit this.^[^ ^396^ ^]^ Such considerations should be made by researchers when designing models that are compatible with the optical characterization techniques available to the end user.

### 5.3 Sampling Capabilities

Sampling is extremely important to determine the state of the biological system in a time resolved manner. Cell culture media contains biomarkers secreted by both the host and the pathogens under investigation. Metabolites produced by gut microbiota are heavily implicated in the regulation of inflammation^[^ ^397^, ^398^ ^]^ and host‐pathogen interactions^[^ ^399^ ^]^ and can mediate bacterial proliferation^[^ ^400^ ^]^ as well as virulence.^[^ ^401^ ^]^ Observation of metabolites enables assessment of the effects of pathogens on local bacterial and metabolite population,^[^ ^402^ ^]^ as well as impact on host cell activity.^[^ ^403^ ^]^ However, despite its power, metabolomics requires further insight into metabolite identification due to the large number of unclassified molecules from NMR/MS‐derived identification.^[^ ^404^ ^]^

A time resolved quantification of pathogens is useful and often performed by manually sampling determining colony forming units and plaque forming units in the case of bacteria and viruses, respectively. Being able to directly and automatically remove media in order to perform such quantification is useful and prevents disturbance to the system. Dynamic culture is therefore a useful tool – not only does this mimic the in vivo state and provide standardization of the cultivation process,^[^ ^405^ ^]^ it also allows the removal of pathogens and metabolites from the system for downstream characterization and quantification. Such measurements can be performed using conventional methods including mass spectroscopy (MS) and nuclear magnetic resonance (NMR), however advances in engineered biosensors have demonstrated the rapid, sensitive, detection of target molecules.^[^ ^406^ ^]^ Combining the advances in the field of tissue engineering with those in metabolite^[^ ^407^ ^]^ and pathogen^[^ ^408^ ^]^ sensing technologies would undoubtedly contribute to the development of useful in vitro models with quantitative, continuous and real‐time observations of the state of biological models.

Designing a model which is capable of integrating sampling capabilities is not trivial. Geometry and flowrate must be optimized to account for the shear force that is experienced by cells. More complex geometries face difficulties with determining the shear forces. There are also material considerations that must be made. While PDMS is the workhorse of the microfluidic field it is known to absorb hydrophobic molecules from media and leach uncross‐linked oligomers which could interfere with down‐stream measurements.^[^ ^409^, ^410^ ^]^ Engineering access ports for media replenishment and removal is necessary and care should be taken to ensure they are adequately sealed to maintain culture sterility. It is important that design configuration is compatible with other techniques and therefore due consideration should be made to incorporate permeability and optical transparency in order to create a tool that is versatile and provides the end user with multiple means of interrogating and understanding the biological system.

## 6 Conclusion and Future Outlook

It is clear from the literature that there are two main philosophies when developing in vitro models for investigating intestinal host‐pathogen interactions which need to balance physiological complexity with experimental simplicity. First, there is a push to develop models that more closely emulate in vivo biology – in function and morphology. This is only likely to increase due to coinciding with the FDA's clarification of their requirement that drugs be tested in rodent and non‐rodent animal models prior to clinical trials in the FDA Modernization Act 2.0. From December 2022, drugs tested using non‐animal models including organ‐on‐a‐chip, organoid models or computational simulations will be allowed to continue to clinical trials subject to FDA approval.^[^ ^411^ ^]^ Conversely, there is a push for a reductionist approach to deconvolute biological systems to study single biomolecular interactions and elucidate new biological mechanisms.

In vitro models have so far been instrumental for understanding the complex interactions between the gut and its pathogens. As we look to the future there are several directions which would need to be realized for the enhancement of these models, they include:

- Physiological relevance: the structure and function of the intestine is intricately intertwined. Future models will need to improve upon the modelling of the in vivo architecture to mimic the function more closely – and vice versa.
- Incorporation of the microbiome: the presence of commensals and the absence of dysbiosis are important to the modulation of host‐pathogen interactions within the in vivo community. Static culture does not lend well to the incorporation of the microbiome component long term.
- Integration of immune components: interactions between pathogens and the host immune system is essential for modelling infection outcomes.
- Inclusion of related host tissues: to develop a more holistic view of host‐pathogen interactions it will be necessary to include other tissue types to study the systemic implications of intestinal infections and investigate crosstalk between organs and tissues. Body‐on‐a‐chip models will be useful in the future.
- Compatibility with advanced monitoring techniques: live monitoring of gut models that can accurately capture a time‐resolved dynamic response of the biological system – including barrier integrity, cell health, and protein expression, would vastly improve on the current models found in the literature.

The push for developing highly complex models, which employ real feats of engineering, unite the fields of biology, material science, engineering, and biotechnology. However, the usefulness of such models fundamentally depends on whether they are capable of answering the biological question the researcher is seeking to address.

## Conflict of Interest

The authors declare no conflict of interest.

## Author Contributions

R.M. and R.M.O. conceived and designed the review manuscript. R.M., S.O., W.J., K.W., and DH wrote the manuscript. R.M.O. supervised the authors and oversaw the project. D.B. and M.Z. supervised the authors and provided feedback. All authors approved the final manuscript.

## References

[1] A. Parker , M. A. E. Lawson , L. Vaux , C. Pin , Environ. Microbiol. 2018, 20, 2337. 28892253 10.1111/1462-2920.13926 PMC6175405

[2] A. K. DeGruttola , D. Low , A. Mizoguchi , E. Mizoguchi , Inflamm. Bowel Dis 2016, 22, 1137. 27070911 10.1097/MIB.0000000000000750 PMC4838534

[3] S. Carding , K. Verbeke , D. T. Vipond , B. M. Corfe , L. J. Owen , Microb. Ecol. Health Dis. 2015, 26, 26191. 25651997 10.3402/mehd.v26.26191 PMC4315779

[4] F. Ghiselli , B. Rossi , A. Piva , E. Grilli , Front. Vet. Sci. 2021, 8, 723387. 34888373 10.3389/fvets.2021.723387 PMC8649998

[5] R. Okumura , K. Takeda , Exp. Mol. Med. 2017, 49, 338. 10.1038/emm.2017.20 PMC5454438 28546564

[6] M. Bruewer , S. Samarin , A. Nusrat , Ann. N. Y. Acad. Sci. 2006, 1072, 242. 17057204 10.1196/annals.1326.017

[7] J. Mantaj , T. Abu‐Shams , Z. Enlo‐Scott , M. Swedrowska , D. Vllasaliu , Mol Pharm 2018, 15, 5802. 30380896 10.1021/acs.molpharmaceut.8b01053

[8] S. Tsukita , M. Furuse , M. Itoh , Nat. Rev. Mol. Cell Biol. 2001, 2, 285. 11283726 10.1038/35067088

[9] A. S. Yap , W. M. Brieher , B. M. Gumbiner , Annu. Rev. Cell Dev. Biol. 1997, 13, 119. 9442870 10.1146/annurev.cellbio.13.1.119

[10] L. J. Klunder , K. N. Faber , G. Dijkstra , S. C. D. Van Ijzendoorn , Cold Spring Harb Perspect. Biol. 2017, 9, a027888. 28213466 10.1101/cshperspect.a027888 PMC5495056

[11] S. W. Crawley , M. S. Mooseker , M. J. Tyska , J. Cell Biol. 2014, 207, 441. 25422372 10.1083/jcb.201407015 PMC4242837

[12] A. Ermund , A. Schütte , M. E. V. Johansson , J. K. Gustafsson , G. C. Hansson , Am. J. Physiol. 2013, 305, G341. 10.1152/ajpgi.00046.2013 PMC3761247 23832518

[13] G. C. Hansson , M. E. V. Johansson , Gut Microbes 2010, 1, 51. 21327117 10.4161/gmic.1.1.10470 PMC3035142

[14] R. Latorre , C. Sternini , R. De Giorgio , B. Greenwood‐Van Meerveld , Neurogastroenterol Motil 2016, 28, 620. 26691223 10.1111/nmo.12754 PMC4842178

[15] J. F. Rehfeld , Front. Endocrinol. (Lausanne) 2017, 8, 47. 28450850 10.3389/fendo.2017.00047 PMC5389988

[16] E. Karra , K. Chandarana , R. L. Batterham , J. Physiol. 2009, 587, 19. 19064614 10.1113/jphysiol.2008.164269 PMC2670018

[17] E. Deloose , W. Verbeure , I. Depoortere , J. Tack , Nat. Rev. Endocrinol. 2019, 15, 238. 30675023 10.1038/s41574-019-0155-0

[18] E. Marshman , C. Booth , C. S. Potten , BioEssays 2002, 24, 91. 11782954 10.1002/bies.10028

[19] N. Barker , M. Van De Wetering , H. Clevers , Genes Dev. 2008, 22, 1856. 18628392 10.1101/gad.1674008 PMC2735277

[20] R. Sancho , C. A. Cremona , A. Behrens , EMBO Rep. 2015, 16, 571. 25855643 10.15252/embr.201540188 PMC4428041

[21] E. S. Demitrack , L. C. Samuelson , J. Physiol. 2016, 594, 4791. 26848053 10.1113/JP271667 PMC5009795

[22] D. Pinto , A. Gregorieff , H. Begthel , H. Clevers , Genes Dev. 2003, 17, 1709. 12865297 10.1101/gad.267103 PMC196179

[23] V. Snoeck , B. Goddeeris , E. Cox , Microbes Infect. 2005, 7, 997. 15925533 10.1016/j.micinf.2005.04.003

[24] K. E. Boonekamp , T. L. Dayton , H. Clevers , J. Mol. Cell Biol. 2020, 12, 562. 32667995 10.1093/jmcb/mjaa034 PMC7683021

[25] K. Nakamura , Y. Yokoi , R. Fukaya , S. Ohira , R. Shinozaki , T. Nishida , M. Kikuchi , T. Ayabe , Front. Immunol. 2020, 11, 570296. 33154750 10.3389/fimmu.2020.570296 PMC7590646

[26] D. A. Elphick , Y. R. Mahida , Gut 2005, 54, 1802. 16284290 10.1136/gut.2005.068601 PMC1774800

[27] F. M. Gribble , F. Reimann , Nat. Rev. Endocrinol. 2019, 15, 226. 30760847 10.1038/s41574-019-0168-8

[28] C. Sternini , L. Anselmi , E. Rozengurt , Curr. Opin. Endocrinol. Diabetes Obes. 2008, 15, 73. 18185066 10.1097/MED.0b013e3282f43a73 PMC2943060

[29] Y. Yu , W. Yang , Y. Li , Y. Cong , Inflamm. Bowel Dis. 2020, 26, 11. 31560044 10.1093/ibd/izz217 PMC7539793

[30] S. K. Hendel , L. Kellermann , A. Hausmann , N. Bindslev , K. B. Jensen , O. H. Nielsen , Front Immunol 2022, 13, 822867. 35237268 10.3389/fimmu.2022.822867 PMC8884241

[31] S. Rajeev , O. Sosnowski , S. Li , T. Allain , A. G. Buret , D. M. McKay , Pathogens 2021, 10, 1163. 34578195 10.3390/pathogens10091163 PMC8467374

[32] J.‐P. Kraehenbuhl , M. R. Neutra , Annu. Rev. Cell Dev. Biol. 2000, 16, 301. 11031239 10.1146/annurev.cellbio.16.1.301

[33] C. S. Potten , M. Loeffler , Development 1990, 110, 1001. 2100251 10.1242/dev.110.4.1001

[34] J. I. Gordon , G. H. Schmidt , K. A. Roth , FASEB J. 1992, 6, 3039. 1521737 10.1096/fasebj.6.12.1521737

[35] J. L. K. Yip , G. K. Balasuriya , S. J. Spencer , E. L. Hill‐Yardin , Cell Mol. Gastroenterol. Hepatol. 2021, 12, 1701. 34506953 10.1016/j.jcmgh.2021.08.021 PMC8551786

[36] L. E. Smythies , M. Sellers , R. H. Clements , M. Mosteller‐Barnum , G. Meng , W. H. Benjamin , J. M. Orenstein , P. D. Smith , J. Clin. Invest. 2005, 115, 66. 15630445 10.1172/JCI19229 PMC539188

[37] H. Ma , W. Tao , S. Zhu , Cell Mol. Immunol. 2019, 16, 216. 30787416 10.1038/s41423-019-0208-2 PMC6460495

[38] F. Van Wijk , H. Cheroutre , Expert Rev. Clin. Immunol. 2010, 6, 559. 20594129 10.1586/eci.10.34 PMC2976609

[39] J. Spencer , L. M. Sollid , Mucosal Immunol. 2016, 9, 1113. 27461177 10.1038/mi.2016.59

[40] J. L. Coombes , F. Powrie , Nat. Rev. Immunol. 2008, 8, 435. 18500229 10.1038/nri2335 PMC2674208

[41] S. M. Jandhyala , R. Talukdar , C. Subramanyam , H. Vuyyuru , M. Sasikala , D. N. Reddy , World J. Gastroenterol. 2015, 21, 8836. 26269668 10.3748/wjg.v21.i29.8787 PMC4528021

[42] H. J. Wu , E. Wu , Gut Microbes 2012, 3, 4. 22356853 10.4161/gmic.19320 PMC3337124

[43] L. Lin , J. Zhang , BMC Immunol. 2017, 18, 2. 28061847 10.1186/s12865-016-0187-3 PMC5219689

[44] J. I. Park , S. W. Cho , J. H. Kang , T. E. Park , Tissue Eng. Regen. Med. 2023, 20, 341. 37079198 10.1007/s13770-023-00543-y PMC10117255

[45] D. Kaiserlian , K. Vidal , J.‐P. Revillard , Eur. J. Immunol. 1989, 19, 1513. 2570703 10.1002/eji.1830190827

[46] T. Ayabe , D. P. Satchell , C. L. Wilson , W. C. Parks , M. E. Selsted , A. J. Ouellette , Nat. Immunol. 2000, 1, 113. 11248802 10.1038/77783

[47] S. R. Lueschow , S. J. McElroy , Front. Immunol. 2020, 11, 520583. 10.3389/fimmu.2020.00587 PMC7145889 32308658

[48] A. Dupont , L. Heinbockel , K. Brandenburg , M. W. Hornef , Gut Microbes 2014, 5, 761. 25483327 10.4161/19490976.2014.972238 PMC4615892

[49] A. M. Mowat , W. W. Agace , Nat. Rev. Immunol. 2014, 14, 667. 25234148 10.1038/nri3738

[50] B. R. Glaysher , N. A. Mabbott , Immunology 2007, 120, 336. 17163957 10.1111/j.1365-2567.2006.02508.x PMC2265896

[51] M. R. Neutra , N. J. Mantis , J. P. Kraehenbuhl , Nat. Immunol. 2001, 2, 1004. 11685223 10.1038/ni1101-1004

[52] R. L. Owen , A. L. Jones , Gastroenterology 1974, 66, 189. 4810912

[53] C. Gutzeit , G. Magri , A. Cerutti , Immunol. Rev. 2014, 260, 76. 24942683 10.1111/imr.12189 PMC4174397

[54] A. Reboldi , J. G. Cyster , Immunol. Rev. 2016, 271, 230. 27088918 10.1111/imr.12400 PMC4835804

[55] G. Vighi , F. Marcucci , L. Sensi , G. Di Cara , F. Frati , Clin. Exp. Immunol. 2008, 153, 3. 18721321 10.1111/j.1365-2249.2008.03713.x PMC2515351

[56] I. M. Chiu , B. A. Heesters , N. Ghasemlou , C. A. Von Hehn , F. Zhao , J. Tran , B. Wainger , A. Strominger , S. Muralidharan , A. R. Horswill , J. B. Wardenburg , S. W. Hwang , M. C. Carroll , C. J. Woolf , Nature 2013, 501, 52. 23965627 10.1038/nature12479 PMC3773968

[57] I. M. Chiu , C. A. von Hehn , C. J. Woolf , Nat. Neurosci. 2012, 15, 1063. 22837035 10.1038/nn.3144 PMC3520068

[58] L. Edvinsson , R. Ekman , I. Jansen , J. McCulloch , R. Uddman , J. Cereb. Blood Flow Metab. 1987, 7, 720. 3500957 10.1038/jcbfm.1987.126

[59] J. C. Ansel , J. R. Brown , D. G. Payan , M. A. Brown , J. Immunol. 1993, 150, 4478. 7683320

[60] I. Raplee , L. Walker , L. Xu , A. Surathu , A. Chockalingam , S. Stewart , X. Han , R. Rouse , Z. Li , Antimicrob. Resist Infect. Control 2021, 10, 36. 33588951 10.1186/s13756-021-00903-0 PMC7885457

[61] S. Almagro‐Moreno , K. Pruss , R. K. Taylor , PLoS Pathog. 2015, 11, 1004787. 10.1371/journal.ppat.1004787 PMC4440752 25996593

[62] E. J. Nelson , J. B. Harris , J. Glenn Morris , S. B. Calderwood , A. Camilli , Nat. Rev. Microbiol. 2009, 7, 693. 19756008 10.1038/nrmicro2204 PMC3842031

[63] A. J. Silva , J. A. Benitez , PLoS Negl. Trop Dis. 2016, 10, 0004330. 10.1371/journal.pntd.0004330 PMC4741415 26845681

[64] T. Ramamurthy , R. K. Nandy , A. K. Mukhopadhyay , S. Dutta , A. Mutreja , K. Okamoto , S.‐I. Miyoshi , G. B. Nair , A. Ghosh , Front. Cell. Infect. Microbiol. 2020, 10, 572096. 33102256 10.3389/fcimb.2020.572096 PMC7554612

[65] A. Elmi , F. Nasher , N. Dorrell , B. Wren , O. Gundogdu , Front. Cell. Infect. Microbiol. 2021, 10, 607704. 33614526 10.3389/fcimb.2020.607704 PMC7887314

[66] M. E. Konkel , J. D. Klena , V. Rivera‐Amill , M. R. Monteville , D. Biswas , B. Raphael , J. Mickelson , J. Bacteriol. 2004, 186, 3296. 15150214 10.1128/JB.186.11.3296-3303.2004 PMC415756

[67] X. Cao , C. H. A. van de Lest , L. Z. X. Huang , J. P. M. van Putten , M. M. S. M. Wösten , Gut Microbes 2022, 14, 2091371. 35797141 10.1080/19490976.2022.2091371 PMC9272830

[68] M. Stahl , B. A. Vallance , Gut Microbes 2015, 6, 143. 25831043 10.1080/19490976.2015.1016691 PMC4615362

[69] K. Aktories , J. T. Barbieri , Nat. Rev. Microbiol. 2005, 3, 397. 15821726 10.1038/nrmicro1150

[70] G. Vedantam , A. Clark , M. Chu , R. McQuade , M. Mallozzi , V. K. Viswanathan , Gut Microbes 2012, 3, 121. 22555464 10.4161/gmic.19399 PMC3370945

[71] A. Rineh , M. J. Kelso , F. Vatansever , G. P. Tegos , M. R. Hamblin , Expert Rev Anti Infect Ther 2014, 12, 131. 24410618 10.1586/14787210.2014.866515 PMC4306399

[72] B. Pakbin , W. M. Brück , T. B. Brück , Int. J. Mol. Sci. 2023, 24, 2448. 36768771 10.3390/ijms24032448 PMC9917014

[73] P. Schnupf , P. J. Sansonetti , in Bacteria and Intracellularity, John Wiley & Sons, Inc., Hoboken, NJ, USA 2020, pp. 15–39.

[74] J. A. Vázquez‐Boland , M. Kuhn , P. Berche , T. Chakraborty , G. Domínguez‐Bernal , W. Goebel , B. González‐Zorn , J. Wehland , J. Kreft , Clin. Microbiol. Rev. 2001, 14, 584. 11432815 10.1128/CMR.14.3.584-640.2001 PMC88991

[75] J. J. Quereda , A. Morón‐García , C. Palacios‐Gorba , C. Dessaux , F. García‐del Portillo , M. G. Pucciarelli , A. D. Ortega , Virulence 2021, 12, 2509. 34612177 10.1080/21505594.2021.1975526 PMC8496543

[76] T. Sibanda , E. M. Buys , Microorganisms 2022, 10, 1522. 36013940 10.3390/microorganisms10081522 PMC9416357

[77] L. Radoshevich , P. Cossart , Nat. Rev. Microbiol. 2018, 16, 32. 29176582 10.1038/nrmicro.2017.126

[78] A. Fàbrega , J. Vila , Clin. Microbiol. Rev. 2013, 26, 308. 23554419 10.1128/CMR.00066-12 PMC3623383

[79] S.‐K. Eng , P. Pusparajah , N.‐S. Ab Mutalib , H.‐L. Ser , K.‐G. Chan , L.‐H. Lee , Front. Life Sci. 2015, 8, 284.

[80] K. Ahmad Bhat , T. Manzoor , M. Ahmad Dar , A. Farooq , K. Ahmad Allie , S. Majeed Wani , T. Ahmad Dar , A. Asghar Shah , in Enterobacteria, (Ed: S. B. Bhardwaj ), IntechOpen, Rijeka 2022, Ch. 3.

[81] S. Knutton , T. Baldwin , P. Williams , A. Manjarrez‐Hernandez , A. Aitken , Zentralbl Bakteriol 1993, 278, 209. 8347927 10.1016/s0934-8840(11)80838-8

[82] E. L. Hartland , J. M. Leong , Front. Cell. Infect. Microbiol. 2013, 3, 15. 23641365 10.3389/fcimb.2013.00015 PMC3639409

[83] M. O. Gaytán , V. I. Martínez‐Santos , E. Soto , B. González‐Pedrajo , Front. Cell. Infect. Microbiol. 2016, 6, 129. 27818950 10.3389/fcimb.2016.00129 PMC5073101

[84] A. R. Pacheco , V. Sperandio , Front. Cell. Infect. Microbiol. 2012, 2, 81. 22919672 10.3389/fcimb.2012.00081 PMC3417539

[85] M. B. Gonzalez‐Hernandez , T. Liu , H. C. Payne , J. E. Stencel‐Baerenwald , M. Ikizler , H. Yagita , T. S. Dermody , I. R. Williams , C. E. Wobus , J. Virol. 2014, 88, 6934. 24696493 10.1128/JVI.00204-14 PMC4054386

[86] K. Ettayebi , S. E. Crawford , K. Murakami , J. R. Broughman , U. Karandikar , V. R. Tenge , F. H. Neill , S. E. Blutt , X.‐L. Zeng , L. Qu , B. Kou , A. R. Opekun , D. Burrin , D. Y. Graham , S. Ramani , R. L. Atmar , M. K. Estes , Science 2016, 353, 1387. 27562956 10.1126/science.aaf5211 PMC5305121

[87] K. R. Grau , A. N. Roth , S. Zhu , A. Hernandez , N. Colliou , B. B. DiVita , D. T. Philip , C. Riffe , B. Giasson , S. M. Wallet , M. Mohamadzadeh , S. M. Karst , Nat. Microbiol. 2017, 2, 1586. 29109476 10.1038/s41564-017-0057-7 PMC5705318

[88] M. K. Jones , K. R. Grau , V. Costantini , A. O. Kolawole , M. de Graaf , P. Freiden , C. L. Graves , M. Koopmans , S. M. Wallet , S. A. Tibbetts , S. Schultz‐Cherry , C. E. Wobus , J. Vinjé , S. M. Karst , Nat. Protoc. 2015, 10, 1939. 26513671 10.1038/nprot.2015.121 PMC4689599

[89] S. M. Karst , C. E. Wobus , PLoS Pathog. 2015, 11, 1004626. 10.1371/journal.ppat.1004626 PMC4344369 25723501

[90] C. A. Omatola , A. O. Olaniran , Viruses 2022, 14, 875. 35632617 10.3390/v14050875 PMC9143449

[91] J. O. Amimo , S. A. Raev , J. Chepngeno , A. O. Mainga , Y. Guo , L. Saif , A. N. Vlasova , Front Immunol 2021, 12, 793841. 35003114 10.3389/fimmu.2021.793841 PMC8727603

[92] B. Li , S. Ding , N. Feng , N. Mooney , Y. S. Ooi , L. Ren , J. Diep , M. R. Kelly , L. L. Yasukawa , J. T. Patton , H. Yamazaki , T. Shirao , P. K. Jackson , H. B. Greenberg , Proc. Natl. Acad. Sci. USA 2017, 114, E3642. 28416666 10.1073/pnas.1619266114 PMC5422808

[93] B. J. Crenshaw , L. B. Jones , C. R. Bell , S. Kumar , Q. L. Matthews , Biomedicines 2019, 7, 61. 31430920 10.3390/biomedicines7030061 PMC6784011

[94] T. J. Wickham , P. Mathias , D. A. Cheresh , G. R. Nemerow , Cell 1993, 73, 309. 8477447 10.1016/0092-8674(93)90231-e

[95] V. Cortez , V. A. Meliopoulos , E. A. Karlsson , V. Hargest , C. Johnson , S. Schultz‐Cherry , Annu. Rev. Virol. 2017, 4, 327. 28715976 10.1146/annurev-virology-101416-041742

[96] L. A. Moser , M. Carter , S. Schultz‐Cherry , J. Virol. 2007, 81, 11937. 17699569 10.1128/JVI.00942-07 PMC2168760

[97] H. Ali , A. Lulla , A. S. Nicholson , J. Hankinson , E. B. Wignall‐Fleming , R. L. O'Connor , D.‐L. Vu , S. C. Graham , J. E. Deane , S. Guix , V. Lulla , PLoS Biol. 2023, 21, 3001815. 10.1371/journal.pbio.3001815 PMC10374088 37459343

[98] G. Lanave , D. Loconsole , F. Centrone , C. Catella , P. Capozza , G. Diakoudi , A. Parisi , E. Suffredini , A. Buonavoglia , M. Camero , M. Chironna , V. Martella , Transbound Emerg. Dis. 2022, 69, 864. 33411943 10.1111/tbed.13979

[99] C. Johnson , V. Hargest , V. Cortez , V. Meliopoulos , S. Schultz‐Cherry , Viruses 2017, 9, 22. 28117758 10.3390/v9010022 PMC5294991

[100] C. J. Burrell , C. R. Howard , F. A. Murphy , Fenner and White's Medical Virology, Elsevier, Amsterdam 2017, pp. 473–476.

[101] S. Schultz‐Cherry , Reference Module in Biomedical Sciences, Elsevier, Amsterdam 2014.

[102] V. Cortez , D. F. Boyd , J. C. Crawford , B. Sharp , B. Livingston , H. M. Rowe , A. Davis , R. Alsallaq , C. G. Robinson , P. Vogel , J. W. Rosch , E. Margolis , P. G. Thomas , S. Schultz‐Cherry , Nat. Commun. 2020, 11, 2097. 32350281 10.1038/s41467-020-15999-y PMC7190700

[103] Z. Khreefa , M. T. Barbier , A. R. Koksal , G. Love , L. Del Valle , Cells 2023, 12, 262. 36672197 10.3390/cells12020262 PMC9856332

[104] R. Zang , M. F. G. Castro , B. T. McCune , Q. Zeng , P. W. Rothlauf , N. M. Sonnek , Z. Liu , K. F. Brulois , X. Wang , H. B. Greenberg , M. S. Diamond , M. A. Ciorba , S. P. J. Whelan , S. Ding , Sci. Immunol. 2020, 5, abc3582. 10.1126/sciimmunol.abc3582 PMC7285829 32404436

[105] J. J. Lee , S. Kopetz , E. Vilar , J. P. Shen , K. Chen , A. Maitra , Genes (Basel) 2020, 11, 645. 32545271 10.3390/genes11060645 PMC7349178

[106] S. S. K. Durairajan , A. K. Singh , U. B. Saravanan , M. Namachivayam , M. Radhakrishnan , J.‐D. Huang , R. Dhodapkar , H. Zhang , Viruses 2023, 15, 1231. 37376531 10.3390/v15061231 PMC10304713

[107] H. Zhang , B. Shao , Q. Dang , Z. Chen , Q. Zhou , H. Luo , W. Yuan , Z. Sun , Front. Immunol., 2021, 12, 674074. 34858386 10.3389/fimmu.2021.674074 PMC8631495

[108] S. Jawhara , Microorganisms 2022, 10, 1014. 35630457 10.3390/microorganisms10051014 PMC9147621

[109] F. L. Mayer , D. Wilson , B. Hube , Virulence 2013, 4, 119. 23302789 10.4161/viru.22913 PMC3654610

[110] J. P. Lopes , M. S. Lionakis , Virulence 2022, 13, 89. 34964702 10.1080/21505594.2021.2019950 PMC9728475

[111] R. Alonso‐Monge , M. S. Gresnigt , E. Román , B. Hube , J. Pla , PLoS Pathog. 2021, 17, 1009710. 10.1371/journal.ppat.1009710 PMC8297749 34293071

[112] J. H. Sung , Y. I. Wang , N. Narasimhan Sriram , M. Jackson , C. Long , J. J. Hickman , M. L. Shuler , Anal. Chem., 2019, 91, 330. 30472828 10.1021/acs.analchem.8b05293 PMC6687466

[113] Z. A. Li , R. S. Tuan , Stem Cell Res Ther 2022, 13, 431 35987699 10.1186/s13287-022-03130-5 PMC9392934

[114] S. A. Tria , M. Ramuz , M. Huerta , P. Leleux , J. Rivnay , L. H. Jimison , A. Hama , G. G. Malliaras , R. M. Owens , Adv. Healthcare Mater. 2014, 3, 1053. 10.1002/adhm.201300632 24497469

[115] N. M. Negretti , G. Clair , P. K. Talukdar , C. R. Gourley , S. Huynh , J. N. Adkins , C. T. Parker , C. M. Corneau , M. E. Konkel , Front. Microbiol. 2019, 10, 755. 31031730 10.3389/fmicb.2019.00755 PMC6470190

[116] A. Kumar , Y. A. Helmy , Z. Fritts , A. Vlasova , L. J. Saif , G. Rajashekara , Probiotics Antimicrob. Proteins 2023, 15, 107. 35034323 10.1007/s12602-021-09884-3

[117] C. Schimpel , C. Passegger , S. Egger , C. Tam‐Amersdorfer , H. Strobl , Eur. J. Immunol. 2023, 53, 2250131. 10.1002/eji.202250131 36527196

[118] F. Kesisoglou , P. Schmiedlin‐Ren , D. Fleisher , E. M. Zimmermann , Mol. Pharm. 2010, 7, 619. 20235596 10.1021/mp9001377 PMC2882510

[119] M. García‐Díaz , M. del MM Cendra , R. Alonso‐Roman , M. Urdániz , E. Torrents , E. Martínez , Pharmaceutics 2022, 14, 1552. 35893808 10.3390/pharmaceutics14081552 PMC9331835

[120] Y. Chen , S. E. Rudolph , B. N. Longo , F. Pace , T. T. Roh , R. Condruti , M. Gee , P. I. Watnick , D. L. Kaplan , Adv. Healthcare Mater. 2022, 11, 2200447. 10.1002/adhm.202200447 PMC9388577 35686484

[121] J. L. Leslie , S. Huang , J. S. Opp , M. S. Nagy , M. Kobayashi , V. B. Young , J. R. Spence , Infect. Immun. 2015, 83, 138. 25312952 10.1128/IAI.02561-14 PMC4288864

[122] M. M. Lamers , J. Beumer , J. van der Vaart , K. Knoops , J. Puschhof , T. I. Breugem , R. B. G. Ravelli , J. Paul van Schayck , A. Z. Mykytyn , H. Q. Duimel , E. van Donselaar , S. Riesebosch , H. J. H. Kuijpers , D. Schipper , W. J. van de Wetering , M. de Graaf , M. Koopmans , E. Cuppen , P. J. Peters , B. L. Haagmans , H. Clevers , Science 2020, 369, 50. 32358202 10.1126/science.abc1669 PMC7199907

[123] C. Biel , O. Martinec , B. Sibering , K. van Summeren , A. M. A. Wessels , D. J. Touw , K. P. de Jong , V. E. de Meijer , K. N. Faber , J. P. ten Klooster , I. A. M. de Graaf , P. Olinga , Arch. Toxicol. 2022, 96, 1815. 35428896 10.1007/s00204-022-03295-1 PMC9095520

[124] M. Nikolaev , O. Mitrofanova , N. Broguiere , S. Geraldo , D. Dutta , Y. Tabata , B. Elci , N. Brandenberg , I. Kolotuev , N. Gjorevski , H. Clevers , M. P. Lutolf , Nature 2020, 585, 574. 32939089 10.1038/s41586-020-2724-8

[125] F. S. Gazzaniga , D. M. Camacho , M. Wu , M. F. Silva Palazzo , A. L. M. Dinis , F. N. Grafton , M. J. Cartwright , M. Super , D. L. Kasper , D. E. Ingber , Front. Cell Infect. Microbiol. 2021, 11, 638014. 33777849 10.3389/fcimb.2021.638014 PMC7996096

[126] T. N. Tun , P. J. Cameron , A. T. A. Jenkins , Biosens. Bioelectron. 2011, 28, 227. 21835605 10.1016/j.bios.2011.07.023

[127] J. Takagi , K. Aoki , B. S. Turner , S. Lamont , S. Lehoux , N. Kavanaugh , M. Gulati , A. Valle Arevalo , T. J. Lawrence , C. Y. Kim , B. Bakshi , M. Ishihara , C. J. Nobile , R. D. Cummings , D. J. Wozniak , M. Tiemeyer , R. Hevey , K. Ribbeck , Nat. Chem. Biol. 2022, 18, 762. 35668191 10.1038/s41589-022-01035-1 PMC7613833

[128] B. X. Wang , J. Takagi , A. McShane , J. H. Park , K. Aoki , C. Griffin , J. Teschler , G. Kitts , G. Minzer , M. Tiemeyer , R. Hevey , F. Yildiz , K. Ribbeck , EMBO J. 2023, 42, 111562. 10.15252/embj.2022111562 PMC9890226 36504455

[129] T. Lea , in The Impact of Food Bioactives on Health, (Eds: K. Verhoeckx , P. Cotter , I. López‐Expósito , C. Kleiveland , T. Lea , A. Mackie , T. Requena , D. Swiatecka , H. Wichers ), Springer International Publishing, Cham 2015, pp. 103–111. 29787039

[130] M. J. Briske‐Anderson , J. W. Finley , S. M. Newman , Exp. Biol. Med. 1997, 214, 248. 10.3181/00379727-214-44093 9083258

[131] O. Anna , L. Monika , G. Włodzimierz , C. Katarzyna , Pol J. Food Nutr. Sci. 2003, 53, 60.

[132] N. Langerak , H. M. M. Ahmed , Y. Li , I. R. Middel , H. Eslami Amirabadi , J. Malda , R. Masereeuw , R. van Roij , Front. Bioeng. Biotechnol. 2020, 8, 763. 32793567 10.3389/fbioe.2020.00763 PMC7393935

[133] R. Singh , A. A. Fouladi‐Nashta , D. Li , N. Halliday , D. A. Barrett , K. D. Sinclair , J. Cell. Biochem. 2006, 99, 146. 16598758 10.1002/jcb.20908

[134] D. Martínez‐Maqueda , B. Miralles , I. Recio , in The Impact of Food Bioactives on Health, Springer International Publishing, Cham 2015, pp. 113–124.

[135] S. Devriese , L. Van den Bossche , S. Van Welden , T. Holvoet , I. Pinheiro , P. Hindryckx , M. De Vos , D. Laukens , Histochem. Cell Biol. 2017, 148, 85. 28265783 10.1007/s00418-017-1539-7

[136] A. Dillon , D. D. Lo , Front Immunol 2019, 10, 1499. 31312204 10.3389/fimmu.2019.01499 PMC6614372

[137] S. Kernéis , E. Caliot , H. Stubbe , A. Bogdanova , J.‐P. Kraehenbuhl , E. Pringault , Microbes Infect 2000, 2, 1119. 10967292 10.1016/s1286-4579(00)01266-1

[138] F. Antunes , F. Andrade , F. Araújo , D. Ferreira , B. Sarmento , Eur. J. Pharm. Biopharm. 2013, 83, 427. 23159710 10.1016/j.ejpb.2012.10.003

[139] I. Lozoya‐Agullo , F. Araújo , I. González‐Álvarez , M. Merino‐Sanjuán , M. González‐Álvarez , M. Bermejo , B. Sarmento , Mol Pharm 2017, 14, 1264. 28263609 10.1021/acs.molpharmaceut.6b01165

[140] E. Gullberg , M. Leonard , J. Karlsson , A. M. Hopkins , D. Brayden , A. W. Baird , P. Artursson , Biochem. Biophys. Res. Commun. 2000, 279, 808. 11162433 10.1006/bbrc.2000.4038

[141] A. A. M. Kämpfer , P. Urbán , S. Gioria , N. Kanase , V. Stone , A. Kinsner‐Ovaskainen , Toxicol. In Vitro 2017, 45, 31. 28807632 10.1016/j.tiv.2017.08.011 PMC5744654

[142] N. K. Kordulewska , J. Topa , M. Tańska , A. Cieślińska , E. Fiedorowicz , H. F. J. Savelkoul , B. Jarmołowska , Nutrients 2021, 13, 123. 10.3390/nu13010123 PMC7824174 33396265

[143] C. M. Moysidou , A. M. Withers , A. J. Nisbet , D. R. G. Price , C. E. Bryant , C. Cantacessi , R. M. Owens , Adv Biol 2022, 6, 2200015. 10.1002/adbi.202200015 35652159

[144] A. Vasiee , F. Falah , B. A. Behbahani , F. Tabatabaee‐yazdi , J. Biosci. Bioeng. 2020, 130, 471. 32753308 10.1016/j.jbiosc.2020.07.002

[145] X. Ding , X. Hu , Y. Chen , J. Xie , M. Ying , Y. Wang , Q. Yu , Trends Food Sci. Technol. 2021, 107, 455.

[146] P. Hoffmann , M. Burmester , M. Langeheine , R. Brehm , M. T. Empl , B. Seeger , G. Breves , PLoS One 2021, 16, 0257824. 10.1371/journal.pone.0257824 PMC8496855 34618824

[147] J. Cao , X. Wu , X. Qin , Z. Li , J. Proteome Res. 2021, 20, 1582. 33555889 10.1021/acs.jproteome.0c00806

[148] K. Verhoeckx , P. Cotter , I. López‐Expósito , C. Kleiveland , T. Lea , A. Mackie , T. Requena , D. Swiatecka , H. Wichers , in The Impact of Food Bioactives on Health, (Eds: K. Verhoeckx , P. Cotter , I. López‐Expósito , C. Kleiveland , T. Lea , A. Mackie ; T. Requena ; D. Swiatecka , H. Wichers ), Springer International Publishing, Cham 2015, p. 338. 29787039

[149] R. Dahiya , T. Lesuffleur , K. S. Kwak , J. C. Byrd , A. Barbat , A. Zweibaum , Y. S. Kim , Cancer Res. 1992, 52, 4655. 1511431

[150] B. McCormick , Curr. Opin. Microbiol. 2003, 6, 77. 12615224 10.1016/s1369-5274(02)00003-6

[151] J. L. Madara , J. Stafford , K. Dharmsathaphorn , S. Carlson , Gastroenterology 1987, 92, 1133. 3557010 10.1016/s0016-5085(87)91069-9

[152] D. J. McCool , M. A. Marcon , J. F. Forstner , G. G. Forstner , Biochem. J. 1990, 267, 491. 2110452 10.1042/bj2670491 PMC1131316

[153] Y.‐N. B. Biazik , J. M. Jahn , K. A. Su , Y. Wu , Filip , World J. Gastroenterol. 2010, 16, 2743. 20533594 10.3748/wjg.v16.i22.2743 PMC2883130

[154] J. Camps , K. Mrasek , E. Prat , A. Weise , H. Starke , J. Egozcue , R. Miró , T. Liehr , Oncol. Rep. 2004, 11, 1215. 15138558

[155] F.‐C. Huang , Gut Pathog. 2016, 8, 5. 26893616 10.1186/s13099-016-0088-2 PMC4758167

[156] C. J. Hunter , V. K. Singamsetty , N. K. Chokshi , P. Boyle , V. Camerini , A. V. Grishin , J. S. Upperman , H. R. Ford , N. V. Prasadarao , J Infect Dis 2008, 198, 586. 18588483 10.1086/590186 PMC2497445

[157] M. K. Puthia , S. W. S. Sio , J. Lu , K. S. W. Tan , Infect. Immun. 2006, 74, 4114. 16790785 10.1128/IAI.00328-06 PMC1489721

[158] R. E. McCabe , G. S. M. Yu , C. Conteas , P. R. Morrill , B. McMorrow , Antimicrob. Agents Chemother. 1991, 35, 29. 1901700 10.1128/aac.35.1.29 PMC244937

[159] A. Barbat , I. Pandrea , D. Cambier , A. Zweibaum , T. Lesuffleur , Int. J. Cancer 1998, 75, 731. 9495241 10.1002/(sici)1097-0215(19980302)75:5<731::aid-ijc11>3.0.co;2-9

[160] C. Alcantara Warren , R. V. Destura , J. E. A. D. Sevilleja , L. F. Barroso , H. Carvalho , L. J. Barrett , A. D. O'Brien , R. L. Guerrant , J. Infect. Dis. 2008, 198, 143. 18498239 10.1086/588819 PMC2631281

[161] M. A. Panaro , A. Cianciulli , V. Mitolo , C. I. Mitolo , A. Acquafredda , O. Brandonisio , P. Cavallo , FEMS Immunol. Med. Microbiol. 2007, 51, 302. 17714487 10.1111/j.1574-695X.2007.00304.x

[162] D. B. Huang , H. L. DuPont , Z.‐D. Jiang , L. Carlin , P. C. Okhuysen , Clin. Vaccine Immunol. 2004, 11, 548. 10.1128/CDLI.11.3.548-551.2004 PMC404585 15138180

[163] K. Souček , P. Gajdušková , M. Brázdová , M. Hýžd'alová , L. Kočí , D. Vydra , R. Trojanec , Z. Pernicová , L. Lentvorská , M. Hajdúch , J. Hofmanová , A. Kozubík , Cancer Genet. Cytogenet. 2010, 197, 107. 20193843 10.1016/j.cancergencyto.2009.11.009

[164] P. Cao , Y. Chen , X. Guo , Y. Chen , W. Su , N. Zhan , W. Dong , Front. Pharmacol. 2020, 11, 565644. 32153411 10.3389/fphar.2020.00106 PMC7047714

[165] M. B. Karpova , J. Schoumans , I. Ernberg , J.‐I. Henter , M. Nordenskjöld , B. Fadeel , Leukemia 2005, 19, 159. 15457187 10.1038/sj.leu.2403534

[166] K. Masuda , A. Kajikawa , S. Igimi , Biosci. Microflora 2011, 30, 37. 25045312 10.12938/bifidus.30.37 PMC4103634

[167] H. Bosshart , M. Heinzelmann , Ann. Transl. Med. 2016, 4, 438. 27942529 10.21037/atm.2016.08.53 PMC5124613

[168] H. Satsu , Y. Ishimoto , T. Nakano , T. Mochizuki , T. Iwanaga , M. Shimizu , Exp. Cell Res. 2006, 312, 3909. 17010338 10.1016/j.yexcr.2006.08.018

[169] P. B. Aldo , V. Craveiro , S. Guller , G. Mor , Am. J. Reprod. Immunol. 2013, 70, 80. 23621670 10.1111/aji.12129 PMC3703650

[170] B. Kaeffer , In Vitro Cell. Dev. Biol.: Anim. 2002, 38, 123. 12026159 10.1290/1071-2690(2002)038<0123:MIECIP>2.0.CO;2

[171] S. Frisch , H. Francis , J. Cell Biol. 1994, 124, 619. 8106557 10.1083/jcb.124.4.619 PMC2119917

[172] J. S. Dutton , S. S. Hinman , R. Kim , Y. Wang , N. L. Allbritton , Trends Biotechnol. 2019, 37, 744. 30591184 10.1016/j.tibtech.2018.12.001 PMC6571163

[173] C. Moon , K. L. Vandussen , H. Miyoshi , T. S. Stappenbeck , Mucosal Immunol. 2014, 7, 818. 24220295 10.1038/mi.2013.98 PMC4019725

[174] B. van der Hee , L. M. P. Loonen , N. Taverne , J. J. Taverne‐Thiele , H. Smidt , J. M. Wells , Stem Cell Res. 2018, 28, 165. 29499500 10.1016/j.scr.2018.02.013

[175] K. Kozuka , Y. He , S. Koo‐McCoy , P. Kumaraswamy , B. Nie , K. Shaw , P. Chan , M. Leadbetter , L. He , J. G. Lewis , Z. Zhong , D. Charmot , M. Balaa , A. J. King , J. S. Caldwell , M. Siegel , Stem Cell Rep. 2017, 9, 1976. 10.1016/j.stemcr.2017.10.013 PMC5785676 29153987

[176] T. Sato , R. G. Vries , H. J. Snippert , M. van de Wetering , N. Barker , D. E. Stange , J. H. van Es , A. Abo , P. Kujala , P. J. Peters , H. Clevers , Nature 2009, 459, 262. 19329995 10.1038/nature07935

[177] L. Hayflick , P. S. Moorhead , Exp. Cell Res. 1961, 25, 585. 13905658 10.1016/0014-4827(61)90192-6

[178] M. Fujii , M. Matano , K. Toshimitsu , A. Takano , Y. Mikami , S. Nishikori , S. Sugimoto , T. Sato , Cell Stem Cell 2018, 23, 787. 30526881 10.1016/j.stem.2018.11.016

[179] S. Beyaz , M. D. Mana , J. Roper , D. Kedrin , A. Saadatpour , S. J. Hong , K. E. Bauer‐Rowe , M. E. Xifaras , A. Akkad , E. Arias , L. Pinello , Y. Katz , S. Shinagare , M. Abu‐Remaileh , M. M. Mihaylova , D. W. Lamming , R. Dogum , G. Guo , G. W. Bell , M. Selig , G. P. Nielsen , N. Gupta , C. R. Ferrone , V. Deshpande , G. C. Yuan , S. H. Orkin , D. M. Sabatini , Ö. H. Yilmaz , Nature 2016, 531, 53. 26935695 10.1038/nature17173 PMC4846772

[180] S. Sugimoto , E. Kobayashi , M. Fujii , Y. Ohta , K. Arai , M. Matano , K. Ishikawa , K. Miyamoto , K. Toshimitsu , S. Takahashi , K. Nanki , Y. Hakamata , T. Kanai , T. Sato , Nature 2021, 592, 99. 33627870 10.1038/s41586-021-03247-2

[181] T. Zietek , P. Giesbertz , M. Ewers , F. Reichart , M. Weinmüller , E. Urbauer , D. Haller , I. E. Demir , G. O. Ceyhan , H. Kessler , E. Rath , Front Bioeng Biotechnol 2020, 8, 577656. 33015026 10.3389/fbioe.2020.577656 PMC7516017

[182] N. Barker , J. H. Van Es , J. Kuipers , P. Kujala , M. Van Den Born , M. Cozijnsen , A. Haegebarth , J. Korving , H. Begthel , P. J. Peters , H. Clevers , Nature 2007, 449, 1003. 17934449 10.1038/nature06196

[183] J. Foulke‐Abel , J. In , O. Kovbasnjuk , N. C. Zachos , K. Ettayebi , S. E. Blutt , J. M. Hyser , X. L. Zeng , S. E. Crawford , J. R. Broughman , M. K. Estes , M. Donowitz , Exp. Biol. Med. 2014, 239, 1124. 10.1177/1535370214529398 PMC4380516 24719375

[184] S. Middendorp , K. Schneeberger , C. L. Wiegerinck , M. Mokry , R. D. L. Akkerman , S. Van Wijngaarden , H. Clevers , E. E. S. Nieuwenhuis , Stem Cells 2014, 32, 1083. 24496776 10.1002/stem.1655

[185] Y. Wang , M. DiSalvo , D. B. Gunasekara , J. Dutton , A. Proctor , M. S. Lebhar , I. A. Williamson , J. Speer , R. L. Howard , N. M. Smiddy , S. J. Bultman , C. E. Sims , S. T. Magness , N. L. Allbritton , Cell. Mol. Gastroenterol. Hepatol. 2017, 4, 165. 29204504 10.1016/j.jcmgh.2017.02.011 PMC5710741

[186] R. F. Ramig , J. Virol. 2004, 78, 10213. 15367586 10.1128/JVI.78.19.10213-10220.2004 PMC516399

[187] S. R. Finkbeiner , X. L. Zeng , B. Utama , R. L. Atmar , N. F. Shroyer , M. K. Estesa , mBio 2012, 3, 10. 10.1128/mBio.00159-12 PMC3398537 22761392

[188] B. J. Koestler , C. M. Ward , C. R. Fisher , A. Rajan , A. W. Maresso , S. M. Payne , Infect. Immun. 2019, 87, 10. 10.1128/IAI.00733-18 PMC6434139 30642906

[189] L. J. Klunder , K. N. Faber , G. Dijkstra , S. C. D. Van Ijzendoorn , Cold Spring Harbor Perspect. Biol. 2017, 9, a027888. 10.1101/cshperspect.a027888 PMC5495056 28213466

[190] E. J. Irvine , J. K. Marshall , Gastroenterology 2000, 119, 1740. 11113095 10.1053/gast.2000.20231

[191] J. Lee , J. H. Mo , K. Katakura , I. Alkalay , A. N. Rucker , Y. T. Liu , H. K. Lee , C. Shen , G. Cojocaru , S. Shenouda , M. Kagnoff , L. Eckmann , Y. Ben‐Neriah , E. Raz , Nat. Cell Biol. 2006, 8, 1327. 17128265 10.1038/ncb1500

[192] J. Liu , J. Li , Y. Ren , P. Liu , Int. J. Biol. Sci. 2014, 10, 543. 24910533 10.7150/ijbs.8888 PMC4046881

[193] S. Xu , F. Zhou , J. Tao , L. Song , N. G. Siew Chien , X. Wang , L. Chen , F. Yi , Z. Ran , R. Zhou , B. Xia , PLoS One 2014, 9, 99807. 10.1371/journal.pone.0099807 PMC4061034 24937328

[194] A. E. Vickers , V. Fischer , S. Connors , R. L. Fisher , J. P. Baldeck , G. Maurer , K. Brendel , Drug Metab Dispos 1992, 20, 802. 1362930

[195] H. Q. Chen , J. Yang , M. Zhang , Y. K. Zhou , T. Y. Shen , Z. X. Chu , M. Zhang , X. M. Hang , Y. Q. Jiang , H. L. Qin , Am. J. Physiol. 2010, 299, 1287. 10.1152/ajpgi.00196.2010 20884889

[196] S. M. N. Udden , S. Waliullah , M. Harris , H. Zaki , J Vis Exp 2017, 2017, 55347. 10.3791/55347 PMC5408962 28287576

[197] I. A. M. De Graaf , P. Olinga , M. H. De Jager , M. T. Merema , R. De Kanter , E. G. Van De Kerkhof , G. M. M. Groothuis , Nat. Protoc. 2010, 5, 1540. 20725069 10.1038/nprot.2010.111

[198] E. G. Van De Kerkhof , I. A. M. De Graaf , M. H. De Jager , D. K. F. Meijer , G. M. M. Groothuis , Drug Metab. Dispos. 2005, 33, 1613. 16051733 10.1124/dmd.105.005686

[199] L. A. Schwerdtfeger , E. P. Ryan , S. A. Tobet , Am. J. Physiol. 2016, 310, G240. 10.1152/ajpgi.00299.2015 PMC4754739 26680736

[200] R. Bartolí , J. Boix , G. Òdena , N. D. D. la Ossa , V. M. de Vega , V. Lorenzo‐Zúñiga , World J. Gastrointest. Endosc. 2013, 5, 226. 23678375 10.4253/wjge.v5.i5.226 PMC3653021

[201] E. G. Van De Kerkhof , I. A. M. De Graaf , M. H. De Jager , G. M. M. Groothuis , Drug Metab. Dispos. 2007, 35, 898. 17344336 10.1124/dmd.106.014563

[202] M. Donowitz , G. T. Keusch , H. J. Binder , Gastroenterology 1975, 69, 1230. 172398

[203] C. Z. Shi , H. Q. Chen , Y. Liang , Y. Xia , Y. Z. Yang , J. Yang , J. D. Zhang , S. H. Wang , J. Liu , H. L. Qin , World J. Gastroenterol. 2014, 20, 4636. 24782616 10.3748/wjg.v20.i16.4636 PMC4000500

[204] R. De Kanter , A. Tuin , E. Van De Kerkhof , M. Martignoni , A. L. Draaisma , M. H. De Jager , I. A. M. De Graaf , D. K. F. Meijer , G. M. M. Groothuis , J. Pharmacol. Toxicol. Methods 2005, 51, 65. 15596116 10.1016/j.vascn.2004.07.007

[205] C. L. Krumdieck , J. dos Santos , K. J. Ho , Anal. Biochem. 1980, 104, 118. 6770714 10.1016/0003-2697(80)90284-5

[206] M. Baydoun , A. Treizeibré , J. Follet , S. B. Vanneste , C. Creusy , L. Dercourt , B. Delaire , A. Mouray , E. Viscogliosi , G. Certad , V. Senez , Micromachines 2020, 11, 150. 32019215 10.3390/mi11020150 PMC7074597

[207] M. Li , I. A. De Graaf , G. M. Groothuis , Expert Opin. Drug Metab. Toxicol. 2016, 12, 175. 26750630 10.1517/17425255.2016.1125882

[208] R. De Kanter , M. H. De Jager , A. L. Draaisma , J. U. Jurva , P. Olinga , D. K. F. Meijer , G. M. M. Groothuis , Xenobiotica 2002, 32, 349. 12065058 10.1080/00498250110112006

[209] D. Punyadarsaniya , C. Winter , A. K. Mork , M. Amiri , H. Y. Naim , S. Rautenschlein , G. Herrler , J. Virol. Methods 2015, 212, 71. 25445801 10.1016/j.jviromet.2014.10.015 PMC7172049

[210] J. K. Gustafsson , A. Ermund , M. E. V. Johansson , A. Schütte , G. C. Hansson , H. Sjövall , Am. J. Physiol. 2012, 302, G430. 10.1152/ajpgi.00405.2011 PMC4073982 22159279

[211] K. Tsilingiri , A. Sonzogni , F. Caprioli , M. Rescigno , J Vis Exp 2013, 1, 4368. 10.3791/4368 PMC3667689 23666550

[212] J. C. Fontoura , C. Viezzer , F. G. dos Santos , R. A. Ligabue , R. Weinlich , R. D. Puga , D. Antonow , P. Severino , C. Bonorino , Mater. Sci. Eng., C 2020, 107, 110264. 10.1016/j.msec.2019.110264 31761183

[213] S. M. Jung , S. Kim , Front. Microbiol. 2022, 12, 767038. 35058894 10.3389/fmicb.2021.767038 PMC8765704

[214] K. P. Nickerson , A. Llanos‐Chea , L. Ingano , G. Serena , A. Miranda‐Ribera , M. Perlman , R. Lima , M. B. Sztein , A. Fasano , S. Senger , C. S. Faherty , Microbiol. Spectr. 2021, 9, 00003. 10.1128/spectrum.00003-21 PMC8552518 34106568

[215] K. L. VanDussen , J. M. Marinshaw , N. Shaikh , H. Miyoshi , C. Moon , P. I. Tarr , M. A. Ciorba , T. S. Stappenbeck , Gut 2015, 64, 911. 25007816 10.1136/gutjnl-2013-306651 PMC4305344

[216] M. Kasendra , A. Tovaglieri , A. Sontheimer‐Phelps , S. Jalili‐Firoozinezhad , A. Bein , A. Chalkiadaki , W. Scholl , C. Zhang , H. Rickner , C. A. Richmond , H. Li , D. T. Breault , D. E. Ingber , Sci. Rep. 2018, 8, 2871. 29440725 10.1038/s41598-018-21201-7 PMC5811607

[217] C. S. Park , L. P. Nguyen , D. Yong , Cells 2020, 9, 2209. 33003541 10.3390/cells9102209 PMC7600593

[218] N. Hammoudi , S. Hamoudi , J. Bonnereau , H. Bottois , K. Pérez , M. Bezault , D. Hassid , V. Chardiny , C. Grand , B. Gergaud , J. Bonnet , L. Chedouba , M. L. Tran Minh , J. M. Gornet , C. Baudry , H. Corte , L. Maggiori , A. Toubert , J. McBride , C. Brochier , M. Neighbors , L. Le Bourhis , M. Allez , Front Immunol 2022, 13, 1008456. 36439157 10.3389/fimmu.2022.1008456 PMC9685428

[219] A. Pastuła , M. Middelhoff , A. Brandtner , M. Tobiasch , B. Höhl , A. H. Nuber , I. E. Demir , S. Neupert , P. Kollmann , G. Mazzuoli‐Weber , M. Quante , Stem Cells Int 2016, 2016, 3710836. 26697073 10.1155/2016/3710836 PMC4677245

[220] R. Feng , E. Aihara , S. Kenny , L. Yang , J. Li , A. Varro , M. H. Montrose , N. F. Shroyer , T. C. Wang , R. A. Shivdasani , Y. Zavros , Gastroenterology 2014, 147, 655. 24859162 10.1053/j.gastro.2014.05.006 PMC4211430

[221] L. Kandilogiannakis , E. Filidou , I. Drygiannakis , G. Tarapatzi , S. Didaskalou , M. Koffa , K. Arvanitidis , G. Bamias , V. Valatas , V. Paspaliaris , G. Kolios , Stem Cells Int 2021, 2021, 9929461. 34354753 10.1155/2021/9929461 PMC8331310

[222] N. Lahar , N. Y. Lei , J. Wang , Z. Jabaji , S. C. Tung , V. Joshi , M. Lewis , M. Stelzner , M. G. Martín , J. C. Y. Dunn , PLoS One 2011, 6, 26898. 10.1371/journal.pone.0026898 PMC3219641 22125602

[223] M. Meir , N. Burkard , H. Ungewiß , M. Diefenbacher , S. Flemming , F. Kannapin , C. T. Germer , M. Schweinlin , M. Metzger , J. Waschke , N. Schlegel , J. Clin. Invest. 2019, 129, 2824. 31205031 10.1172/JCI120261 PMC6597228

[224] G. Altay , E. Larrañaga , S. Tosi , F. M. Barriga , E. Batlle , V. Fernández‐Majada , E. Martínez , Sci. Rep. 2019, 9. 10.1038/s41598-019-55181-z PMC6895096 31806863

[225] P. Schlaermann , B. Toelle , H. Berger , S. C. Schmidt , M. Glanemann , J. Ordemann , S. Bartfeld , H. J. Mollenkopf , T. F. Meyer , Gut 2016, 65, 202. 25539675 10.1136/gutjnl-2014-307949 PMC4752654

[226] L. Albenberg , T. V. Esipova , C. P. Judge , K. Bittinger , J. Chen , A. Laughlin , S. Grunberg , R. N. Baldassano , J. D. Lewis , H. Li , S. R. Thom , F. D. Bushman , S. A. Vinogradov , G. D. Wu , Gastroenterology 2014, 147, 1055. 25046162 10.1053/j.gastro.2014.07.020 PMC4252572

[227] R. Kim , P. J. Attayek , Y. Wang , K. L. Furtado , R. Tamayo , C. E. Sims , N. L. Allbritton , Biofabrication 2020, 12, 015006. 10.1088/1758-5090/ab446e PMC6933551 31519008

[228] N. Sasaki , K. Miyamoto , K. M. Maslowski , H. Ohno , T. Kanai , T. Sato , Gastroenterology 2020, 159, 388. 32199883 10.1053/j.gastro.2020.03.021

[229] C. Zhao , R. Liu , Y. Zhou , R. Zheng , Y. Shen , B. Wen , B. Zhang , J. Che , Biomed. Technol. 2023, 3, 1.

[230] S. K. Ramakrishnan , Y. M. Shah , Annu. Rev. Inc. 2016, 78, 301. 10.1146/annurev-physiol-021115-105202 PMC4809193 26667076

[231] Y. S. Son , S. J. Ki , R. Thanavel , J. J. Kim , M. O. Lee , J. Kim , C. R. Jung , T. S. Han , H. S. Cho , C. M. Ryu , S. H. Kim , D. S. Park , M. Y. Son , FASEB J. 2020, 34, 9899. 32602623 10.1096/fj.202000063R

[232] M. A. Engevik , M. B. Yacyshyn , K. A. Engevik , J. Wang , B. Darien , D. J. Hassett , B. R. Yacyshyn , R. T. Worrell , Am. J. Physiol. 2014, 308, G510. 10.1152/ajpgi.00091.2014 PMC4422372 25552581

[233] J. Petersson , O. Schreiber , G. C. Hansson , S. J. Gendler , A. Velcich , J. O. Lundberg , S. Roos , L. Holm , M. Phillipson , Am. J. Physiol. 2011, 300, G327. 10.1152/ajpgi.00422.2010 PMC3302190 21109593

[234] Y. Wang , R. Kim , C. E. Sims , N. L. Allbritton , Cell. Mol. Gastroenterol. Hepatol. 2019, 8, 653. 31356887 10.1016/j.jcmgh.2019.07.009 PMC6889783

[235] M. Noben , B. Verstockt , M. de Bruyn , N. Hendriks , G. Van Assche , S. Vermeire , C. Verfaillie , M. Ferrante , Gut 2017, 66, 2193. 28159838 10.1136/gutjnl-2016-313667

[236] M. Vancamelbeke , T. Laeremans , W. Vanhove , K. Arnauts , A. S. Ramalho , R. Farré , I. Cleynen , M. Ferrante , S. Vermeire , J. Crohns Colitis 2019, 13, 1351. 30919886 10.1093/ecco-jcc/jjz064 PMC6764103

[237] A. H. Sachdev , M. Pimentel , Ther. Adv. Chronic Dis. 2013, 4, 223. 23997926 10.1177/2040622313496126 PMC3752184

[238] M. Pimentel , E. J. Chow , H. C. Lin , Am. J. Gastroenterol. 2000, 95, 3503. 11151884 10.1111/j.1572-0241.2000.03368.x

[239] Y. Funayama , I. Sasaki , H. Naito , K. Fukushima , C. Shibata , T. Masuko , K. I. Takahashi , H. Ogawa , S. Sato , T. Ueno , M. Noguchi , N. Hiwatashi , S. Matsuno , Dis. Colon Rectum 1999, 42, 1072. 10458133 10.1007/BF02236706

[240] X. Qian , H. N. Nguyen , M. M. Song , C. Hadiono , S. C. Ogden , C. Hammack , B. Yao , G. R. Hamersky , F. Jacob , C. Zhong , K. J. Yoon , W. Jeang , L. Lin , Y. Li , J. Thakor , D. A. Berg , C. Zhang , E. Kang , M. Chickering , D. Nauen , C. Y. Ho , Z. Wen , K. M. Christian , P. Y. Shi , B. J. Maher , H. Wu , P. Jin , H. Tang , H. Song , G. L. Ming , Cell 2016, 165, 1238. 27118425 10.1016/j.cell.2016.04.032 PMC4900885

[241] W. Shin , C. D. Hinojosa , D. E. Ingber , H. J. Kim , iScience 2019, 15, 391. 31108394 10.1016/j.isci.2019.04.037 PMC6526295

[242] Y. Wang , R. Kim , D. B. Gunasekara , M. I. Reed , M. DiSalvo , D. L. Nguyen , S. J. Bultman , C. E. Sims , S. T. Magness , N. L. Allbritton , Cell. Mol. Gastroenterol. Hepatol. 2018, 5, 113. 29693040 10.1016/j.jcmgh.2017.10.007 PMC5904049

[243] W. Shin , H. J. Kim , Nat. Protoc. 2022, 17, 910. 35110737 10.1038/s41596-021-00674-3 PMC9675318

[244] C. Moysidou , A. M. Withers , A. J. Nisbet , D. R. G. Price , C. E. Bryant , C. Cantacessi , R. M. Owens , Adv Biol 2022, 6, 2200015. 10.1002/adbi.202200015 35652159

[245] Y. G. Zhang , S. Wu , Y. Xia , J. Sun , Physiol. Rep. 2014, 2, 12147. 10.14814/phy2.12147 PMC4270227 25214524

[246] M. Hamon , H. Bierne , P. Cossart , Nat. Rev. Microbiol. 2006, 4, 423. 16710323 10.1038/nrmicro1413

[247] C. Klotz , T. Aebischer , F. Seeber , Int. J. Med. Micorbiol. 2012, 302, 203. 10.1016/j.ijmm.2012.07.010 22898491

[248] C. Pleguezuelos‐Manzano , J. Puschhof , A. Rosendahl Huber , A. van Hoeck , H. M. Wood , J. Nomburg , C. Gurjao , F. Manders , G. Dalmasso , P. B. Stege , F. L. Paganelli , M. H. Geurts , J. Beumer , T. Mizutani , Y. Miao , R. van der Linden , S. van der Elst , J. C. Ambrose , P. Arumugam , E. L. Baple , M. Bleda , F. Boardman‐Pretty , J. M. Boissiere , C. R. Boustred , H. Brittain , M. J. Caulfield , G. C. Chan , C. E. H. Craig , L. C. Daugherty , A. de Burca , et al., Nature 2020, 580, 269.32106218

[249] S. Bartfeld , H. Clevers , J. Vis. Exp. 2015, 2015, 53359. 10.3791/53359 PMC4692704 26650279

[250] J. Puschhof , C. Pleguezuelos‐Manzano , A. Martinez‐Silgado , N. Akkerman , A. Saftien , C. Boot , A. de Waal , J. Beumer , D. Dutta , I. Heo , H. Clevers , Nat. Protoc. 2021, 16, 4633. 34381208 10.1038/s41596-021-00589-z

[251] G. E. Kaiko , S. H. Ryu , O. I. Koues , P. L. Collins , L. Solnica‐Krezel , E. J. Pearce , E. L. Pearce , E. M. Oltz , T. S. Stappenbeck , Cell 2016, 165, 1708. 27264604 10.1016/j.cell.2016.05.018 PMC5026192

[252] I. A. Williamson , J. W. Arnold , L. A. Samsa , L. Gaynor , M. DiSalvo , J. L. Cocchiaro , I. Carroll , M. A. Azcarate‐Peril , J. F. Rawls , N. L. Allbritton , S. T. Magness , Cell. Mol. Gastroenterol. Hepatol. 2018, 6, 301. 30123820 10.1016/j.jcmgh.2018.05.004 PMC6092482

[253] J. Y. Co , M. Margalef‐Català , X. Li , A. T. Mah , C. J. Kuo , D. M. Monack , M. R. Amieva , Cell Rep. 2019, 26, 2509. 30811997 10.1016/j.celrep.2019.01.108 PMC6391775

[254] Y. Yokoi , K. Nakamura , T. Yoneda , M. Kikuchi , R. Sugimoto , Y. Shimizu , T. Ayabe , Sci. Rep. 2019, 9, 2710. 30804449 10.1038/s41598-019-39610-7 PMC6389922

[255] S. S. Wilson , A. Tocchi , M. K. Holly , W. C. Parks , J. G. Smith , Mucosal Immunol. 2015, 8, 352. 25118165 10.1038/mi.2014.72 PMC4326599

[256] G. Nigro , R. Rossi , P. H. Commere , P. Jay , P. J. Sansonetti , Cell Host Microbe 2014, 15, 792. 24882705 10.1016/j.chom.2014.05.003

[257] C. Zhou , Y. Zou , J. Huang , Z. Zhao , Y. Zhang , Y. Wei , K. Ye , Int. J. Mol. Sci. 2022, 23, 6231. 35682909 10.3390/ijms23116231 PMC9181811

[258] L. L. Clarke , Am. J. Physiol. 2009, 296, 1151. 10.1152/ajpgi.90649.2008 PMC2697950 19342508

[259] I. Schoultz , Å. V. Keita , Cells 2020, 9, 1909. 32824536 10.3390/cells9081909 PMC7463717

[260] N. V. Jafari , S. A. Kuehne , N. P. Minton , E. Allan , M. Bajaj‐Elliott , Anaerobe 2016, 37, 96. 26708704 10.1016/j.anaerobe.2015.12.007

[261] J. C. Eby , W. P. Ciesla , W. Hamman , G. M. Donato , R. J. Pickles , E. L. Hewlett , W. I. Lencer , J. Biol. Chem. 2010, 285, 10662. 20139088 10.1074/jbc.M109.089219 PMC2856274

[262] D. Huh , H. J. Kim , J. P. Fraser , D. E. Shea , M. Khan , A. Bahinski , G. A. Hamilton , D. E. Ingber , Nat. Protoc. 2013, 8, 2135. 24113786 10.1038/nprot.2013.137

[263] R. D. Singh , M. Khullar , N. K. Ganguly , Mol. Cell. Biochem. 2000, 215, 39. 11204454 10.1023/a:1026545630773

[264] S. Jalili‐Firoozinezhad , F. S. Gazzaniga , E. L. Calamari , D. M. Camacho , C. W. Fadel , A. Bein , B. Swenor , B. Nestor , M. J. Cronce , A. Tovaglieri , O. Levy , K. E. Gregory , D. T. Breault , J. M. S. Cabral , D. L. Kasper , R. Novak , D. E. Ingber , Nat. Biomed. Eng. 2019, 3, 520. 31086325 10.1038/s41551-019-0397-0 PMC6658209

[265] H. J. Kim , D. Huh , G. Hamilton , D. E. Ingber , Lab Chip 2012, 12, 2165. 22434367 10.1039/c2lc40074j

[266] M. Verhulsel , A. Simon , M. Bernheim‐Dennery , V. R. Gannavarapu , L. Gérémie , D. Ferraro , D. Krndija , L. Talini , J.‐L. Viovy , D. M. Vignjevic , S. Descroix , Lab Chip 2021, 21, 365. 33306083 10.1039/d0lc00672f PMC9930731

[267] Y. Wang , D. B. Gunasekara , M. I. Reed , M. DiSalvo , S. J. Bultman , C. E. Sims , S. T. Magness , N. L. Allbritton , Biomaterials 2017, 128, 44. 28288348 10.1016/j.biomaterials.2017.03.005 PMC5392043

[268] J. Creff , R. Courson , T. Mangeat , J. Foncy , S. Souleille , C. Thibault , A. Besson , L. Malaquin , Biomaterials 2019, 221, 119404. 31419651 10.1016/j.biomaterials.2019.119404

[269] K. I. Park , Y. D. Teng , E. Y. Snyder , Nat. Biotechnol. 2002, 20, 1111. 12379868 10.1038/nbt751

[270] S. Halstenberg , A. Panitch , S. Rizzi , H. Hall , J. A. Hubbell , Biomacromolecules 2002, 3, 710. 12099815 10.1021/bm015629o

[271] N. Gjorevski , N. Sachs , A. Manfrin , S. Giger , M. E. Bragina , P. Ordóñez‐Morán , H. Clevers , M. P. Lutolf , Nature 2016, 539, 560. 27851739 10.1038/nature20168

[272] J. D. Wang , N. J. Douville , S. Takayama , M. ElSayed , Ann. Biomed. Eng. 2012, 40, 1862. 22484830 10.1007/s10439-012-0562-z

[273] C. M. Leung , P. de Haan , K. Ronaldson‐Bouchard , G.‐A. Kim , J. Ko , H. S. Rho , Z. Chen , P. Habibovic , N. L. Jeon , S. Takayama , M. L. Shuler , G. Vunjak‐Novakovic , O. Frey , E. Verpoorte , Y.‐C. Toh , Nat Rev Methods Primers 2022, 2, 33.

[274] E. Berthier , E. W. K. Young , D. Beebe , Lab Chip 2012, 12, 1224. 22318426 10.1039/c2lc20982a

[275] E. Sackmann , Science 1996, 271, 43. 8539599 10.1126/science.271.5245.43

[276] M. Tanaka , E. Sackmann , Nature 2005, 437, 656. 16193040 10.1038/nature04164

[277] H. Y. Liu , H. Grant , H. L. Hsu , R. Sorkin , F. Bošković , G. Wuite , S. Daniel , ACS Appl. Mater. Interfaces 2017, 9, 35526. 28930438 10.1021/acsami.7b07500

[278] M. Tanaka , E. Sackmann , Phys. Status Solidi A 2006, 203, 3452.

[279] E. T. Castellana , P. S. Cremer , Surf. Sci. Rep. 2006, 61, 429. 32287559 10.1016/j.surfrep.2006.06.001 PMC7114318

[280] S. J. Singer , G. L. Nicolson , Science 1972, 175, 720. 4333397 10.1126/science.175.4023.720

[281] R. P. Richter , R. Bérat , A. R. Brisson , Langmuir 2006, 22, 3497. 16584220 10.1021/la052687c

[282] L. K. Tamm , H. M. McConnell , Biophys. J. 1985, 47, 105. 3978184 10.1016/S0006-3495(85)83882-0 PMC1435076

[283] H. Su , H. Y. Liu , A. M. Pappa , T. C. Hidalgo , P. Cavassin , S. Inal , R. M. Owens , S. Daniel , ACS Appl. Mater. Interfaces 2019, 11, 43799. 31659897 10.1021/acsami.9b10303

[284] J. Nissen , S. Gritsch , G. Wiegand , J. O. Rädler , Eur. Phys. J. B 1999, 10, 335.

[285] R. Richter , A. Mukhopadhyay , A. Brisson , Biophys. J. 2003, 85, 3035. 14581204 10.1016/S0006-3495(03)74722-5 PMC1303580

[286] M. Tanaka , F. F. Rossetti , S. Kaufmann , Biointerphases 2008, 3, FA12. 20408661 10.1116/1.2905233

[287] L. Wessels , M. W. Elting , D. Scimeca , K. Weninger , Biophys. J. 2007, 93, 526. 17449662 10.1529/biophysj.106.097485 PMC1896232

[288] A. K. Jayaram , A. M. Pappa , S. Ghosh , Z. A. Manzer , W. C. Traberg , T. P. J. Knowles , S. Daniel , R. M. Owens , Trends Biotechnol. 2022, 40, 107. 34229865 10.1016/j.tibtech.2021.06.001

[289] M. Tanaka , MRS Bull. 2006, 31, 513.

[290] E. Sackmann , M. Tanaka , Trends Biotechnol. 2000, 18, 58. 10652510 10.1016/s0167-7799(99)01412-2

[291] K. Bali , C. Guffick , R. McCoy , Z. Lu , C. F. Kaminski , I. Mela , R. M. Owens , H. W. van Veen , ACS Appl. Mater. Interfaces 2023, 15, 12766. 36866935 10.1021/acsami.2c21556 PMC10020959

[292] Z. Lu , D. van Niekerk , A. Savva , K. Kallitsis , Q. Thiburce , A. Salleo , A. M. Pappa , R. M. Owens , J. Mater. Chem. C. 2022, 10, 8050.

[293] K. Bali , R. McCoy , Z. Lu , J. Treiber , A. Savva , C. F. Kaminski , G. Salmond , A. Salleo , I. Mela , R. Monson , R. M. Owens , ACS Biomater. Sci. Eng. 2023, 9, 3632. 37137156 10.1021/acsbiomaterials.3c00021 PMC10265573

[294] B. Geny , M. R. Popoff , Biol. Cell. 2006, 98, 667. 17042742 10.1042/BC20050082

[295] J. M. Moran‐Mirabal , J. B. Edel , G. D. Meyer , D. Throckmorton , A. K. Singh , H. G. Craighead , Biophys. J. 2005, 89, 296. 15833994 10.1529/biophysj.104.054346 PMC1366527

[296] S. P. Hardy , T. Lund , P. E. Granum , FEMS Microbiol. Lett. 2006, 197, 47. 10.1111/j.1574-6968.2001.tb10581.x 11287145

[297] H. Jung , A. D. Robison , P. S. Cremer , J. Struct. Biol. 2009, 168, 90. 19508894 10.1016/j.jsb.2009.05.010 PMC2752984

[298] M. Bally , G. E. Rydell , R. Zahn , W. Nasir , C. Eggeling , M. E. Breimer , L. Svensson , F. Höök , G. Larson , Angew. Chem., Int. Ed. 2012, 51, 12020. 10.1002/anie.201205972 PMC3546384 23097253

[299] G. Marasco , M. V. Lenti , C. Cremon , M. R. Barbaro , V. Stanghellini , A. Di Sabatino , G. Barbara , Neurogastroenterol. Motil. 2021, 33, 14104. 10.1111/nmo.14104 PMC7995160 33591607

[300] K. Kallitsis , A. Pappa , Z. Lu , A. Alvarez‐Fernandez , I. Charalambous , S. Schack , W. C. Traberg , Q. Thiburce , K. Bali , G. Christie , S. Guldin , S. Daniel , A. Salleo , R. M. Owens , Macromol. Mater. Eng. 2023, 308, 2300038.

[301] A. Luchini , S. Micciulla , G. Corucci , K. C. Batchu , A. Santamaria , V. Laux , T. Darwish , R. A. Russell , M. Thepaut , I. Bally , F. Fieschi , G. Fragneto , Sci. Rep. 2021, 11, 14867. 34290262 10.1038/s41598-021-93996-x PMC8295359

[302] L.‐Q. Gu , O. Braha , S. Conlan , S. Cheley , H. Bayley , Nature 1999, 398, 686. 10227291 10.1038/19491

[303] R. Bansil , B. S. Turner , Adv Drug Deliv Rev 2018, 124, 3. 28970050 10.1016/j.addr.2017.09.023

[304] L. Sardelli , D. P. Pacheco , A. Ziccarelli , M. Tunesi , O. Caspani , A. Fusari , F. B. Vangosa , P. Petrini , 2019, 9, 15887. 10.1039/c9ra02368b PMC9064393 35521409

[305] P. Paone , P. D. Cani , Gut 2020, 69, 2232. 32917747 10.1136/gutjnl-2020-322260 PMC7677487

[306] A. P. Moran , A. Gupta , L. Joshi , Gut 2011, 60, 1412. 21228430 10.1136/gut.2010.212704

[307] C. Werlang , G. Cárcarmo‐Oyarce , K. Ribbeck , Nat. Rev. Mater. 2019, 4, 134. 10.1038/s41578-018-0079-7 PMC11906034 40084234

[308] M. E. V. Johansson , G. C. Hansson , Nat. Rev. Immunol. 2016, 16, 639. 27498766 10.1038/nri.2016.88 PMC6435297

[309] D. J. Thornton , K. Rousseau , M. A. McGuckin , Annu. Rev. Physiol. 2008, 70, 459. 17850213 10.1146/annurev.physiol.70.113006.100702

[310] S. Trillo‐muyo , H. E. Nilsson , C. V Recktenwald , A. Ermund , C. Ridley , L. N. Meiss , A. Bähr , N. Klymiuk , J. J. Wine , P. J. B. Koeck , D. J. Thornton , H. Hebert , G. C. Hansson , J. Biol. Chem. 2018, 293, 5746. 29440393 10.1074/jbc.RA117.001014 PMC5900763

[311] G. Javitt , L. Khmelnitsky , L. Albert , Y. Levy , R. Diskin , G. Javitt , L. Khmelnitsky , L. Albert , L. S. Bigman , N. Elad , D. Morgenstern , T. Ilani , Cell 2020, 183, 717. 33031746 10.1016/j.cell.2020.09.021 PMC7599080

[312] A. Ermund , A. Schütte , M. E. V Johansson , J. K. Gustafsson , G. C. Hansson , Am. J. Physiol. 2023, 305, 341. 10.1152/ajpgi.00046.2013 PMC3761247 23832518

[313] G. Birchenough , M. Johansson , J. Gustafsson , J. Bergstrom , G. Hansson , Soc. Mucosal Immunol. 2015, 8, 712. 10.1038/mi.2015.32 PMC4631840 25872481

[314] C. Zhao , L. Cai , H. Chen , H. Tan , D. Yan , Eng. Regener. 2021, 2, 116.

[315] R. et al Lennernäs , H. Ahrenstedt , Ö. Hällgren , Pharm. Res. 1992, 9, 1243. 1448420 10.1023/a:1015888813741

[316] B. Lukanc , A. N. Svete , Slov. Vet. Res. 2017, 54, 117.

[317] R. T. Osiecka , I. Porter , P. A. Borchardt , Pharm. Res. 1985, 9, 284. 10.1023/A:1016341601273 24271125

[318] P. S. Leppert , J. A. Fix , J. Pharm. Sci. 1993, 83, 976. 10.1002/jps.2600830712 7965678

[319] O. Lieleg , C. Lieleg , J. Bloom , C. B. Buck , K. Ribbeck , Biomacromolecules 2012, 13, 1724. 22475261 10.1021/bm3001292 PMC3597216

[320] D. Song , E. Iverson , L. Kaler , S. Bader , M. A. Scull , G. A. Duncan , ACS Biomater. Sci. Eng. 2021, 7, 2723. 33871978 10.1021/acsbiomaterials.0c01728 PMC8803127

[321] L. Sardelli , F. Briatico , M. Merli , A. Ziccarelli , S. Visentin , L. Visai , P. Petrini , Biomater. Adv. 2022, 139, 213022. 35891596 10.1016/j.bioadv.2022.213022

[322] C. Hilgendorf , H. Spahn‐Langguth , C. G. Regardh , E. Lipka , G. L. Amidon , P. Langguth , J. Pharm. Sci. 2000, 89, 63. 10664539 10.1002/(SICI)1520-6017(200001)89:1<63::AID-JPS7>3.0.CO;2-6

[323] R. T. Borchardt , Gastroenterology 1989, 96, 736.2914637

[324] R. Bej , R. Haag , J. Am. Chem. Soc. 2022, 144, 20137. 36074739 10.1021/jacs.1c13547 PMC9650700

[325] R. Hamed , J. Fiegel , J. Biomed. Mater. Res. 2013, 102, 1788. 10.1002/jbm.a.34851 PMC4719568 23813841

[326] K. Joyner , D. Song , R. F. Hawkins , R. D. Silcott , G. A. Duncan , Soft Matter 2019, 15, 9632. 31651920 10.1039/c9sm01715a

[327] N. L. Kavanaugh , A. Q. Zhang , C. J. Nobile , A. D. Johnson , K. Ribbeck , mBio 2014, 5, 01911. 10.1128/mBio.01911-14 PMC4235211 25389175

[328] A. Macierzanka , A. R. Mackie , L. Krupa , Sci. Rep. 2019, 9, 17516. 31772308 10.1038/s41598-019-53933-5 PMC6879640

[329] J. Sotres , S. Jankovskaja , K. Wannerberger , T. Arnebrant , Sci. Rep. 2017, 7, 7270. 28779181 10.1038/s41598-017-07552-7 PMC5544714

[330] L. Wright , P. Joyce , T. J. Barnes , C. A. Prestidge , ACS Biomater. Sci. Eng. 2021, 9, 2819. 34784462 10.1021/acsbiomaterials.1c00814

[331] A. Schütte , A. Ermund , C. Becker‐pauly , M. E. V Johansson , A. M. Rodriguez‐pineiro , Proc. Natl. Acad. Sci. USA 2014, 111, 2. 10.1073/pnas.1407597111 PMC4151749 25114233

[332] H. E. Jakobsson , A. M. Rodríguez‐piñeiro , A. Schütte , A. Ermund , P. Boysen , M. Bemark , F. Sommer , F. Bäckhed , G. C. Hansson , M. E. V Johansson , EMBO Rep. 2015, 16, 164. 25525071 10.15252/embr.201439263 PMC4328744

[333] B. O. Schroeder , Gastroenterol Rep (Oxf) 2019, 7, 3.30792861

[334] D. R. Mack , S. Michail , S. H. U. Wei , L. M. C. Dougall , M. A. Hollingsworth , R. David , S. Michail , S. Wei , L. Mc , M. A. H. Probiotics , Am. J. Physiol. 1999, 276, 941.

[335] G. P. Donaldson , S. M. Lee , S. K. Mazmanian , Nat. Rev. Microbiol. 2016, 14, 20. 26499895 10.1038/nrmicro3552 PMC4837114

[336] L. Etienne‐Mesmin , B. Chassaing , M. Desvaux , et al., FEMS Microbiol. Rev. 2019, 43, 457. 31162610 10.1093/femsre/fuz013

[337] G. C. Hansson , J. Intern. Med. 2019, 285, 479. 30963635 10.1111/joim.12910 PMC6497544

[338] Y. Bertin , F. Chaucheyras‐durand , C. Robbe‐masselot , A. Durand , A. De Foye , J. Harel , P. S. Cohen , T. Conway , E. Forano , C. Martin , Environ. Microbiol. 2013, 15, 610. 23126484 10.1111/1462-2920.12019 PMC3558604

[339] J. Wurpel , M. Totsika , L. P. Allsopp , L. E. Hartley‐tassell , C. J. Day , K. M. Peters , S. Sarkar , G. C. Ulett , J. Yang , J. Tiralongo , R. A. Strugnell , M. P. Jennings , M. A. Schembri , PLoS One 2014, 9, 1. 10.1371/journal.pone.0093177 PMC3966885 24671091

[340] B. Srinivasan , A. R. Kolli , M. B. Esch , H. E. Abaci , M. L. Shuler , J. J. Hickman , J. Lab Autom. 2015, 20, 107. 25586998 10.1177/2211068214561025 PMC4652793

[341] S. Lopez‐Escalera , A. Wellejus , Biochem. Biophys. Rep. 2022, 31, 101314. 35873654 10.1016/j.bbrep.2022.101314 PMC9304606

[342] R. El Asmar , P. Panigrahi , P. Bamford , I. Berti , T. Not , G. V. Coppa , C. Catassi , A. Fasano , Gastroenterology 2002, 123, 1607. 12404235 10.1053/gast.2002.36578

[343] M. M. Cajnko , M. Marušić , M. Kisovec , N. Rojko , M. Benčina , S. Caserman , G. Anderluh , PLoS One 2015, 10, 0130471. 10.1371/journal.pone.0130471 PMC4472510 26087154

[344] H. W. Fang , S. Bin Fang , J. S. C. Chiau , C. Y. Yeung , W. T. Chan , C. Bin Jiang , M. L. Cheng , H. C. Lee , J. Med. Microbiol. 2010, 59, 573. 20110387 10.1099/jmm.0.009662-0

[345] R. C. Anderson , A. L. Cookson , W. C. McNabb , Z. Park , M. J. McCann , W. J. Kelly , N. C. Roy , BMC Microbiol. 2010, 10, 1. 21143932 10.1186/1471-2180-10-316 PMC3004893

[346] T. Gerasimenko , S. Nikulin , G. Zakharova , A. Poloznikov , V. Petrov , A. Baranova , A. Tonevitsky , Front. Bioeng. Biotechnol. 2020, 7, 474. 32039179 10.3389/fbioe.2019.00474 PMC6992543

[347] D. D. MacDonald , Electrochim. Acta 2006, 51, 1376.

[348] I. Giaever , C. R. Keese , Proc. Natl. Acad. Sci. USA 1991, 88, 7896. 1881923 10.1073/pnas.88.17.7896 PMC52411

[349] Z. Maherally , H. L. Fillmore , S. L. Tan , S. F. Tan , S. A. Jassam , F. I. Quack , K. E. Hatherell , G. J. Pilkington , FASEB J. 2018, 32, 168. 28883042 10.1096/fj.201700162R PMC5731124

[350] R. Szulcek , H. J. Bogaard , G. P. van Nieuw Amerongen , J. Vis. Exp. 2014, 85, 51300. 10.3791/51300 PMC4159052 24747269

[351] M. Amini , J. Hisdal , H. Kalvøy , J. Electr. Bioimpedance 2018, 9, 142. 33584930 10.2478/joeb-2018-0019 PMC7852004

[352] C. Moysidou , C. Pitsalidis , M. Al‐Sharabi , A. M. Withers , J. A. Zeitler , R. M. Owens , Adv. Biol. 2021, 5, 2000306.

[353] M. Marziano , S. Tonello , E. Cantù , G. Abate , M. Vezzoli , W. Rungratanawanich , M. Serpelloni , N. F. Lopomo , M. Memo , E. Sardini , D. Uberti , Biochim. Biophys. Acta, Gen. Subj. 2019, 1863, 893. 30817979 10.1016/j.bbagen.2019.02.008

[354] H.‐Y. Tan , S. Trier , U. L. Rahbek , M. Dufva , J. P. Kutter , T. L. Andresen , PLoS One 2018, 13, 0197101. 10.1371/journal.pone.0197101 PMC5944968 29746551

[355] A. Nicolas , F. Schavemaker , K. Kosim , D. Kurek , M. Haarmans , M. Bulst , K. Lee , S. Wegner , T. Hankemeier , J. Joore , K. Domansky , H. L. Lanz , P. Vulto , S. J. Trietsch , Lab Chip 2021, 21, 1676. 33861225 10.1039/d0lc00770f

[356] J. Rivnay , M. Ramuz , P. Leleux , A. Hama , M. Huerta , R. M. Owens , Appl. Phys. Lett. 2015, 106, 55.

[357] K. Lieberth , A. Pavlou , D. Harig , P. W. M. Blom , P. Gkoupidenis , F. Torricelli , Adv. Mater. Technol. 2023, 8, 2201697.

[358] F. Bonafè , F. Decataldo , I. Zironi , D. Remondini , T. Cramer , B. Fraboni , Nat. Commun. 2022, 13, 1. 36109508 10.1038/s41467-022-33094-2 PMC9477811

[359] D. Ulluwishewa , R. C. Anderson , W. Young , W. C. Mcnabb , P. van Baarlen , P. J. Moughan , J. M. Wells , N. C. Roy , Cell. Microbiol. 2015, 17, 226. 25224879 10.1111/cmi.12360

[360] W. W. Stewart , Nature 1981, 292, 17. 6168915 10.1038/292017a0

[361] Å. Keita , S. Salim , T. Jiang , P.‐C. Yang , L. Franzén , P. Söderkvist , K.‐E. Magnusson , J. Söderholm , J Pathol 2008, 215, 135. 18348161 10.1002/path.2337

[362] C. Tagesson , R. Sjödahl , B. Thorén , Scand J Gastroenterol 1978, 13, 519. 705246 10.3109/00365527809181758

[363] T. Lindmark , Y. Kimura , P. Artursson , J. Pharmacol. Expe. Ther. 1998, 284, 362. 9435199

[364] P. Parajuli , K. Gokulan , S. Khare , Int. J. Mol. Sci. 2022, 23, 4851. 35563241 10.3390/ijms23094851 PMC9101442

[365] N. Uchide , K. Ohyama , T. Bessho , H. Toyoda , Intervirology 2009, 52, 164. 19521105 10.1159/000224644

[366] L. Wang , C. Llorente , P. Hartmann , A.‐M. Yang , P. Chen , B. Schnabl , J. Immunol. Methods 2015, 421, 44. 25595554 10.1016/j.jim.2014.12.015 PMC4451427

[367] A. Thomson , K. Smart , M. S. Somerville , S. N. Lauder , G. Appanna , J. Horwood , L. Sunder Raj , B. Srivastava , D. Durai , M. J. Scurr , Å. V. Keita , A. M. Gallimore , A. Godkin , BMC Gastroenterol. 2019, 19, 98. 31221083 10.1186/s12876-019-1002-4 PMC6585111

[368] A. V. Keita , J. D. Söderholm , Neurogastroenterol. Motil. 2010, 22, 718. 20377785 10.1111/j.1365-2982.2010.01498.x

[369] A. Boquet‐Pujadas , T. Feaugas , A. Petracchini , A. Grassart , H. Mary , M. Manich , S. Gobaa , J. C. Olivo‐Marin , N. Sauvonnet , E. Labruyère , Sci. Adv. 2022, 8, abo5767. 10.1126/sciadv.abo5767 PMC9586479 36269830

[370] J. Roostalu , A. Jõers , H. Luidalepp , N. Kaldalu , T. Tenson , BMC Microbiol. 2008, 8, 68. 18430255 10.1186/1471-2180-8-68 PMC2377270

[371] S. Helaine , J. A. Thompson , K. G. Watson , M. Liu , C. Boyle , D. W. Holden , Proc. Natl. Acad. Sci. USA 2010, 107, 3746. 20133586 10.1073/pnas.1000041107 PMC2840444

[372] X. Yin , H. F. Farin , J. H. Van Es , H. Clevers , R. Langer , J. M. Karp , Nat. Methods 2014, 11, 106. 24292484 10.1038/nmeth.2737 PMC3951815

[373] S. Salomé‐Desnoulez , S. Poiret , B. Foligné , G. Muharram , V. Peucelle , F. Lafont , C. Daniel , Gut microbes 2021, 13, 1. 10.1080/19490976.2021.1897374 PMC8009120 33779491

[374] R. Dheer , R. Santaolalla , J. M. Davies , J. K. Lang , M. C. Phillips , C. Pastorini , M. T. Vazquez‐Pertejo , M. T. Abreu , Infect. Immun. 2016, 84, 798. 26755160 10.1128/IAI.01374-15 PMC4771346

[375] L. L. Wu , W. H. Peng , W. T. Kuo , C. Y. Huang , Y. H. Ni , K. S. Lu , J. R. Turner , L. C. H. Yu , Am. J. Pathol. 2014, 184, 2260. 24911373 10.1016/j.ajpath.2014.05.003 PMC4188866

[376] S. Yu , I. Balasubramanian , D. Laubitz , K. Tong , S. Bandyopadhyay , X. Lin , J. Flores , R. Singh , Y. Liu , C. Macazana , Y. Zhao , F. Béguet‐Crespel , K. Patil , M. T. Midura‐Kiela , D. Wang , G. S. Yap , R. P. Ferraris , Z. Wei , E. M. Bonder , M. M. Häggblom , L. Zhang , V. Douard , M. P. Verzi , K. Cadwell , P. R. Kiela , N. Gao , Immunity 2020, 53, 398. 32814028 10.1016/j.immuni.2020.07.010 PMC7461615

[377] S. M. Crowley , X. Han , J. M. Allaire , M. Stahl , I. Rauch , L. A. Knodler , B. A. Vallance , PLoS Pathog. 2020, 16, 1008498. 10.1371/journal.ppat.1008498 PMC7179941 32282854

[378] L. Liu , W. Saitz‐Rojas , R. Smith , L. Gonyar , J. G. In , O. Kovbasnjuk , N. C. Zachos , M. Donowitz , J. P. Nataro , F. Ruiz‐Perez , Sci. Rep. 2020, 10, 10533. 32601325 10.1038/s41598-020-67104-4 PMC7324601

[379] A. T. Nielsen , N. A. Dolganov , G. Otto , M. C. Miller , Y. W. Cheng , G. K. Schoolnik , PLoS Pathog. 2006, 2, 109. 10.1371/journal.ppat.0020109 PMC1617127 17054394

[380] W. H. Jang , A. Park , T. Wang , C. J. Kim , H. Chang , B. G. Yang , M. J. Kim , S. J. Myung , S. H. Im , M. H. Jang , Y. M. Kim , K. H. Kim , Sci. Rep. 2018, 8, 14174. 30242205 10.1038/s41598-018-32640-7 PMC6155010

[381] K. M. Scherer , L. Mascheroni , G. W. Carnell , L. C. S. Wunderlich , S. Makarchuk , M. Brockhoff , I. Mela , A. Fernandez‐Villegas , M. Barysevich , H. Stewart , M. Suau Sans , C. L. George , J. R. Lamb , G. S. Kaminski‐Schierle , J. L. Heeney , C. F. Kaminski , Sci. Adv. 2022, 8, 4895. 10.1126/sciadv.abl4895 PMC10954198 34995113

[382] J. Schlegel , S. Peters , S. Doose , A. Schubert‐Unkmeir , M. Sauer , Front. Cell. Dev. Biol. 2019, 7, 194. 31572726 10.3389/fcell.2019.00194 PMC6753371

[383] S. J. L. Van Wijk , F. Fricke , L. Herhaus , J. Gupta , K. Hötte , F. Pampaloni , P. Grumati , M. Kaulich , Y. S. Sou , M. Komatsu , F. R. Greten , S. Fulda , M. Heilemann , I. Dikic , Nat. Microbiol. 2017, 2, 17066. 28481361 10.1038/nmicrobiol.2017.66

[384] M. J. Baker , J. Trevisan , P. Bassan , R. Bhargava , H. J. Butler , K. M. Dorling , P. R. Fielden , S. W. Fogarty , N. J. Fullwood , K. A. Heys , C. Hughes , P. Lasch , P. L. Martin‐Hirsch , B. Obinaju , G. D. Sockalingum , J. Sulé‐Suso , R. J. Strong , M. J. Walsh , B. R. Wood , P. Gardner , F. L. Martin , Nat. Protoc. 2014, 9, 1771. 24992094 10.1038/nprot.2014.110 PMC4480339

[385] J. Hanlan , D. A. Skoog , D. M. West , Stud. Conserv. 1973, 18, 45.

[386] K. Beton‐Mysur , B. Brozek‐Pluska , Molecules 2023,28, 137. 10.3390/molecules28010137 PMC9822473 36615330

[387] W. E. Huang , A. D. Ward , A. S. Whiteley , Environ. Microbiol. Rep. 2009, 1, 44. 23765719 10.1111/j.1758-2229.2008.00002.x

[388] A. Salman , U. Sharaha , E. Rodriguez‐Diaz , E. Shufan , K. Riesenberg , I. J. Bigio , M. Huleihel , Analyst 2017, 142, 2136. 28518194 10.1039/c7an00192d

[389] H. A. Bechtel , E. A. Muller , R. L. Olmon , M. C. Martin , M. B. Raschke , Proc. Natl. Acad. Sci. USA 2014, 111, 7191. 24803431 10.1073/pnas.1400502111 PMC4034206

[390] R. Goodacre , C. Lima , H. Muhamadali , Y. Xu , M. Kansiz , Anal. Chem. 2021, 93, 3082. 33522799 10.1021/acs.analchem.0c03967

[391] D. Zhang , C. Li , C. Zhang , M. N. Slipchenko , G. Eakins , J. X. Cheng , Sci. Adv. 2016, 2, 1600521. 10.1126/sciadv.1600521 PMC5040478 27704043

[392] B. Knoll , F. Keilmann , Nature 1999, 399, 134.

[393] T. Roodsant , M. Navis , I. Aknouch , I. B. Renes , R. M. van Elburg , D. Pajkrt , K. C. Wolthers , C. Schultsz , K. C. H. van der Ark , A. Sridhar , V. Muncan , Front. Cell. Infect. Microbiol. 2020, 10, 272. 32656095 10.3389/fcimb.2020.00272 PMC7326037

[394] A. Fritschen , A. K. Bell , I. Königstein , L. Stühn , R. W. Stark , A. Blaeser , Biomater. Sci. 2022, 10, 1981. 35262097 10.1039/d1bm01794b

[395] A. C. Croce , G. Bottiroli , Eur. J. Histochem. 2014, 58, 320. 10.4081/ejh.2014.2461 PMC4289852 25578980

[396] A. Sharaf , J. P. Frimat , G. J. Kremers , A. Accardo , Micro Nano Eng. 2023, 19, 100188.

[397] E. A. Franzosa , A. Sirota‐Madi , J. Avila‐Pacheco , N. Fornelos , H. J. Haiser , S. Reinker , T. Vatanen , A. B. Hall , H. Mallick , L. J. McIver , J. S. Sauk , R. G. Wilson , B. W. Stevens , J. M. Scott , K. Pierce , A. A. Deik , K. Bullock , F. Imhann , J. A. Porter , A. Zhernakova , J. Fu , R. K. Weersma , C. Wijmenga , C. B. Clish , H. Vlamakis , C. Huttenhower , R. J. Xavier , Nat. Microbiol. 2019, 4, 293. 30531976 10.1038/s41564-018-0306-4 PMC6342642

[398] The Integrative HMP (iHMP) Research Network Consortium . Cell Host Microbe 2014, 16, 276. 10.1016/j.chom.2014.08.014 PMC5109542 25211071

[399] A. Jacobson , L. Lam , M. Rajendram , F. Tamburini , J. Honeycutt , T. Pham , W. Van Treuren , K. Pruss , S. R. Stabler , K. Lugo , D. M. Bouley , J. G. Vilches‐Moure , M. Smith , J. L. Sonnenburg , A. S. Bhatt , K. C. Huang , D. Monack , Cell Host Microbe 2018, 24, 296. 30057174 10.1016/j.chom.2018.07.002 PMC6223613

[400] T. Hagi , S. Y. Geerlings , B. Nijsse , C. Belzer , Appl. Microbiol. Biotechnol. 2020, 104, 10641. 33159542 10.1007/s00253-020-10976-3 PMC7671984

[401] M. L. Reniere , A. T. Whiteley , K. L. Hamilton , S. M. John , P. Lauer , R. G. Brennan , D. A. Portnoy , Nature 2015, 517, 170. 25567281 10.1038/nature14029 PMC4305340

[402] F.‐P. J. Martin , N. Sprenger , I. K. S. Yap , Y. Wang , R. Bibiloni , F. Rochat , S. Rezzi , C. Cherbut , S. Kochhar , J. C. Lindon , E. Holmes , J. K. Nicholson , J. Proteome Res. 2009, 8, 2090. 19281268 10.1021/pr801068x

[403] M. Zimmermann , M. Kogadeeva , M. Gengenbacher , G. McEwen , H.‐J. Mollenkopf , N. Zamboni , S. H. E. Kaufmann , U. Sauer , mSystems 2017, 29, 10. 10.1128/mSystems.00057-17 PMC5566787 28845460

[404] M. Wang , J. J. Carver , V. V Phelan , L. M. Sanchez , N. Garg , Y. Peng , D. D. Nguyen , J. Watrous , C. A. Kapono , T. Luzzatto‐Knaan , C. Porto , A. Bouslimani , A. V Melnik , M. J. Meehan , W.‐T. Liu , M. Crüsemann , P. D. Boudreau , E. Esquenazi , M. Sandoval‐Calderón , R. D. Kersten , L. A. Pace , R. A. Quinn , K. R. Duncan , C.‐C. Hsu , D. J. Floros , R. G. Gavilan , K. Kleigrewe , T. Northen , R. J. Dutton , D. Parrot , et al., Nat. Biotechnol. 2016, 34, 828. 27504778 10.1038/nbt.3597 PMC5321674

[405] F. M. Orecchio , V. Tommaso , T. Santaniello , S. Castiglioni , F. Pezzotta , A. Monti , F. Butera , J. A. M. Maier , P. Milani , Micromachines (Basel) 2022, 13, 994. 35888811 10.3390/mi13070994 PMC9316907

[406] A. J. Stewart , E. J. O'Reilly , R. D. Moriarty , P. Bertoncello , T. E. Keyes , R. J. Forster , L. Dennany , Electrochim. Acta 2015, 157, 8.

[407] A. M. Pappa , O. Parlak , G. Scheiblin , P. Mailley , A. Salleo , R. M. Owens , Trends Biotechnol. 2018, 36, 45. 29196057 10.1016/j.tibtech.2017.10.022

[408] J. Oziat , T. Babin , M. Gougis , G. G. Malliaras , P. Mailley , Bioelectrochemistry 2023, 154, 108538. 37549554 10.1016/j.bioelechem.2023.108538

[409] F. Orecchio , V. Tommaso , T. Santaniello , S. Castiglioni , F. Pezzotta , A. Monti , F. Butera , J. Maier , P. Milani , Micromachines (Basel) 2022, 13, 994. 35888811 10.3390/mi13070994 PMC9316907

[410] S.‐S. D. Carter , A.‐R. Atif , S. Kadekar , I. Lanekoff , H. Engqvist , O. P. Varghese , M. Tenje , G. Mestres , Organs Chip 2020, 2, 100004.

[411] R. M. Owens , MRS Commun. 2023, 13, 685.

